# Rethinking Uncaging: A New Antiaromatic Photocage Driven by a Gain of Resonance Energy

**DOI:** 10.1002/chem.202102351

**Published:** 2021-09-02

**Authors:** Volker Hermanns, Maximilian Scheurer, Nils Frederik Kersten, Chahinez Abdellaoui, Josef Wachtveitl, Andreas Dreuw, Alexander Heckel

**Affiliations:** ^1^ Institute of Organic Chemistry and Chemical Biology Goethe University Frankfurt Max-von-Lau-Str. 7 60438 Frankfurt Germany; ^2^ Interdisciplinary Center for Scientific Computing Heidelberg University Im Neuenheimer Feld 205 69120 Heidelberg Germany; ^3^ Institute of Physical and Theoretical Chemistry Goethe University Frankfurt Max-von-Laue-Str. 7 60438 Frankfurt Germany

**Keywords:** Baird's rule, excited state aromaticity, photochemistry, photolabile protecting groups, substituent effects

## Abstract

Photoactivatable compounds for example photoswitches or photolabile protecting groups (PPGs, photocages) for spatiotemporal light control, play a crucial role in different areas of research. For each application, parameters such as the absorption spectrum, solubility in the respective media and/or photochemical quantum yields for several competing processes need to be optimized. The design of new photochemical tools therefore remains an important task. In this study, we exploited the concept of excited‐state‐aromaticity, first described by N. Colin Baird in 1971, to investigate a new class of photocages, based on cyclic, ground‐state‐antiaromatic systems. Several thio‐ and nitrogen‐functionalized compounds were synthesized, photochemically characterized and further optimized, supported by quantum chemical calculations. After choosing the optimal scaffold, which shows an excellent uncaging quantum yield of 28 %, we achieved a bathochromic shift of over 100 nm, resulting in a robust, well accessible, visible light absorbing, compact new photocage with a clean photoreaction and a high quantum product (ϵ⋅Φ) of 893 M^−1^ cm^−1^ at 405 nm.

## Graphical abstract

**The concept of** excited state aromaticity was used to design a new class of photolabile protecting groups, based on cyclic, ground‐state‐antiaromatic systems. Supported by theoretical calculations, several thio‐ and nitrogen functionalized compounds were synthesized, characterized and further optimized, resulting in a well accessible, visible light absorbing, new photocage with a high uncaging cross section of 893 M^−1^ cm^−1^ at 405 nm.

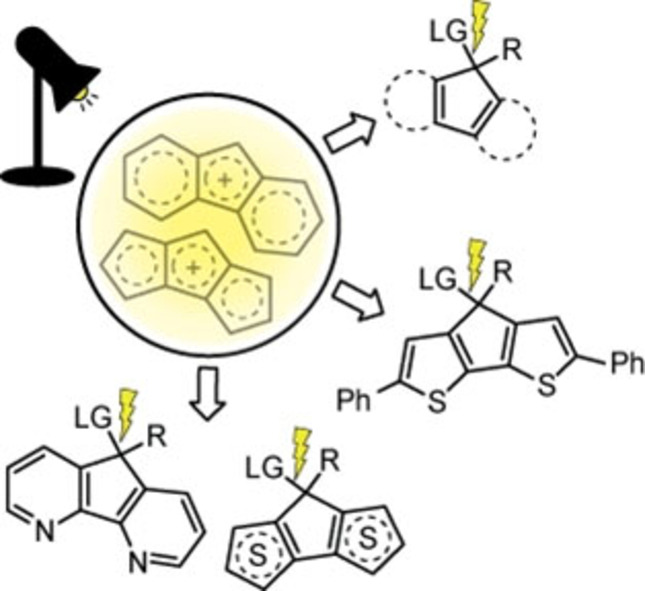

## Introduction

**Figure 1:**
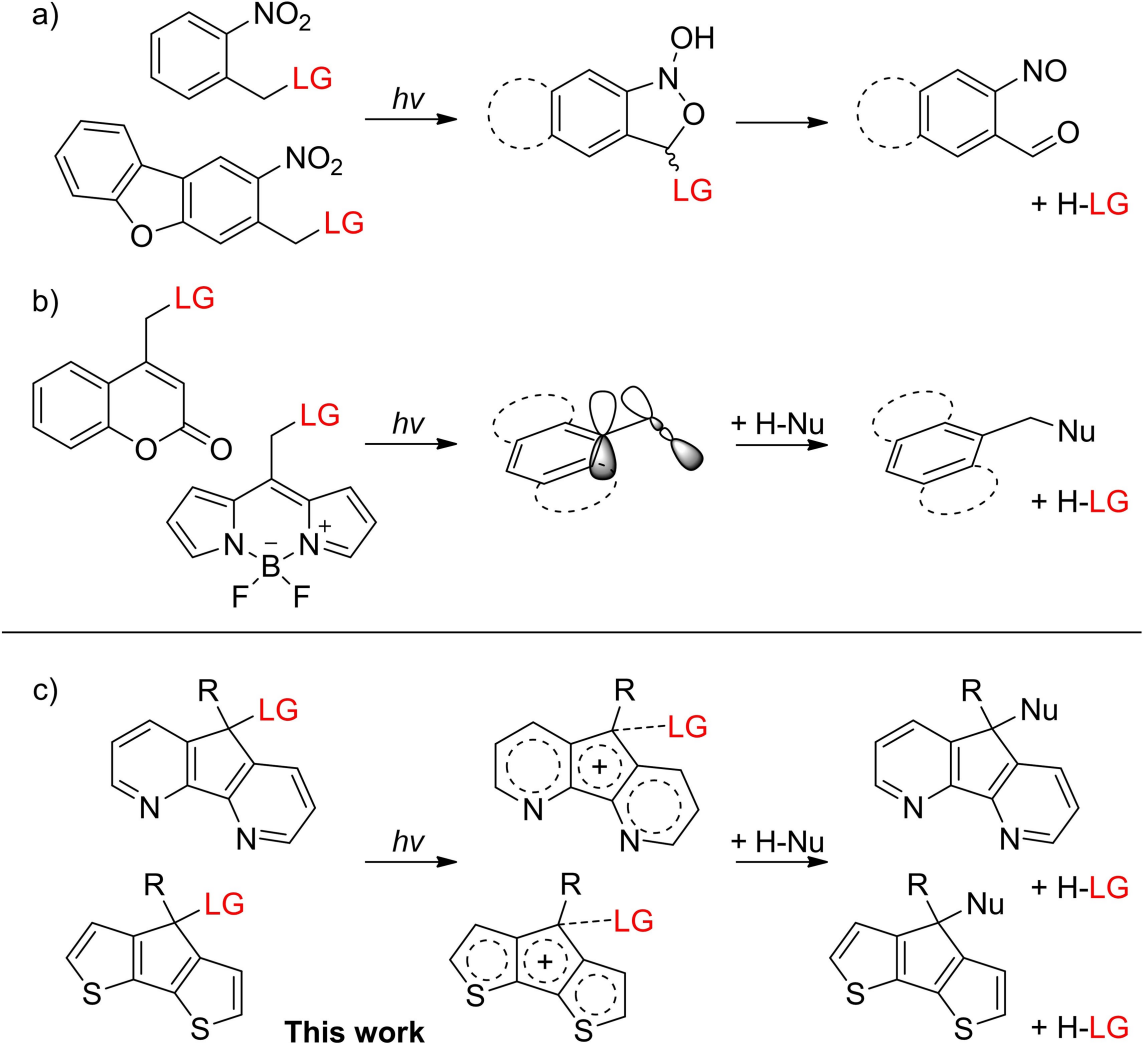
Uncaging concepts: a) nitrogen‐based push–pull systems (*o*NB, NDBF), which uncage via an *aci*‐nitro‐ and subsequent benzisoxazole intermediate, which is shown in the middle. b) Dye‐inspired systems (coumarin, BODIPY) with electron density donation into an antibonding molecular orbital. c) Excited‐state‐aromaticity assisted uncaging investigated here. (LG=leaving group, Nu=nucleophile, R=H, ethynyl, phenyl).

Photochemical processes are quite complex due to various competing reactions in the exited states. Besides many organic compounds, which react according to Kasha's rule^[1]^ there are several examples for non‐Kasha behavior^[2]^ or complex photoreactivity depending on the excitation wavelength,^[3]^ one‐ or two‐photon‐excitation^[4]^ or solvation in general.^[5]^ Deeper insight into these processes can be gained with computational methods, spectroscopically, with systematic synthetic work – or ideally with all of these approaches working together. The change of one parameter for example the solubility can have an impact on the excited state reactivity and therefore change the entire photochemistry.[^6^, ^7^, ^8^, ^9^] Hence, the design of new photoactivatable compounds needs to be based on elementary, but relevant concepts, as for example nitro‐push–pull‐systems or Zimmerman's *meta*‐effect (Figure 1a,b).[^10^, ^11^, ^12^] In the last decades, numerous new photocleavable protecting groups (PPGs, photocages) evolved from these existing concepts in photochemistry. Some examples are nitro‐based (*ortho*‐nitrobenzyl (*o*NB) and nitrodibenzofuran (NDBF)) or dye‐inspired (coumarin and boron‐dipyrromethene (BODIPY)), which have already been synthetically optimized and tested in biological systems.[^13^, ^14^, ^15^, ^16^, ^17^, ^18^] The initial photochemical process, which is essentially based on the push–pull idea for nitro‐based cages, is the formation of an *aci*‐nitro and subsequent benzisoxazole intermediate (Figure 1a).^[10]^ Coumarin‐ and BODIPY‐cages show an increase in electron density at the carbon atom next to the carbon atom attached to the leaving group (LG) upon irradiation.[^19^, ^20^]

This additional electron density – partially donated into the antibonding molecular orbital of the C−LG bond – weakens the strength of the C−LG bond, so that it can be cleaved off in a solvent‐assisted manner (Figure 1b). For the design of fundamentally new photocages, based on a robust principle, it is therefore necessary to re‐think the uncaging process in a new direction.

Herein we report a new class of photocages which are based on the excited‐state‐aromaticity concept of cyclic annulenes, first described by N. Colin Baird in 1971 (Figure 1c).^[21]^ As the photochemical analogue of the Hückel rule, he postulated that ground state aromatic systems are antiaromatic in the excited state and vice versa for antiaromatic ground state annulenes. His assumption for the lowest ππ* triplet state was extended to the lowest excited singlet state by Karadakov in 2008.^[22]^

## Results and Discussion

In 1985, Wan and Krogh reported the photolysis of fluoren‐9‐ol, which was “believed to be the formation of an aromatic 4π cationic system in the excited‐state”.^[23]^ Further photochemical studies revealed that the photochemical intermediate can be attacked by a nucleophilic solvent molecule for example water or methanol, resulting in 9‐hydroxy‐9*H*‐fluorene or 9‐methoxy‐9*H*‐fluorene.[^24^, ^25^, ^26^] This observation is supported by our work from 2018, where several conformationally locked fluorene‐based compounds were tested photochemically and showed the same behavior in water.^[27]^ As reviewed in 2014^[28]^ and recently presented by Ottosson, Bergman and coworkers for benzene,^[29]^ the principle of excited state aromaticity and antiaromaticity has potential for new photophysical and photochemical applications.

**Figure 2:**
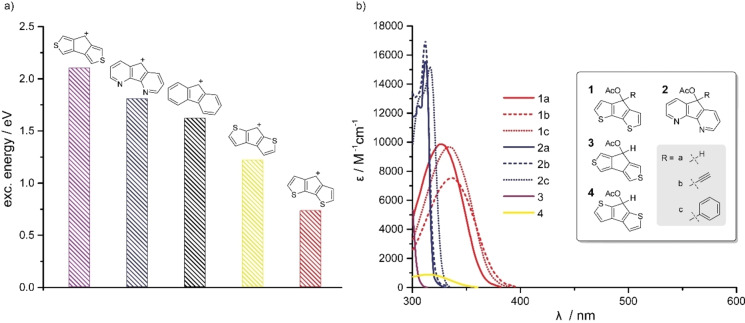
a) Calculated vertical excitation energies of cationic species in water. b) Measured absorption spectra and molar absorption coefficients of the various basic scaffolds and their derivatives with *meso*‐substitution.

For the second generation of fluorene‐based photocages, we took a step backward and thought about new scaffolds to exploit the excited‐state‐aromaticity concept for more efficient uncaging reactions. As reported, ground state antiaromatic compounds should in principle be able to cleave a specific bond during excitation to generate the aromatic system in the excited state. We initially sought to optimize several cyclic structures with computational methods and correlate it with experimental results. We focused therefore on heteroatomic substitutions and various ring sizes. As Winter and coworkers reported in 2014, the photo‐induced heterolysis in such compounds proceeds via a conical intersection between the lowest excited singlet state and the ground state.^[30]^ The presence of a productive conical intersection can be probed by computing the vertical absorption energy of the cationic species. The underlying mechanism is illustrated in Supporting Information Figure S8. Hence, a small vertical excitation energy of the cationic intermediate species can indicate a productive photoheterolysis, which allowed us to efficiently screen and rank different scaffolds. The ranking of fluorene‐like cationic species based on vertical excitation energies computed at the CAM‐B3LYP/def2‐TZVP level of theory is presented in Figure 2a. All optimized molecular geometries can be found in the Supporting Information.

We observed that the cationic intermediate of the fluorenol scaffold (black) has a relatively high vertical excitation energy of about 1.61 eV (Figure 2a). In comparison, the *para*‐sulfur‐cyclopenta‐dithiophene‐derivative (red) showed a significantly lower excitation energy of only 0.73 eV. The corresponding *meta‐* (violet) and *ortho*‐compounds (yellow) demonstrated also higher excitation energies. Also the aza‐compound (blue) showed a higher excitation energy than the fluorenol scaffold. Thus, the *para*‐sulfur‐cyclopentadithiophene‐derivative seemed to be the most promising candidate based on the computational results. Furthermore, NICS(0) calculations on the cationic species of Figure 2a corroborate the hypothesis that the cations are indeed antiaromatic in the electronic ground state (see Supporting Information Table S3).

With this assessment at hand, we started synthesizing the basic aza‐ and thio‐compounds. The *para‐*azo‐ and *para*‐thio‐*meso*‐carbonyl‐compounds were commercially available, the *meta* and *ortho*‐thio‐compounds had to be prepared from the corresponding thiophene compound according to literature.^[31]^ Starting from 3,4‐dibromothiophene, a copper(II) chloride‐assisted oxidative homo‐coupling was performed, followed by a bromine–lithium‐exchange and final cyclization with dimethylcarbamoyl chloride resulting in the *meta*‐compound (Scheme S1 in Supporting Information).

**Scheme 1:**
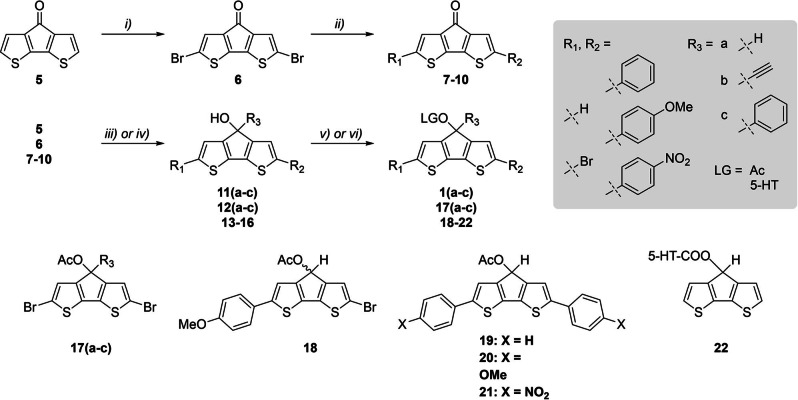
Synthesis of the *para*‐thio‐cyclopentadithiophene derivatives: i) NBS, THF, 0 °C, 30 min ; ii) arylboronic acid, Pd(PPh_3_)_4_, K_2_CO_3_, toluene/EtOH/H_2_O, 100 °C, 24–72 h ; iii) NaBH_4_, EtOH/MeOH, RT, 10–30 min ; iv) ethynyl/phenyl MgBr, abs. THF, 0 °C – RT, 2–24 h ; v) Ac_2_O, pyridine, room temperature, overnight ; vi) 1,1’‐carbonyldiimidazole, abs. DCM, mw, 45 °C, 60 min and after purification 5‐hydroxytryptamine (5‐HT) hydrochloride, abs. DMF, mw, 50 °C, 60 min. For further experimental details see Supporting Information (Supporting Information).

The *ortho*‐compound was similarly synthesized from 2,2’‐dibromo‐3,3’‐bithiophene, consecutive after the bromination of 3,3’‐bithiophene with *N*‐bromo‐succinimide. To finally obtain compounds **1 a**, **2 a**, **3** and **4**, all precursor compounds needed to be reduced with NaBH_4_ to the corresponding alcohol and finally provided with an acetyl LG for photochemical analysis (Scheme 1). For compounds **1 b**, **1 c**, **2 b** and **2 c**, Grignard reactions were carried out. For further experimental details, see the Supporting Information.

We could experimentally confirm that the *meta*‐compound **3** has the highest excitation energy and therefore the most hypsochromically‐shifted absorption maximum at 300 nm (Figure 2b). The *ortho*‐compound **4** and the aza‐compound **2 a** showed a slightly bathochromic absorption maximum at 314 nm and 312 nm, but in the case of compound **4** with a very low extinction coefficient. The *para*‐compound **1 a** has the most red shifted absorption maximum of all basic scaffolds and a good extinction coefficient.

All compounds exhibit low fluorescence, which is a further validation for the high uncaging quantum yields, as in many cases fluorescence is a competitive pathway for energy loss from the excited state.

Because the introduction of an electron donating substituent at the *meso*‐substitution turned out to be a successful strategy in previous photochemical studies,^[14]^ we substituted the *para*‐thio and aza‐compounds with ethynyl and phenyl‐groups. In the case of compounds **2 a**–**c** only the phenyl substitution afforded a slight shift of the absorption maximum of about 4 nm while the extinction coefficient remained essentially the same. Also for compounds **1 a**–**c** ethynyl and phenyl substitution resulted in a shift of the absorption maximum of 10 nm and 7 nm, respectively. Both modifications show only minor influence on the extinction coefficient.

We analyzed the photolysis behavior (Supporting Information Figure S1) and determined the uncaging quantum yields (Φ) at 365 nm (Table 1) and observed compounds **1 a** and **1 b** to possess high values of 28 % and 20 %. This is in agreement with the observation that these compounds display no significant fluorescence despite the limited conformational freedom for radiationless relaxation back to the ground state through internal conversion. Compound **1 c** displays also a high uncaging quantum yield of about 7.9 %. Compounds **3** and **4** do not show significant extinction above 300 nm. The aza‐compounds had no photoreactivity at 365 nm and showed only very little photolysis during irradiation at 310 nm.

**Table 1:** Photochemical properties of the various basic scaffolds and their derivatives with *meso*‐substitution.^[a]^

Compd.	*λ*_max_ [nm]	*ϵ*_λmax_ [M^−1^ cm^−1^]	*ϵ*_365_ [M^−1^ cm^−1^]	*Φ*_365_ [%]	*ϵΦ*_365_^[b]^ [M^−1^ cm^−1^]
**1 a 1 b 1 c 2 a 2 b 2 c 3 4**	326 336 333 312 312 316 300 314	9870 7540 9670 15530 16960 15190 5310 880	856 2763 2219 <1 <1 <1 <1 8	28 20 7.9 n.d. n.d. n.d. n.d. n.d.	240 553 175 n.d. n.d. n.d. n.d. n.d.

[a] All measurements were performed in a solvent mixture of 20 % 0.1 M PBS Buffer and 80 % MeOH. [b] Uncaging cross section (ϵΦ). n.d.=not determined.

In the next step, we focused on the *para*‐thio‐compound, as it offers the most promising photochemical properties compared to the other scaffolds. As for biological applications a red‐shifted absorption wavelength (>365 nm) with high extinction coefficients is desirable, we extended the π‐system of the chromophore, with various substituted phenyl‐compounds at the *α*‐position (Scheme 1).

The bromination was performed according to literature [31] with *N*‐bromo‐succinimide in abs. THF at 0 °C. The Suzuki couplings of the aromatic compounds were performed in a toluene/EtOH/H_2_O mixture under argon atmosphere. The reduction, Grignard reactions and acetylations were carried out similar to the procedure for the unmodified scaffolds.

The bromination at the *α*‐position resulted in a shift of the absorption spectra of about 22–23 nm for all three thio‐compounds (Table 2). Interestingly, at the same time it reduced the uncaging quantum yields drastically to 1.5 % for **17 a**, 3.1 % for **17 b** and to 7.9 % for **17 c**. This might be a hint that the uncaging happens in the singlet excited state, as halogen substituents are known to increase the intersystem crossing rate into triplet states. This appears to have a negative influence on the uncaging quantum yields in this case.^[6]^ As expected, all phenylic *α*‐substitutions resulted in a longer absorption wavelength and higher extinction coefficients than the unmodified and the brominated compounds.

**Table 2:** Photochemical properties of the bathochromically shifted compounds.

Computational data					Experimental data					
Compd.	R_1_, R_2_, R_3_	Excitation energy (S_1_) [eV]	λ (S_1_) [nm]	Oscillator strength^[b]^	*λ*_max_ [nm]	*ϵ*_λmax_ [M^−1^ cm^−1^]	*ϵ*_365_ [M^−1^ cm^−1^]	*Φ*_365_ [%]	*ϵΦ*_365_^[c]^ [M^−1^ cm^−1^]	*ϵΦ*_405_^[c]^ [M^−1^ cm^−1^]
**1 a 17 a 17 b 17 c 18 19 20 21**	H, H, H Br, Br, H Br, Br, Ethynyl Br, Br, Ph Br, MeOPh, H Ph^[a]^, H MeOPh^[a]^, H NO_2_Ph^[a]^, H	3.99 3.77 3.69 3.69 3.46 3.31 3.25 2.99	310 329 336 336 358 374 382 415	0.33 0.52 0.46 0.44 0.92 1.15 1.28 1.68	326 349 358 355 378 394 403 455	9870 16329 11805 11867 29200 17759 18291 17769	856 11837 11082 10551 26568 11311 9078 2006	28 1.5 3.1 7.9 3.9 4.6 4.9 n.r.^[d]^	240 178 344 834 1036 520 445 n.r.^[d]^	n.d. n.d. n.d. n.d. 625 755 893 n.r.^[d]^

All measurements were performed in a solvent mixture of 20 % 0.1 M PBS Buffer and 80 % MeOH. [a] R_1_=R_2_ [b] All transitions are of π‐π* character. [c] Uncaging cross section. [d] Compound showed no photoreaction, even at 405 and 455 nm irradiation.

**Figure 3:**
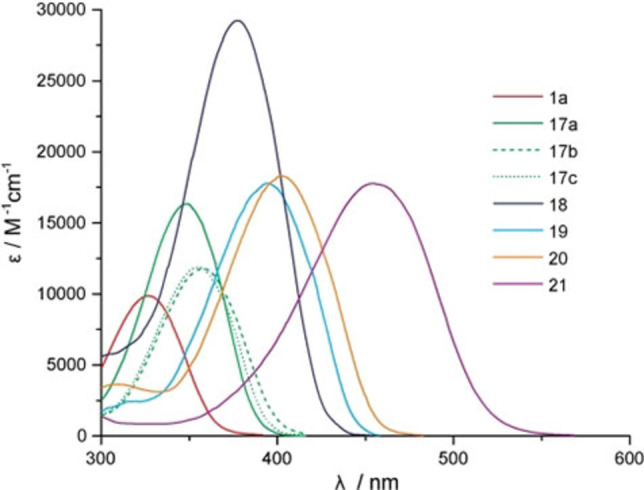
Absorption spectra and molar absorption coefficients of the various symmetrically and asymmetrically α‐substituted *para*‐thio compounds. Compound 1a is shown as comparison.

The asymmetrically substituted compound **18** demonstrates the highest ϵ (29200 M^−1^ cm^−1^) of all compounds and the symmetrically substituted compound **21** shows the most bathochromic absorption and a λ_max_ at 455 nm (Figure 3).

The uncaging quantum yields are reduced in comparison to the unmodified scaffolds, but still these compounds are more useful for biological applications, as they do not need UV irradiation for uncaging. This reduction can be explained by the additional conformational freedom of these compounds, which in general enables non‐radiative relaxation pathways (internal conversion) after excitation. However, compound **20** has still a high uncaging quantum yield of 4.9 % in its bathochromic shifted absorption range, compared to similar photocages in the literature above 400 nm.[^13^, ^14^, ^15^] With its quantum product (ϵ⋅Φ) of 893 M^−1^ cm^−1^ at 405 nm it is perfectly suited for biological applications. Compared to literature‐known photocages, with an ϵ⋅Φ of 200 M^−1^ cm^−1^, which could already be successfully used in biological applications,[^13^, ^14^, ^15^, ^16^, ^17^, ^18^] it shows more than a four‐fold higher efficiency. The most red‐shifted compound **21** showed no photoreaction during irradiation at various wavelengths. This may be explained by charge transfer to both nitro‐groups upon excitation and no sufficient cation stabilization.

**Figure 4:**
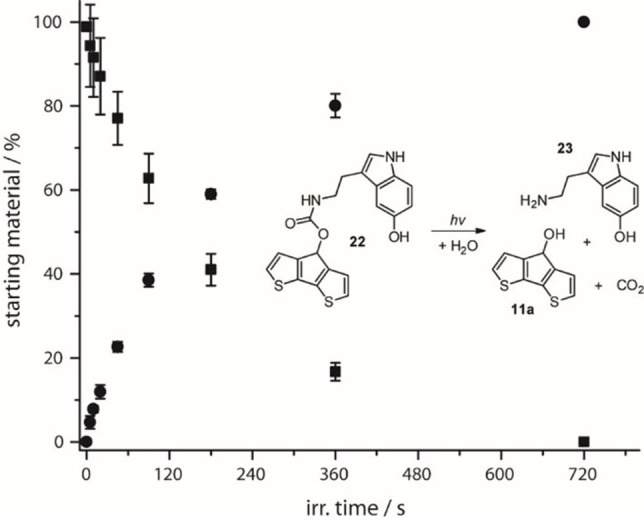
Photolysis for irradiation at 365 nm (11.2 mW) of compound **22** (squares, 37.6 nmol, OD_365_=0.26) and released serotonin **23** (5‐HT, circles) in 1 M PBS buffer with 36 % DMSO at 25 °C.

To demonstrate the effective release of biologically relevant leaving groups in buffer conditions with light, we chose the neurotransmitter serotonin (5‐hydroxytryptamine, 5‐HT, Figure 4).

It was synthetically attached via a carbamate‐linker in a two‐step, microwave‐assisted reaction, similar to compound **1 a**, which shows the best uncaging quantum yield. Upon irradiation at 365 nm or 405 nm, compound **22** shows fast photolysis with a quantum yield (Φ) of 4.9 %, which was tracked by HPLC (Figure 4). Already after 90 seconds, nearly 50 % and after 720 seconds the whole starting material was photolyzed.

**Figure 5:**
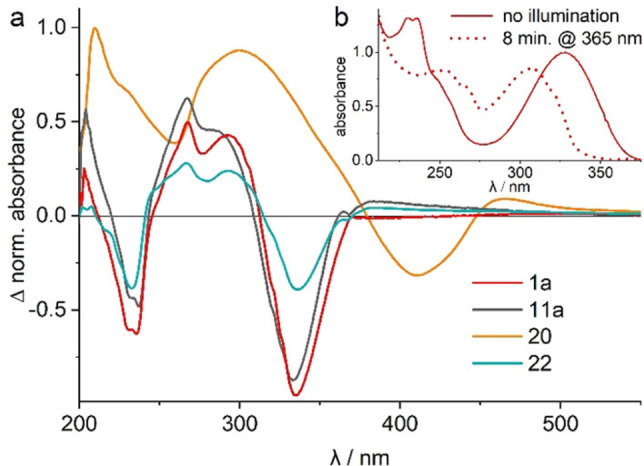
(a) Light‐induced difference absorption spectra of *para*‐thio compounds in MeOH before and after 8 minutes of illumination at 365 nm for **1 a**, **11 a** and **22** and at 420 nm for **20**. (b) Absorption spectra of **1 a** before and after 8 minutes of illumination at 365 nm (normalized to the main peak of the dark spectrum @ 327 nm).

Figure 5 shows the absorption changes of selected derivatives before and after illumination at the respective absorption maxima. Since both DFT calculations and uncaging quantum yields show the most promising results for para‐thio compounds, we have focused here on the unsubstituted compound with three different leaving groups (**1 a**, **11 a** and **22**) and the symmetrically substituted one with acetate as leaving group (**20**).

Similar to the initial absorption spectrum of compound **1 a** (Figure 5b), all three unsubstituted compounds possess two absorption bands at 327 nm and 233 nm, since they only differ in the leaving groups. Uncaging occurs upon illumination, which results in a hypsochromic shift of the absorption band at 327 nm to 309 nm and a decrease of the band at 233 nm. In addition, a new absorption band is formed at 266 nm, which is reflected in a positive contribution in the difference spectra (Figure 5a).

A comparison of derivatives with different leaving groups shows the acetate **1 a** to exhibit the largest absorption difference at 327 nm upon illumination. Since all three compounds were illuminated under the same conditions (wavelength, LED power and sample concentration), a qualitative statement can be made about the unknown quantum yield of **11 a**. This compound features a slightly smaller bleach of the main band at 327 nm than **1 a**. Accordingly, **11 a** with ^−^OH as leaving group should thus possess an uncaging quantum yield similar to the one of **1 a**, while the serotonin derivative **22** seems to have a lower Φ. The symmetrically substituted compound **20** with its bathochromically shifted absorption spectrum also undergoes a hypsochromic shift of the main absorption band at 405 nm by 22 nm during illumination (Supporting Information Figure S7). Unlike the other compounds, the two higher‐energy bands increase.

## Conclusion

We were able to optimize cyclic, heteroatom‐functionalized, low fluorescent compounds in terms of absorption wavelength, extinction coefficient, uncaging quantum yield and uncaging cross section. The identified trends are supported by quantum chemical investigations of the cationic intermediate structures, occurring during photolysis. It has been shown that an optimization of one parameter affects other parameters, as photochemical reactions pathways are complex and cannot be predicted easily. Upon shifting the absorption over 100 nm to higher wavelengths, the uncaging quantum yield decreases in all investigated systems. We present here compound **20**, which has a high uncaging quantum yield of 4.9 %, compared to similar photocages in the literature.[^13^, ^14^, ^15^, ^16^, ^17^, ^18^] With its uncaging cross section of 893 M^−1^ cm^−1^ at 405 nm it can be perfectly used for biological applications. Uncaging of the biologically relevant neurotransmitter serotonin has been demonstrated. Exploiting excited state aromaticity for the stabilization of intermediates is thus a useful and yet largely unexplored concept, which is here demonstrated to serve for further development of new photoactivatable compounds.

## Experimental Section

**Synthesis**: All reagents and solvents were purchased from commercial sources and were used as received. All reactions were performed in dry solvents and under argon atmosphere unless otherwise specified. For normal and reverse phase TLC pre‐coated ALUGRAM® Xtra SIL aluminum sheets from Macherey‐Nagel were used. Visualization was done with UV light (254 and 365 nm). NMR spectra were measured on a Bruker DPX 250, AV 300, AV 400, AV 500 MHz or DRX 600 device. Deuterated solvents (purchased at Eurisotop) were used for sample preparation. Spectra were referenced to the solvent peak. Mass spectrometry was performed on ThermoFisher Surveyor MSQ™ (ESI – Electrospray ionization) and MALDILTQ Orbitrap XL™ (HRMS ‐ High‐Resolution Mass Spectrometry) device from Thermo Fisher Scientific. Microwave reactions were performed in a Biotage® Initiator (Microwave power max. 400 W, Frequency 2450 MHz, 220–240 V, Power, max. 1100 VA). For further details see Supporting Information.

**Photochemical measurements**: UV‐vis spectra were measured in 1.0 cm quartz fluorescence cuvette (QS) from Hellma‐Analytics. Two different spectrometers were used. Ocean Optics USB4000 detector connected via optical fiber and convex lens, mounted in an adapter, to cuvette holder CVH100 (Thorlabs). In the opposed side of cuvette holder DH‐mini light source (Ocean Optics) was connected in the same way. The results were evaluated using an in‐house programmed software (PHITS; Photoswitch Irradiator Test Suite) based on LabVIEW. For more details see Reinfelds et al.^[32]^ This setup and software were also used for our chemical actinometry. The reference compound was an indolylfulgide photo‐switch. A solution of the fulgide (500–1000 μm) was irradiated with the respective light source (Thorlabs mounted LED, λ_max_=365 nm or 405 nm) in the same setup as for the irradiation experiments to convert the photoswitch from its 1Z form to 1 C or vice versa. The conversion was tracked via absorbtion and fitted with the respective quantum yields to get the photonflux. Afterwards, the caged compound of interest could be irradiated with known photon flux.^[32]^ Steady‐state fluorescence emission was recorded using a Hitachi F‐4500 spectrophotometer. The optical density (OD) was lower than or equal to 0.1 for fluorescence spectra. Light induced UV‐Vis measurements were performed with Thorlabs LEDs (λ_max_=365 nm and 420 nm) using Specord spectrometer S600 (Analytik Jena). Second spectrometer was JASCO−V650. In both cases spectra were measured with 1 nm steps.

**Computational methods**: All computations were performed using Q‐Chem 5.3.^[33]^ Cationic species were optimized at the CAM‐B3LYP/def2‐TZVP level of theory[^34^, ^35^] employing a polarizable continuum model ($\epsilon =78.4,\;n^{2}=1.76$ ).[^36^, ^37^] Afterwards, the five energetically lowest singlet excited states were computed with time‐dependent DFT (TD‐DFT) at the same level of theory. To support the hypothesis that the cationic species are antiaromatic in the electronic ground state, we carried out NICS(0) calculations^[38]^ at the CAM‐B3LYP/def2‐SVP level of theory. The NICS(0) values for each ring were obtained by placing a hydrogen ghost atom in the center of the respective ring and a subsequent calculation of chemical shielding tensors. The results are shown in Table S3. As all NICS(0) values are positive, one can expect that the cationic ground state species actually possess antiaromatic character, as predicted using Hückel's rule. The excited state calculations presented in Table 2 were run at the CAM‐B3LYP/def2‐SVP level of theory using a polarizable continuum model ($\epsilon =32.63,\;n^{2}=1.758$ ). Again, five singlet excited states were obtained with TD‐DFT after initial geometry optimization.

## Conflict of interest

The authors declare no conflict of interest.

## Supporting information

As a service to our authors and readers, this journal provides supporting information supplied by the authors. Such materials are peer reviewed and may be re‐organized for online delivery, but are not copy‐edited or typeset. Technical support issues arising from supporting information (other than missing files) should be addressed to the authors.

Supporting Information
