# Supplementary material for: Rethinking Uncaging: A New Antiaromatic Photocage Driven by a Gain of Resonance Energy
Source: Chemistry. 2021 Sep 2;27(56):14121–7. doi: 10.1002/chem.202102351 (PMC8519059; doi:10.1002/chem.202102351)
Supplement: Supplementary file 1 — Supporting Information [file CHEM-27-14121-s001.pdf]

Chemistry–A European Journal

Supporting Information

Rethinking Uncaging: A New Antiaromatic Photocage Driven by a Gain of Resonance Energy

Volker Hermanns, Maximilian Scheurer, Nils Frederik Kersten, Chahinez Abdellaoui, Josef Wachtveitl,* Andreas Dreuw,* and Alexander Heckel*

Table of contents

1. General information 2

2.1 Synthesis – Overview 4

2.2 Synthesis – General procedures 5

2.3 Synthesis – Experimental details 6

3. Photochemical Measurements 18

4. Theoretical calculations 26

5. NMR spectra of key compounds 27

6. Mass spectra of key compounds 57

7. References 64

1. General information

All reagents and solvents were purchased from TCI, Acros Organics (Thermofisher), Sigma Aldrich (Merck) and Fluorochem and were used as received. All reactions were performed in dry solvents and under argon atmosphere unless otherwise specified.

For normal and reverse phase TLC pre-coated ALUGRAM® Xtra SIL aluminum sheets from Macherey-Nagel were used. Visualization was done with UV light (254 and 365 nm). Technical grade solvents were used (EtOAc - ethyl acetate, Cyclohexane, DCM – dichloromethane).

NMR spectra were measured on a Bruker DPX 250, AV 300, AV 400, AV 500 MHz or DRX 600 device. Deuterated solvents (purchased at Eurisotop) were used for sample preparation. Spectra were referenced to the solvent peak. The values used therefore were: $CDCl_3$ ^1H 7.26, ^{13}C 77.16; DMSO-d_6 ^1H 2.50, ^{13}C 39.52; MeCN-d_3 ^1H 1.94, ^{13}C 1.32 and 118.2; CD_3OD-d_4 ^1H 3.31, ^{13}C 49.00; D_2O ^1H 4.79, ^{13}C no reference was done. In those cases when solvent mixtures were used (MeCN-d3 + D2O and CD3OD-d4 + D2O), spectra were calibrated to the organic solvent signal. Chemical shifts (δ) are reported on a ppm scale. Following abbreviations (or combinations thereof) were used to describe multiplicities: s = singlet, d = doublet, t = triplet, q = quartet, m = multiplet, br = broad. Coupling constants (J) are reported in Hertz (Hz).

Mass spectrometry was performed on ThermoFisher Surveyor MSQ™ (ESI - Electrospray ionization) and MALDILTQ Orbitrap XL™ (HRMS - High-Resolution Mass Spectrometry) device from Thermo Fisher Scientific.

Microwave reactions were performed in a Biotage® Initiator (Microwave power max. 400 W, Frequency 2450 MHz, 220-240 V, Power, max. 1100 VA).

UV-vis spectra were measured in 1.0 cm quartz fluorescence cuvette (QS) from Hellma-Analytics. Two different spectrometers were used. Ocean Optics USB4000 detector connected via optical fiber and convex lens, mounted in an adapter, to cuvette holder CVH100 (Thorlabs). In the opposed side of cuvette holder DH-mini light source (Ocean Optics) was connected in the same way. The results were evaluated using an in-house programmed software (PHITS; Photoswitch Irradiator Test Suite) based on LabVIEW. For more details see Reinfelds *et al.*.[1]

This setup and software were also used for our chemical actinometry. Reference compound was an indolylfulgide photo-switch. A solution of the fulgide (500–1000 µm) was irradiated with the respective light source (Thorlabs mounted LED, λ_{max}= 365 nm or 405 nm) in the same setup as for the irradiation experiments to convert the photoswitch from its 1Z form to 1C or vice versa. The conversion was tracked via absorbtion and fitted with the respective quantum yields to get the photonflux. Afterwards, the caged compound of interest could be irradiated with known photon flux.[1] Steady-state fluorescence emission was recorded using a Hitachi F-4500 spectrophotometer. The optical density (OD) was set lower than or equal to 0.1 for fluorescence spectra. Light induced UV-Vis measurements were performed with Thorlabs LEDs (λ_{max}= 365 nm and 420 nm) using Specord spectrometer S600 (Analytik Jena). Second spectrometer was JASCO-V650. In both cases spectra were measured with 1 nm steps.

Irradiation experiments were done in 1.0 cm quartz fluorescence cuvette (QS) from Hellma-Analytics equipped with a magnetic stirrer or for small volumes in 0.3 cm quartz fluorescence cuvette (QS) from Hellma-Analytics. Light sources (365 nm LED M365L2, 405 nm LED M405L4, 455 nm LED M455L4) were operated by DC2100 LED driver in external trigger mode (both from Thorlabs). As external trigger was used an in-house programmed software PHITS.

For high-performance liquid chromatography Agilent Technologies 1260 Infinity instrument was used, equipped with quaternary pump, automatic liquid sampler, thermostatted column compartment and diode array detector. Separation was done using MultoKrom® 100-5 C18 column (250 x 4.6 mm) from CS-Chromatographie Service GmbH. Binary solvent mixtures were used for elution. Typical gradients are described in Table S1 and shown in Figure S3.

2.1 Synthesis – Overview

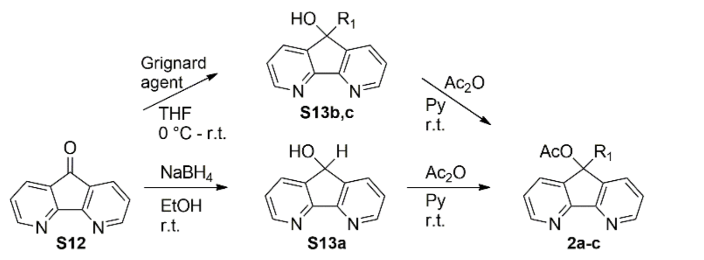

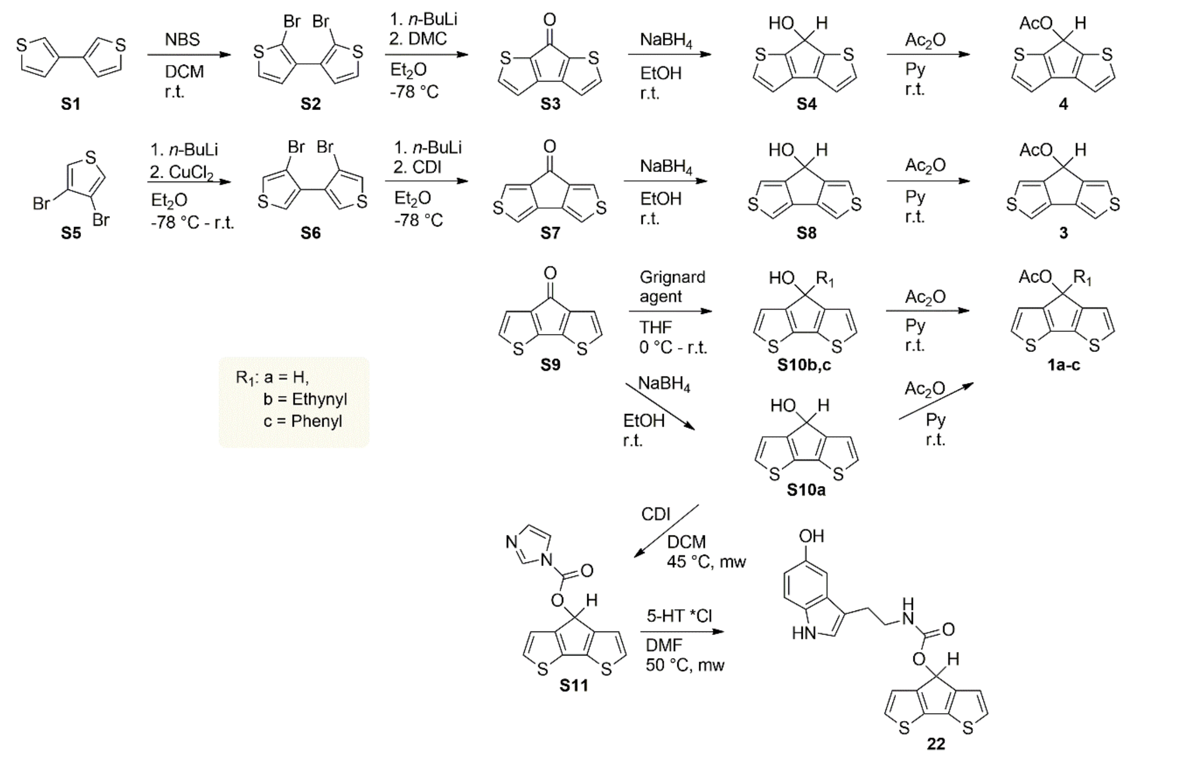

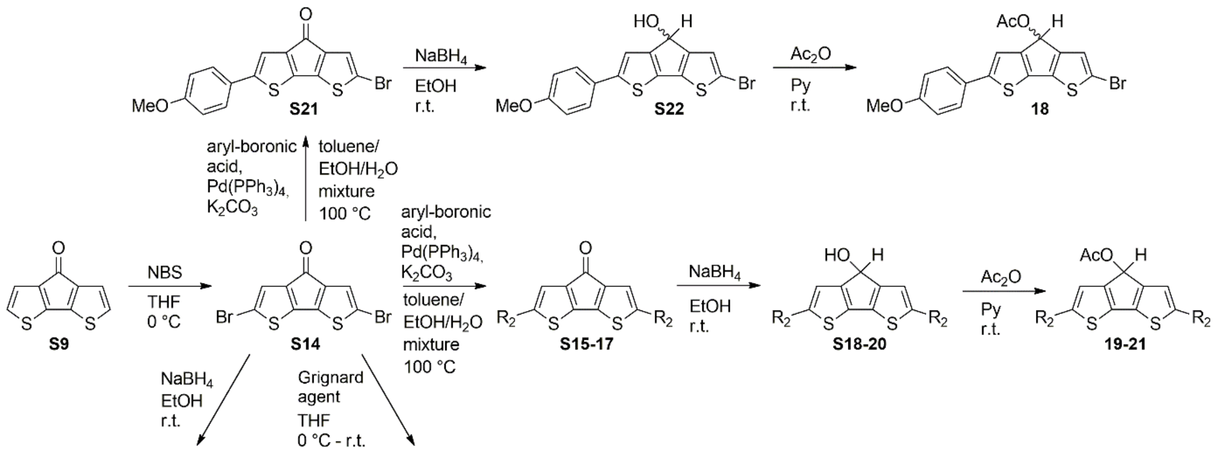

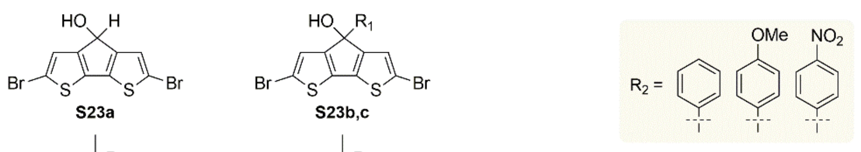

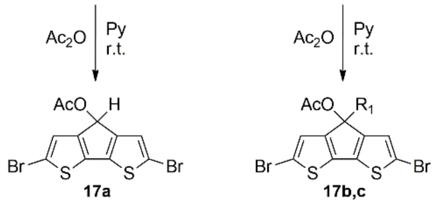

Scheme S1: Synthesis of the several photoactivatable compounds.

2.2 Synthesis – General procedures

General procedure A – reduction

The respective carbonyl compound (1 eq) was dissolved in 3-5 mL ethanol under argon atmosphere and ambient temperature. Afterwards sodium borohydride (2-3 eq) was added in small portions and the reaction mixtures was stirred at room temperature until the starting material was completely consumed (5 – 60 minutes). After dilution with water, the mixture was extracted three to five times with DCM. The combined organic phase was washed with water and brine and dried over Na_2SO_4. The solvent was removed under reduced pressure. In most cases no further purification was necessary.

General procedure B – Grignard-reaction

A solution of the individual carbonyl compound dissolved in 3-6 mL abs. THF under argon atmosphere was cooled down to 0 °C. Then the respective Grignard solution (0.5 M ethynylmagnesium bromide or 1 M phenylmagnesium bromide, 1.0 – 2.0 eq) was added dropwise. After full addition, the reaction mixture was allowed to warm up to room temperature and was stirred additional 3-5 hours. After that water was added and the mixture was extracted with DCM three to five times. The combined organic phases was dried over Na_2SO_4 and the solvent was removed under reduced pressure. The residue was purified via column chromatography (DCM:cyclohexane 2:1 to DCM).

General procedure C – acetylation

Under argon atmosphere the respective alcohol (1 eq) was dissolved in 2-6 mL pyridine and acetic acid anhydride (8-30 eq) was added. After stirring under light exclusion for 6-24 hours, the reaction progress was checked via TLC. When the reaction was completed, saturated $NaHCO_3$ solution and water were added to the mixture. Extraction with DCM was done three to five times, the organic phase was dried over Na_2SO_4 and the solvent was removed under reduced pressure. With deactivated silica gel (1% Et_3N), the residue was purified isocratic via column chromatography (cyclohexane:EtOAc 100:5).

General procedure D – Suzuki-Cross-Coupling

2,6-Dibromo-4H-cyclopenta[1,2-b:5,4-b']dithiophene-4-one (1 eq), K_2CO_3 (8.1 eq), and $Pd(PPh_3)_4$ (0.07 eq) and the respective boronic acid (phenylboronic acid, 4-methoxy-phenylboronic acid, 4-nitro-phenylboronic acid) were suspended in a mixture of 4 mL toluene, 2 mL EtOH and 2 mL water under argon atmosphere. The mixture was heated to reflux for 24-72 hours until the starting material was consumed and diluted with water. Extraction was performed three to five times with DCM and the combined organic phases were dried over Na_2SO_4. Purification was done with column chromatography (DCM: cyclohexane 2:1 to DCM).

2.3 Synthesis – Experimental details

Synthesis of 2,2'-Dibromo-3,3'-Dithiophene (**S2**)

Synthesis was done as described in literature.[2] Starting from compound S1 (885 mg, 5.32 mmol, 1 eq), 1.305 g (84%) of product S2 was obtained as colorless solid.

Yield: 1.305 g (84%)

TLC (cyclohexane): R_f = 0.60

^{1}H-NMR (400 MHz, DMSO-d_6): δ = 7.70 (d, $^3J_{H-H}$ = 5.6 Hz, 2 H, Ar-H), 7.12 (d, $^3J_{H-H}$ = 5.6 Hz, 2 H, Ar-H) ppm.

^{1}H-NMR (250 MHz, $CDCl_3$): δ = 7.31 (d, $^3J_{H-H}$ = 5.6 Hz, 2 H, Ar-H), 7.08 (d, $^3J_{H-H}$ = 5.6 Hz, 2 H, Ar-H) ppm.

Synthesis of 7H-Cyclopenta[1,2-b:4,3-b']dithiophene-7-one (**S3**)

Synthesis was done similar as described in literature.[3] Starting compound S2 (843 mg, 2.6 mmol, 1 eq) was dissolved in diethylether and cooled down to -78 °C, *n*-butyllithium (2.19 mL, 2.5 M, 5.46 mmol, 2.1 eq) was added dropwise. After stirring for one hour at -78 °C, dimethylcarbamoylchlorid (0.26 mL, 2.9 mmol, 1.1 eq) was added slowly. The mixture was allowed to warm up to room temperature and was stirred overnight. Dilution with water and sat. ammonium chlorid solution and extraction with EtOAc were done. After washing the combined organic phases with water and sat. sodium chlorid solution and drying over Na_2SO_4, purification via column chromatography was performed (DCM/cyclohexane, 2:1) resulting in 0.195 g (39%) of product S3.

Yield: 0.195 g (39%)

TLC (DCM:cyclohexane 2:1): R_f = 0.57

^{1}H-NMR (250 MHz, DMSO-d_6): δ = 8.01 (d, $^3J_{H-H}$ = 4.6 Hz, 2 H, Ar-H), 7.16 (d, $^3J_{H-H}$ = 4.6 Hz, 2 H, Ar-H) ppm.

Synthesis of 7H-Cyclopenta[1,2-b:4,3-b']dithiophene-7-ol (**S4**)

Synthesis was done as described in general procedure A. Starting from compound S3 (50 mg, 0.26 mmol), 50 mg (99%) of product S4 was obtained as colorless solid.

Yield: 0.050 g (99%)

TLC (cyclohexane:EtOAc 9:1): R_f = 0.14

^{1}H-NMR (250 MHz, DMSO-d_6): δ = 7.56 (d, $^3J_{H-H}$ = 4.9 Hz, 2 H, Ar-H), 7.14 (d, $^3J_{H-H}$ = 4.9 Hz, 2 H, Ar-H), 6.14 (d, $^3J_{H-H}$ = 7.6 Hz, 1 H, O-H), 5.53 (d, $^3J_{H-H}$ = 7.6 Hz, 1 H, C-H) ppm.

Synthesis of 7H-Cyclopenta[1,2-b:4,3-b']dithiophene-7-yl-acetate (*ortho*-derivative, **4**)

Synthesis was done as described in general procedure C. Starting from compound S4 (50 mg, 0.257 mmol), 45 mg (75%) of product 4 was obtained.

Yield: 0.045 g (75%)

TLC (DCM:cyclohexane 2:1): R_f = 0.49

^{1}H-NMR (250 MHz, DMSO-d$_6$): δ = 7.66 (d, $^3J_{H-H}$ = 4.9 Hz, 2 H, Ar-H), 7.20 (d, $^3J_{H-H}$ = 4.9 Hz, 2 H, Ar-H), 6.22 (s, 1 H, C-H), 2.14 (s, 3 H, C-H) ppm.

^{1}H-NMR (500 MHz, $CDCl_3$): δ = 7.38 (d, $^3J_{H-H}$ = 4.9 Hz, 2 H, Ar-H), 7.05 (d, $^3J_{H-H}$ = 4.9 Hz, 2 H, Ar-H), 6.17 (s, 1 H, C-H), 2.18 (s, 3 H, C-H) ppm.

^{13}C{^{1}H}-NMR (125.8 MHz, $CDCl_3$): δ = 171.56, 144.50, 142.74, 130.89, 118.39, 71.14, 21.08 ppm.

MALDI-MS: *m/z* calcd. for $C_{11}H_8O_2S_2$ $[M\cdot]^+$ 235.99657, found 235.86930.

Synthesis of 4,4'-Dibromo-3,3'-bithiophene (**S6**)

Synthesis was done as described in literature.[4] Starting from compound S5 (3.9 g, 16.12 mmol, 1 eq), 2.07 g (79%) of product S6 was obtained as a beige solid.

Yield: 2.07 g (79%)

^{1}H-NMR (250 MHz, DMSO-d$_6$): δ = 7.81 (d, $^4J_{H-H}$ = 3.4 Hz, 2 H, Ar-H), 7.69 (d, $^4J_{H-H}$ = 3.4 Hz, 2 H, Ar-H) ppm.

Synthesis of 7H-Cyclopenta[1,2-c:3,4-c']dithiophene-7-one (**S7**)

Compound S6 (173 mg, 0.543 mmol, 1 eq) was solved in dry diethylether and cooled down to -78 °C. Now *n*-butyllithium (0.44 mL, 2.5 M, 1.1 mmol, 2.1 eq) was added dropwise and the mixture was stirred for three hours. After that time, carbonyldiimidazole (314 mg, 1.94 mmol, 3.6 eq) was added slowly at -78 °C and the mixture was stirred for 30 minutes and allowed to warm up to room temperature. Saturated ammonium chlorid solution was added and diluted with water. After extraction with ethyl acetate, purification was done via column chromatography (DCM/cyclohexane, 2:1).

Yield: 0.010 g (8%)

TLC (DCM:cyclohexane 4:1): R_f = 0.53

^{1}H-NMR (250 MHz, $CDCl_3$): δ = 7.77 (d, $^4J_{H-H}$ = 2.1 Hz, 2 H, Ar-H), 7.09 (d, $^4J_{H-H}$ = 2.1 Hz, 2 H, Ar-H) ppm.

Synthesis of 7H-Cyclopenta[1,2-c:3,4-c']dithiophene-7-ol (**S8**)

Synthesis was done as described in general procedure A. Starting from compound S7 (50 mg, 0.26 mmol), 50 mg (99%) of product S8 was obtained.

Yield: 0.050 g (99%)

TLC (cyclohexane:EtOAc 9:1): R_f = 0.09

^{1}H-NMR (250 MHz, DMSO-d$_6$): δ = 7.43-7.40 (m, 2 H, Ar-H), 7.40-7.37 (m, 2 H, Ar-H), 5.77 (d, $^3J_{H-H}$ = 7.0 Hz, 1 H, O-H), 5.40 (d, $^3J_{H-H}$ = 7.0 Hz, 1 H, C-H) ppm.

Synthesis of 7H-Cyclopenta[1,2-c:3,4-c']dithiophene-7-yl-acetate (*meta*-derivative, **3**)

Synthesis was done as described in general procedure C. Starting from compound S8 (60 mg, 0.309 mmol), 25 mg (34%) of 3 was obtained as a brown solid.

Yield: 0.025 g (34%)

TLC (cyclohexane: DCM 5:1): R_f = 0.18

^{1}H-NMR (500 MHz, $CDCl_3$): δ = 7.31 (dd, $^4J_{H-H}$ = 2.2 Hz, 1.0 Hz, 2 H, Ar-H), 7.13 (d, $^4J_{H-H}$ = 2.2 Hz, 2 H, Ar-H), 6.36 (s, 1 H, C-H), 2.13 (s, 3 H, C-H) ppm.

^{13}C{^{1}H}-NMR (125.8 MHz, $CDCl_3$): δ = 171.51, 150.05, 139.96, 122.00, 113.45, 67.67, 21.29 ppm.

MALDI-MS: *m/z* calcd. for $C_{11}H_8O_2S_2$ $[M\cdot]^+$ 235.9966 found 235.8632.

Synthesis of 4H-Cyclopenta[1,2-b:5,4-b']dithiophene-4-ol (*para*-derivative, **11a, S10a**)

Synthesis was done as described in general procedure A. Starting from compound S9 (100 mg, 0.521 mmol), 105 mg (98%) of 11a was obtained.

Yield: 0.105 g (98%)

TLC (cyclohexane: EtOAc 9:1): R_f = 0.15

^{1}H-NMR (250 MHz, DMSO-d_6): δ = 7.38 (d, $^3J_{H-H}$ = 4.9 Hz, 2 H, Ar-H), 7.14 (d, $^3J_{H-H}$ = 4.9 Hz, 2 H, Ar-H), 5.72 (d, $^3J_{H-H}$ = 7.4 Hz, 1 H, O-H), 5.22 (d, $^3J_{H-H}$ = 7.4 Hz, 1 H, C-H) ppm.

Synthesis of 4H-Cyclopenta[1,2-b:5,4-b']dithiophene-4-yl-acetate (*para*-derivative, **1a**)

Synthesis was done as described in general procedure C. Starting from compound S10a (90 mg, 0.45 mmol), 84 mg (77%) of product 1a was obtained as an orange solid.

Yield: 0.84 g (77%)

TLC (DCM): R_f = 0.63

^{1}H-NMR (250 MHz, DMSO-d_6): δ = 7.44 (d, $^3J_{H-H}$ = 4.9 Hz, 2 H, Ar-H), 7.10 (d, $^3J_{H-H}$ = 4.9 Hz, 2 H, Ar-H), 6.18 (s, 1H, C-H), 2.12 (s, 3H, C-H) ppm.

^{1}H-NMR (500 MHz, $CDCl_3$): δ = 7.15 (d, $^3J_{H-H}$ = 4.9 Hz, 2 H, Ar-H), 7.09 (d, $^3J_{H-H}$ = 4.9 Hz, 2 H, Ar-H), 6.18 (s, 1 H, C-H), 2.16 (s, 3 H, C-H) ppm.

^{13}C{^{1}H}-NMR (125.8 MHz, $CDCl_3$): δ = 171.47, 148.91, 139.98, 125.77, 123.84, 69.90, 21.16 ppm.

MALDI-HRMS: *m/z* calcd. for $C_{11}H_8O_2S_2$ $[M+H]^+$ 237.00385, found 237.00408 (Δm = 0.00023, error 1.0 ppm).

Synthesis of 4*H*-Cyclopenta[1,2-b:5,4-b']dithiophene-4-yl-1*H*-imidazole-1-carboxylate (**S11**)

Compound S10a (90 mg, 0.46 mmol, 1 eq) was dissolved in 3 mL dry DCM and 1'-Carbonyldiimidazole (CDI) (150 mg, 0.93 mmol, 2 eq) was added in a microwave vial under argon atmosphere. The vial was placed in a microwave reactor and stirred at 45 °C (100 W) for 60 minutes. The reaction mixture was

then diluted with water, extracted three times with DCM and the combined organic phases were dried over Na_2SO_4. The solvent was removed under reduced pressure. No further purification was done and the product S11 (133 mg, 0.44 mmol) was directly used for the next synthesis step.

Synthesis of 4*H*-Cyclopenta[1,2-b:5,4-b']dithiophene-4-yl-(2-(5-hydroxy-1*H*-indol-3-yl-)ethyl)carbamate (**22**)

Compound S11 (133 mg, 0.44 mmol, 1 eq) was dissolved in 4 mL dry DMF and triethylamine (223 mg, 2.2 mmol, 5 eq) and serotonin-hydrochlorid (5-HT*Cl) (187 mg, 0.88 mmol, 2 eq) were added in a microwave vial under argon atmosphere. The vial was placed in a microwave reactor and stirred at 50 °C (100 W) for 45 minutes. After dilution with water and extraction with DCM, the combined organic phases were dried over Na_2SO_4. Then the solvent was removed under reduced pressure and purification was performed via column chromatography (cyclohexane/EE, 2:1). Compound 22 (151 mg, 87%) was obtained as a brown solid.

Yield: 0.151 g (87%)

TLC (Cyclohexane: EE 1:1): R_f = 0.5

^{1}H-NMR (400 MHz, DMSO-d_6): δ = 10.48 (bs, 1 H, Ar-OH), 8.59 (s, 1 H, N-H), 7.48 (t, $^3J_{H-H}$ = 5.8 Hz, 1H, Ar-H), 7.43 (d, $^3J_{H-H}$ = 5.0 Hz, 2 H, Ar-H), 7.14 - 7.11 (m, 3 H, Ar-H), 7.05 (d, $^4J_{H-H}$ = 2.0 Hz 1 H, Ar-H), 6.85 (d, $^4J_{H-H}$ = 2.0 Hz, 1 H, Ar-H), 6.59 (dd, $^3J_{H-H}$ = 8.6 Hz, $^4J_{H-H}$ = 2.3 Hz, 1 H, Ar-H), 6.11 (s, 1 H, Ar-H), 3.31 (m, overlaid with H_2O signal, Alkyl-H), 2.78 (t, $^3J_{H-H}$ = 7.5 Hz, 2 H, Alkyl-H) ppm.

Synthesis of 4-Ethynyl-4H-cyclopenta[1,2-b:5,4-b']dithiophene-4-ol (**11b**, **S10b**)

Synthesis was done as described in general procedure B with ethylnylmagnesium bromide as grignard agent. Starting from compound S9 (100 mg, 0.52 mmol), 80 mg (70%) of product S10b was obtained.

Yield: 0.08 g (70%)

TLC (DCM): R_f = 0.51

^{1}H-NMR (400 MHz, DMSO-d_6): δ = 7.42 (d, $^3J_{H-H}$ = 4.9 Hz, 2 H, Ar-H), 7.14 (d, $^3J_{H-H}$ = 4.9 Hz, 2 H, Ar-H), 6.63 (s, 1H, O-H), 3.26 (s, 1H, C-H) ppm.

Synthesis of 4-Ethynyl-4H-cyclopenta[1,2-b:5,4-b']dithiophene-4-yl-acetate (**1b**)

Synthesis was done as described in general procedure C. Starting from compound S10b (86 mg, 0.39 mmol), 30 mg (31%) of product 1b was obtained as a red solid.

Yield: 0.03 g (31%)

TLC (cyclohexane: DCM 5:1): R_f = 0.75

^{1}H-NMR (250 MHz, DMSO-d_6): δ = 7.47 (d, $^3J_{H-H}$ = 5.0 Hz, 2 H, Ar-H), 7.23 (d, $^3J_{H-H}$ = 5.0 Hz, 2 H, Ar-H), 3.60 (s, 1H, C-H), 2.06 (s, 3H, C-H) ppm.

^{1}H-NMR (500 MHz, $CDCl_3$): δ = 7.28 (d, $^3J_{H-H}$ = 4.9 Hz, 2 H, Ar-H), 7.16 (d, $^3J_{H-H}$ = 4.9 Hz, 2 H, Ar-H), 2.47 (s, 1 H, C-H), 2.10 (s, 3 H, C-H) ppm.

$^{13}C\{^1H\}$-NMR (125.8 MHz, $CDCl_3$): δ = 169.78, 150.09, 139.03, 126.12, 124.22, 79.29, 71.91, 29.85, 21.55 ppm.

ESI-MS: *m/z* calcd. for $C_{13}H_8O_2S_2$ $[M+H]^+$ 261.00, found 261.09.

Synthesis of 4-Phenyl-4H-cyclopenta[1,2-b:5,4-b']dithiophene-4-ol (**11c**, **S10c**)

Synthesis was done as described in general procedure B with phenylmagnesium bromide as grignard agent. Starting from compound S9 (100 mg, 0.52 mmol), 135 mg (96%) of product S10c was obtained.

Yield: 0.135 g (96%)

TLC (DCM): R_f = 0.52

^1H-NMR (400 MHz, DMSO-d_6): δ = 7.37 (d, $^3J_{H-H}$ = 4.9 Hz, 2 H, Ar-H), 7.36-7.33 (m, 2 H, Ar-H), 7.29-7.19 (m, 3 H, Ar-H), 6.95 (d, $^3J_{H-H}$ = 4.9 Hz, 2 H, Ar-H), 6.38 (s, 1H, O-H) ppm.

Synthesis of 4-Phenyl-4H-cyclopenta[1,2-b:5,4-b']dithiophene-4-yl-acetate (**1c**)

Synthesis was done as described in general procedure C. Starting from compound S10c (120 mg, 0.44 mmol), 130 mg (94%) of product 1c was obtained.

Yield: 0.130 g (94%)

TLC (DCM: cyclohexane 2:1): R_f = 0.56

^1H-NMR (250 MHz, DMSO-d_6): δ = 7.44 (d, $^3J_{H-H}$ = 4.9 Hz, 2 H, Ar-H), 7.36-7.25 (m, 5 H, Ar-H), 7.13 (d, $^3J_{H-H}$ = 4.9 Hz, 2 H, Ar-H), 2.08 (s, 3 H, C-H) ppm.

^1H-NMR (500 MHz, $CDCl_3$): δ = 7.37-7.34 (m, 2 H, Ar-H), 7.32-7.27 (m, 3 H, Ar-H), 7.15-7.12 (m, 4 H, Ar-H), 2.11 (s, 3 H, C-H) ppm.

$^{13}C\{^1H\}$-NMR (125.8 MHz, $CDCl_3$): δ = 169.75, 152.66, 139.57, 138.64, 128.82, 128.08, 125.72, 124.94, 124.69, 84.05, 21.86 ppm.

MALDI-HRMS: *m/z* calcd. for $C_{17}H_{12}O_2S_2$ $[M\cdot]^+$ 312.02787, found 312.02769 (Δm = 0.00018, error 0.6 ppm).

Synthesis of 5H-Cyclopenta[1,2-b:5,4-b']dipyridine-5-ol (**S13a**)

Synthesis was done as described in general procedure A. Starting from compound S12 (10 mg, 0.55 mmol), 8 mg (74%) of product S13a was obtained as yellow solid.

Yield: 0.08 g (74%)

TLC (EtOAc:cyclohexane 10:1): R_f = 0.11

^1H-NMR (250 MHz, DMSO-d_6): δ = 8.65 (dd, $^3J_{H-H}$ = 5.05 Hz, 2H, Ar-H), 8.04 (dd, $^3J_{H-H}$ = 7.62 Hz, 2H, Ar-H), 7.42 (q, $^3J_{H-H}$ = 12.52 Hz, 2H, Ar-H), 6.08 (d, $^3J_{H-H}$ = 7.63 Hz, 1H OH), 5.59 (d, $^3J_{H-H}$ = 7.88 Hz, 1H, Ar-H) ppm.

Synthesis of 5H-Cyclopenta[1,2-b:5,4-b']dipyridine-5-yl-acetate (**2a**)

Synthesis was done as described in general procedure C. Starting from compound S13a (5 mg, 0.2 mmol), 3 mg (51%) of product 2a was obtained as colorless solid.

Yield: 0.003 g (51%)

TLC (EtOAc:cyclohexane 10:1): R_f = 0.28

^{1}H-NMR (400 MHz, D_2O): δ = 8.53 (dd, $^3J_{H-H}$ = 4.77 Hz, 2H, H1, H1‘), 7.91 (dd, $^3J_{H-H}$ = 7.43 Hz, 2H, H3, H3‘), 7.37 (dd, $^3J_{H-H}$ = 12.64 Hz, 2H, H2, H2‘), 2.19 (s, 3H, H5) ppm.

^{13}C{^{1}H}-NMR (125.8 MHz, DMSO-d$_6$): δ = 171.1, 158.1, 151.1, 137.5, 134.0, 123.8, 70.5, 20.7 ppm.

MALDI-HRMS: *m/z* calcd. for $C_{13}H_{10}N_2O_2$ $[M+H]^+$ 227.082053, found 227.08193 (Δm = 0.000123, error 0.5 ppm).

Synthesis of 5-Ethinyl-5H-cyclopenta[1,2-b:5,4-b']dipyridine-5-ol (**S13b**)

Synthesis was done as described in general procedure B with ethylnylmagnesium bromide as grignard agent. Starting from compound S12 (500 mg, 2.74 mmol), 190 mg (33%) of product S13b was obtained.

Yield: 0.19 g (33%)

TLC (EtOAc): R_f = 0.32

^{1}H-NMR (250 MHz, DMSO-d$_6$): δ = 8.69 (dd, $^3J_{H-H}$ = 4.9 Hz, $^4J_{H-H}$ = 1.5 Hz, 2 H, Ar-H), 8.10 (dd, $^3J_{H-H}$ = 7.7 Hz, $^4J_{H-H}$ = 1.5 Hz, 2 H, Ar-H), 7.48 (dd, $^3J_{H-H}$ = 4.9 Hz, $^3J_{H-H}$ = 7.7 Hz, 2 H, Ar-H), 6.98 (s, 1 H, O-H), 3.52 (s, 1 H, C-H) ppm.

Synthesis of 5-Ethinyl-5H-cyclopenta[1,2-b:5,4-b']dipyridine-5-yl-acetate (**2b**)

Synthesis was done as described in general procedure C. Starting from compound S13b (70 mg, 0.34 mmol), 56 mg (67%) of product 2b was obtained.

Yield: 0.056 g (67%)

TLC (DCM: MeOH 50:1): R_f = 0.06

^{1}H-NMR (250 MHz, DMSO-d$_6$): δ = 8.74 (dd, $^3J_{H-H}$ = 4.9 Hz, $^4J_{H-H}$ = 1.5 Hz, 2 H, Ar-H), 8.21 (dd, $^3J_{H-H}$ = 7.8 Hz, $^4J_{H-H}$ = 1.5 Hz, 2 H, Ar-H), 7.51 (dd, $^3J_{H-H}$ = 4.9 Hz, $^3J_{H-H}$ = 7.8 Hz, 2 H, Ar-H), 3.91 (s, 1 H, C-H), 2.04 (s, 3 H, C-H) ppm.

^{1}H-NMR (500 MHz, $CDCl_3$): δ = 8.75 (dd, $^3J_{H-H}$ = 4.9 Hz, $^4J_{H-H}$ = 1.5 Hz, 2 H, Ar-H), 8.20 (dd, $^3J_{H-H}$ = 7.8 Hz, $^4J_{H-H}$ = 1.5 Hz, 2 H, Ar-H), 7.34 (dd, $^3J_{H-H}$ = 4.9 Hz, $^3J_{H-H}$ = 7.8 Hz, 2 H, Ar-H), 2.63 (s, 1 H, C-H), 2.08 (s, 3 H, C-H) ppm.

^{13}C{^{1}H}-NMR (125.8 MHz, $CDCl_3$): δ = 169.72, 157.80, 152.32, 138.98, 134.26, 124.23, 78.86, 74.84, 74.71, 21.59 ppm.

MALDI-HRMS: *m/z* calcd. for $C_{15}H_{10}N_2O_2$ $[M+H]^+$ 251.08150, found 251.08165 (Δm = 0.00015, error 0.6 ppm).

Synthesis of 5-Phenyl-5H-cyclopenta[1,2-b:5,4-b']dipyridine-5-ol (**S13c**)

Synthesis was done as described in general procedure B with phenylmagnesium bromide as grignard agent. Starting from compound S12 (500 mg, 2.74 mmol), 291 mg (41%) of product S13c was obtained.

Yield: 0.291 g (41%)

TLC (EtOAc): R_f = 0.31

^{1}H-NMR (250 MHz, DMSO-d$_6$): δ = 8.65 (dd, $^3J_{H-H}$ = 4.9 Hz, $^4J_{H-H}$ = 1.5 Hz, 2 H, Ar-H), 7.73 (dd, $^3J_{H-H}$ = 7.7 Hz, $^4J_{H-H}$ = 1.5 Hz, 2 H, Ar-H), 7.36 (dd, $^3J_{H-H}$ = 4.9 Hz, $^3J_{H-H}$ = 7.7 Hz, 2 H, Ar-H), 7.31-7.22 (m, 5 H, Ar-H), 6.67 (s, 1 H, O-H) ppm.

Synthesis of 5-Phenyl-5H-cyclopenta[1,2-b:5,4-b']dipyridine-5-yl-acetate (**2c**)

Synthesis was done as described in general procedure C. Starting from compound S13c (100 mg, 0.38 mmol), 87 mg (75%) of product 2c was obtained as a colorless solid.

Yield: 0.087 g (75%)

TLC (EtOAc): R_f = 0.48

^{1}H-NMR (250 MHz, DMSO-d$_6$): δ = 8.68 (dd, $^3J_{H-H}$ = 4.9 Hz, $^4J_{H-H}$ = 1.5 Hz, 2 H, Ar-H), 7.90 (dd, $^3J_{H-H}$ = 7.7 Hz, $^4J_{H-H}$ = 1.5 Hz, 2 H, Ar-H), 7.40 (dd, $^3J_{H-H}$ = 4.9 Hz, $^3J_{H-H}$ = 7.7 Hz, 2 H, Ar-H), 7.37-7.23 (m, 5 H, Ar-H), 2.11 (s, 3 H, C-H) ppm.

^{1}H-NMR (500 MHz, CDCl$_3$): δ = 8.72 (dd, $^3J_{H-H}$ = 4.9 Hz, $^4J_{H-H}$ = 1.5 Hz, 2 H, Ar-H), 7.79 (dd, $^3J_{H-H}$ = 7.7 Hz, $^4J_{H-H}$ = 1.5 Hz, 2 H, Ar-H), 7.31-7.28 (m, 3 H, Ar-H), 7.26-7.23 (m, 4 H, Ar-H), 2.13 (s, 3 H, C-H) ppm.

^{13}C{^{1}H}-NMR (125.8 MHz, CDCl$_3$): δ = 169.16, 158.43, 151.52, 141.62, 139.09, 133.18, 128.98, 128.45, 124.89, 123.90, 85.33, 21.79 ppm.

MALDI-HRMS: *m/z* calcd. for $C_{19}H_{14}N_2O_2$ $[M+H]^+$ 303.11280, found 303.11282 (Δm = 0.00002, error 0.1 ppm).

Synthesis of 2,6-Dibromo-4H-cyclopenta[1,2-b:5,4-b']dithiophene-4-one (**6**, **S14**)

Synthesis was performed similar to literature.[5] Compound S9 (100 mg, 0.52 mmol, 1 eq) was dissolved in dry tetrahydrofuran under argon atmosphere and cooled down to 0 °C. Then *N*-bromosuccinimide was added in small portions over two minutes. After stirring for half an hour the mixture was diluted with water and extracted three times with dichloromethane. Purification was performed via column chromatography (CH 100 -> CH/EtOAc, 100:6). 6 (184 mg, 98%) could be obtained as dark violet crystals.

Yield: 2.02 g (98%)

TLC (DCM:cyclohexane 1:1): R_f = 0.56

^{1}H-NMR (250 MHz, DMSO-d$_6$): δ = 7.25 (s, 2 H, Ar-H) ppm.

Synthesis of 2,6-Dibromo-4H-cyclopenta[1,2-b:5,4-b']dithiophene-4-ol (**12a**, **S23a**)

Synthesis was done as described in general procedure A. Starting from compound S14 (100 mg, 0.29 mmol), 100 mg (98%) of product 12a was obtained.

Yield: 0.1 g (98%)

TLC (cyclohexane:EtOAc 9:1): R_f = 0.19

^{1}H-NMR (250 MHz, DMSO-d_6): δ = 7.27 (s, 2 H, Ar-H), 5.85 (d, $^3J_{H\text{-}H}$ = 7.6 Hz, 1 H, O-H), 5.21 (d, $^3J_{H\text{-}H}$ = 7.6 Hz, 1 H, C-H) ppm.

Synthesis of 2,6-Dibromo-4H-cyclopenta[1,2-b:5,4-b']dithiophene-4-yl-acetate (**17a**)

Synthesis was done as described in general procedure C. Starting from compound S23a (46 mg, 0.13 mmol), 30 mg (58%) of product 17a was obtained as colorless solid.

Yield: 0.03 g (58%)

TLC (cyclohexane:EtOAc 20:1): R_f = 0.39

^{1}H-NMR (250 MHz, DMSO-d_6): δ = 7.22 (s, 2 H, Ar-H), 6.17 (s, 1 H, C-H), 2.12 (s, 3 H, C-H) ppm.

^{1}H-NMR (500 MHz, $CDCl_3$): δ = 7.10 (s, 2 H, Ar-H), 6.09 (s, 1 H, C-H), 2.15 (s, 3 H, C-H) ppm.

^{13}C{^{1}H}-NMR (125.8 MHz, $CDCl_3$): δ = 171.16, 146.76, 139.71, 126.77, 112.42, 70.26, 21.01 ppm.

MALDI-MS: *m/z* calcd. for $C_{11}H_6Br_2O_2S_2$ $[M\cdot]^+$ 391.82, found 392.04.

Synthesis of 2,6-Dibromo-4-ethinyl-4H-cyclopenta[1,2-b:5,4-b']dithiophene-4-ol (**12b**, **S23b**)

Synthesis was done as described in general procedure B with ethylnylmagnesium bromide as grignard agent. Starting from compound S14 (100 mg, 0.29 mmol), 79 mg (68%) of product 12b was obtained.

Yield: 0.073 g (68%)

TLC (DCM): R_f = 0.5

^{1}H-NMR (250 MHz, DMSO-d_6): δ = 7.29 (s, 2 H, Ar-H), 6.78 (s, 1 H, C-H), 3.38 (s, 1 H, C-H) ppm.

Synthesis of 2,6-Dibromo-4-ethinyl-4H-cyclopenta[1,2-b:5,4-b']dithiophene-4-yl-acetate (**17b**)

Synthesis was done as described in general procedure C. Starting from compound S23b (60 mg, 0.16 mmol), 65 mg (97%) of product 17b was obtained as off-white solid.

Yield: 0.065 g (97%)

TLC (DCM): R_f = 0.86

^{1}H-NMR (500 MHz, $CDCl_3$): δ = 7.30 (s, 2 H, Ar-H), 2.49 (s, 1 H, C-H), 2.10 (s, 3 H, C-H) ppm.

^{13}C{^{1}H}-NMR (125.8 MHz, $CDCl_3$): δ = 169.54, 147.92, 138.72, 127.09, 112.90, 78.11, 72.81, 72.01, 21.40 ppm.

MALDI-MS: *m/z* calcd. for $C_{13}H_6Br_2O_2S_2$ [M·]$^+$ 415.82, found 416.16.

Synthesis of 2,6-Dibromo-4-phenyl-4H-cyclopenta[1,2-b:5,4-b']dithiophene-4-ol (**12c**, **S23c**)

Synthesis was done as described in general procedure B with phenylmagnesium bromide as grignard agent. Starting from compound S14 (100 mg, 0.29 mmol), 109 mg (89%) of 12c was obtained as yellow crystals.

Yield: 0.109 g (89%)

TLC (DCM): R_f = 0.73

^{1}H-NMR (250 MHz, DMSO-d$_6$): δ = 7.38-7.23 (m, 5 H, Ar-H), 7.10 (s, 2 H, Ar-H), 6.54 (s, 1 H, C-H) ppm.

Synthesis of 2,6-Dibromo-4-phenyl-4H-cyclopenta[1,2-b:5,4-b']dithiophene-4-yl-acetate (**17c**)

Synthesis was done as described in general procedure C. Starting from compound S23c (100 mg, 0.23 mmol), 105 mg (95%) of 17c was obtained.

Yield: 0.105 g (95%)

TLC (DCM): R_f = 0.93

^{1}H-NMR (500 MHz, $CDCl_3$): δ = 7.34-7.27 (m, 5 H, Ar-H), 7.14 (s, 2 H, Ar-H), 2.12 (s, 3 H, C-H) ppm.

^{13}C{^{1}H}-NMR (125.8 MHz, $CDCl_3$): δ = 169.52, 150.60, 138.11, 129.05, 128.51, 127.43, 124.73, 112.51, 84.49, 29.85, 21.73 ppm.

MALDI-MS: *m/z* calcd. for $C_{17}H_{10}Br_2O_2S_2$ [M·]$^+$ 467.85, found 468.02.

Synthesis of 2,6-Diphenyl-4H-cyclopenta[1,2-b:5,4-b']dithiophene-4-one (**7**, **S15**)

Synthesis was done as described in general procedure D with phenylboronic acid. Starting from compound S14 (100 mg, 0.29 mmol), 75 mg (76%) of 7 was obtained.

Yield: 0.075 g (76%)

TLC (cyclohexane:EtOAc 5:1): R_f = 0.53

^{1}H-NMR (250 MHz, $CDCl_3$): δ = 7.58-7.50 (m, 4 H, Ar-H), 7.45-7.36 (m, 4 H, Ar-H), 7.36-7.30 (m, 2 H, Ar-H), 7.23 (s, 2 H, Ar-H) ppm.

Synthesis of 2,6-Diphenyl-4H-cyclopenta[1,2-b:5,4-b']dithiophene-4-ol (**13**, **S18**)

Synthesis was done as described in general procedure A. Starting from compound S15 (35 mg, 0.1 mmol), 44 mg (90%) of 13 was obtained.

Yield: 0.044 g (90%)

TLC (cyclohexane:EtOAc 9:1): R_f = 0.07

^{1}H-NMR (250 MHz, DMSO-d$_6$): δ = 7.71-7.56 (m, 6 H, Ar-H), 7.47-7.38 (m, 4 H, Ar-H), 7.34-7.27 (m, 2 H, Ar-H), 5.89 (s, 1 H, O-H), 5.34 (s, 1 H, C-H) ppm.

Synthesis of 2,6-Diphenyl-4H-cyclopenta[1,2-b:5,4-b']dithiophene-4-yl-acetate (**19**)

Synthesis was done as described in general procedure C. Starting from compound S18 (35 mg, 0.1 mmol), 40 mg (90%) of product 19 was obtained as yellow solid.

Yield: 0.04 g (90%)

TLC (DCM:cyclohexane 1:1): $R_f = 0.43$

^{1}H-NMR (300 MHz, DMSO-d$_6$): δ = 7.67 (d, $^{3}J_{H-H}$ = 7.3 Hz, 4 H, Ar-H), 7.54 (s, 2 H, Ar-H), 7.43 (t, $^{3}J_{H-H}$ = 7.3 Hz, 4 H, Ar-H), 7.32 (t, $^{3}J_{H-H}$ = 7.3 Hz, 2 H, Ar-H), 6.34 (s, 1 H, C-H), 2.18 (s, 3 H, C-H) ppm.

^{1}H-NMR (500 MHz, $CDCl_3$): δ = 7.59 (d, $^{3}J_{H-H}$ = 7.3 Hz, 4 H, Ar-H), 7.38 (t, $^{3}J_{H-H}$ = 7.3 Hz, 4 H, Ar-H), 7.34 (s, 2 H, Ar-H), 7.28 (t, $^{3}J_{H-H}$ = 7.3 Hz, 2 H, Ar-H), 6.25 (s, 1 H, C-H), 2.21 (s, 3 H, C-H) ppm.

^{13}C{^{1}H}-NMR (125.8 MHz, $CDCl_3$): δ = 171.52, 149.10, 145.83, 139.24, 134.74, 129.16, 127.68, 125.47, 119.89, 70.54, 21.20 ppm.

MALDI-HRMS: *m/z* calcd. for $C_{23}H_{16}O_2S_2$ $[M\cdot]^+$ 388.05917, found 388.05834 (Δm = 0.00083, error 2.1 ppm).

Synthesis of 2,6-Bis(4-methoxyphenyl)-4H-cyclopenta[1,2-b:5,4-b']dithiophene-4-one (**9**, **S16**)

Synthesis was done as described in general procedure D with 4-methoxyboronic acid. Starting from compound S14 (100 mg, 0.29 mmol), 170 mg of product 9 with small impurities was obtained.

Yield: 0.17 g (with small impurities)

^{1}H-NMR (250 MHz, DMSO-d$_6$): δ = 7.46 (d, $^{3}J_{H-H}$ = 7.4 Hz, 4 H, Ar-H), 7.09 (s, 2 H, Ar-H), 6.92 (d, $^{3}J_{H-H}$ = 7.4 Hz, 4 H, Ar-H), 3.84 (s, 6 H, Ar-H) ppm.

Synthesis of 2,6-Bis(4-methoxyphenyl)-4H-cyclopenta[1,2-b:5,4-b']dithiophene-4-ol (**15**, **S19**)

Synthesis was done as described in general procedure A. Starting from compound S16 (150 mg, 0.37 mmol), 50 mg (33%) of 15 was obtained.

Yield: 0.05 g (33%)

TLC (DCM): $R_f = 0.24$

^{1}H-NMR (600 MHz, DMSO-d$_6$): δ = 7.59 (d, $^{3}J_{H-H}$ = 8.7 Hz, 4 H, Ar-H), 7.45 (s, 2 H, Ar-H), 6.99 (d, $^{3}J_{H-H}$ = 8.7 Hz, 4 H, Ar-H), 5.82 (d, $^{3}J_{H-H}$ = 8.0 Hz, 1 H, O-H), 5.29 (d, $^{3}J_{H-H}$ = 8.0 Hz, 1 H, C-H), 3.79 (s, 6 H, C-H) ppm.

Synthesis of 2,6-Bis(4-methoxyphenyl)-4H-cyclopenta[1,2-b:5,4-b']dithiophene-4-yl-acetate (**20**)

Synthesis was done as described in general procedure C. Starting from compound S19 (50 mg, 0.12 mmol), 40 mg (73%) of 20 was obtained as yellow solid.

Yield: 0.04 g (73%)

TLC (DCM:cyclohexane 2:1): R_f = 0.44

^{1}H-NMR (250 MHz, DMSO-d$_6$): δ = 7.58 (d, $^3J_{H-H}$ = 8.8 Hz, 4 H, Ar-H), 7.38 (s, 2 H, Ar-H), 6.99 (d, $^3J_{H-H}$ = 8.8 Hz, 4 H, Ar-H), 6.29 (s, 1 H, C-H), 3.79 (s, 6 H, C-H), 2.17 (s, 3 H, C-H) ppm.

^{1}H-NMR (500 MHz, $CDCl_3$): δ = 7.51 (d, $^3J_{H-H}$ = 8.8 Hz, 4 H, Ar-H), 7.21 (s, 2 H, Ar-H), 6.92 (d, $^3J_{H-H}$ = 8.8 Hz, 4 H, Ar-H), 6.22 (s, 1 H, C-H), 3.84 (s, 6 H, C-H), 2.20 (s, 3 H, C-H) ppm.

^{13}C{^{1}H}-NMR (125.8 MHz, $CDCl_3$): δ = 171.54, 159.38, 148.76, 145.50, 138.37, 127.67, 126.78, 118.86, 114.57, 70.60, 55.54, 21.22 ppm.

MALDI-HRMS: *m/z* calcd. for $C_{25}H_{20}O_4S_2$ [M·]$^+$ 448.08030, found 448.07886 (Δm = 0.00144, error 3.2 ppm).

Synthesis of 2,6-Bis(4-nitrophenyl)-4H-cyclopenta[1,2-b:5,4-b']dithiophene-4-one (**10**, **S17**)

Synthesis was done as described in general procedure D with 4-nitroboronic acid. Starting from compound S14 (100 mg, 0.29 mmol), 75 mg (60%) of 10 was obtained as red-brown solid.

Yield: 0.075 g (60%)

TLC (DCM): R_f = 0.65

^{1}H-NMR (250 MHz, $CDCl_3$): δ = 8.28 (d, $^3J_{H-H}$ = 8.8 Hz, 4 H, Ar-H), 7.69 (d, $^3J_{H-H}$ = 8.8 Hz, 4 H, Ar-H), 7.43 (s, 2 H, Ar-H) ppm.

Synthesis of 2,6-Bis(4-nitrophenyl)-4H-cyclopenta[1,2-b:5,4-b']dithiophene-4-ol (**16**, **S20**)

Synthesis was done as described in general procedure A. Starting from compound S17 (70 mg, 0.16 mmol), 30 mg (43%) of 16 was obtained.

Yield: 0.03 g (43%)

TLC (DCM): R_f = 0.08

^{1}H-NMR (250 MHz, DMSO-d$_6$): δ = 8.26 (d, $^3J_{H-H}$ = 8.8 Hz, 4 H, Ar-H), 8.00-7.91 (m, 6 H, Ar-H), 6.04 (d, $^3J_{H-H}$ = 8.2 Hz, 1 H, O-H), 5.44 (d, $^3J_{H-H}$ = 8.2 Hz, 1 H, C-H) ppm.

Synthesis of 2,6-Bis(4-nitrophenyl)-4H-cyclopenta[1,2-b:5,4-b']dithiophene-4-yl-acetate (**21**)

Synthesis was done as described in general procedure C. Starting from compound S20 (30 mg, 0.07 mmol), 6 mg (18%) of 21 was obtained.

Yield: 0.006 g (18%)

TLC (cyclohexane:EtOAc 2:1): R_f = 0.48

^{1}H-NMR (500 MHz, $CDCl_3$): δ = 8.26 (d, $^3J_{H-H}$ = 8.9 Hz, 4 H, Ar-H), 7.72 (d, $^3J_{H-H}$ = 8.9 Hz, 4 H, Ar-H), 7.54 (s, 2 H, Ar-H), 6.29 (s, 1 H, C-H), 2.23 (s, 3 H, C-H) ppm.

$^{13}C\{^1H\}$-NMR (125.8 MHz, $CDCl_3$): δ = 171.40, 150.54, 146.79, 140.63, 127.93, 125.50, 124.80, 122.54, 114.16, 70.25, 21.11 ppm.

MALDI-MS: *m/z* calcd. for $C_{23}H_{14}N_2O_6S_2$ $[M\cdot]^+$ 478.03, found 477.98.

Synthesis of 2-Bromo-6-(4-methoxyphenyl)-4H-cyclopenta[1,2-b:5,4-b']dithiophene-4-one (**8**, **S21**)

Synthesis was done as described in general procedure D with 4-methoxyboronic acid. Starting from compound S14 (100 mg, 0.29 mmol), 30 mg (28%) of product 8 was obtained.

Yield: 0.03 g (28%)

TLC (DCM:cyclohexane 2:1): R_f = 0.61

^1H-NMR (600 MHz, $CDCl_3$): δ = 7.45 (d, $^3J_{H-H}$ = 8.9 Hz, 2 H, Ar-H), 7.08 (s, 1 H, Ar-H), 7.00 (s, 1 H, Ar-H), 6.92 (d, $^3J_{H-H}$ = 8.9 Hz, 2 H, Ar-H), 3.84 (s, 3 H, CH_3) ppm.

Synthesis of 2-Bromo-6-(4-methoxyphenyl)-4H-cyclopenta[1,2-b:5,4-b']dithiophene-4-ol (**14**, **S22**)

Synthesis was done as described in general procedure A. Starting from compound S21 (30 mg, 0.08 mmol), 27 mg (71%) of product 14 was obtained.

Yield: 0.027 g (71%)

TLC (cyclohexane:EtOAc 9:1): R_f = 0.05

^1H-NMR (250 MHz, DMSO-d_6): δ = 7.58 (d, $^3J_{H-H}$ = 8.8 Hz, 2 H, Ar-H), 7.44 (s, 1 H, Ar-H), 7.29 (s, 1 H, Ar-H), 6.99 (d, $^3J_{H-H}$ = 8.8 Hz, 2 H, Ar-H), 5.84 (d, $^3J_{H-H}$ = 7.7 Hz, 1 H, O-H), 5.26 (d, $^3J_{H-H}$ = 7.8 Hz, 1 H, C-H), 3.79 (s, 3 H, CH_3) ppm.

Synthesis of 2-Bromo-6-(4-methoxyphenyl)-4H-cyclopenta[1,2-b:5,4-b']dithiophene-4-yl-acetate (**18**)

Synthesis was done as described in general procedure C. Starting from compound S22 (27 mg, 0.06 mmol), 8 mg (33%) of product 18 was obtained.

Yield: 0.008 g (33%)

TLC (cyclohexane:EtOAc 8:1): R_f = 0.31

^1H-NMR (250 MHz, DMSO-d_6): δ = 7.57 (d, $^3J_{H-H}$ = 8.8 Hz, 2 H, Ar-H), 7.36 (s, 1 H, Ar-H), 7.24 (s, 1 H, Ar-H), 6.99 (d, $^3J_{H-H}$ = 8.8 Hz, 2 H, Ar-H), 6.23 (s, 1 H, C-H), 3.79 (s, 3 H, CH_3), 2.14 (s, 3 H, CH_3) ppm.

^1H-NMR (500 MHz, $CDCl_3$): δ = 7.49 (d, $^3J_{H-H}$ = 8.8 Hz, 2 H, Ar-H), 7.19 (s, 1 H, Ar-H), 7.11 (s, 1 H, Ar-H), 6.91 (d, $^3J_{H-H}$ = 8.8 Hz, 2 H, Ar-H), 6.15 (s, 1 H, C-H), 3.84 (s, 3 H, CH_3), 2.18 (s, 3 H, CH_3) ppm.

$^{13}C\{^1H\}$-NMR (125.8 MHz, $CDCl_3$): δ = 171.36, 159.56, 148.73, 146.66, 146.23, 140.65, 137.43, 127.41, 126.89, 126.78, 118.81, 114.61, 111.57, 70.44, 55.55, 21.11 ppm.

MALDI-HRMS: *m/z* calcd. for $C_{18}H_{13}BrO_3S_2$ $[M\cdot]^+$ 419.94895, found 419.94814 (Δm = 0.00081, error 1.9 ppm).

3. Photochemical Measurements

Molar absorption coefficient measurements

A stock solution of each compound was prepared by weighing a small amount of each sample (approx. 1.0 mg) in a glass vial and dissolving it in 2 mL of a 20% 0.1 M PBS-buffer 80% MeOH mixture. Then a dilution series from this stock solution was prepared. Absorbance spectra in wavelength range 300-800 nm were measured for all solutions. The data analysis was started by baseline correction. This was done by subtracting the average value of the baseline shift in the wavelength range where the sample does not absorb (in this case 600-800 nm) from the entire spectra. Then the linearity of the measured data was checked by plotting absorbance vs. concentration.

Live-tracking of Photolysis behavior

Several 10-150 µM solutions (80% MeOH : 20% 0.1 M PBS Buffer) were irradiated at 365 nm (700 mA, 11.2 mW) or at 455 nm (9.9 mW, 1000 mA) and its absorption was “live” tracked during irradiation via our in house system PHITS for selected compounds. Left side: absorption spectra before (black) and after irradiation (red). Right: Absorption change during irradiation monitored at different wavelengths.

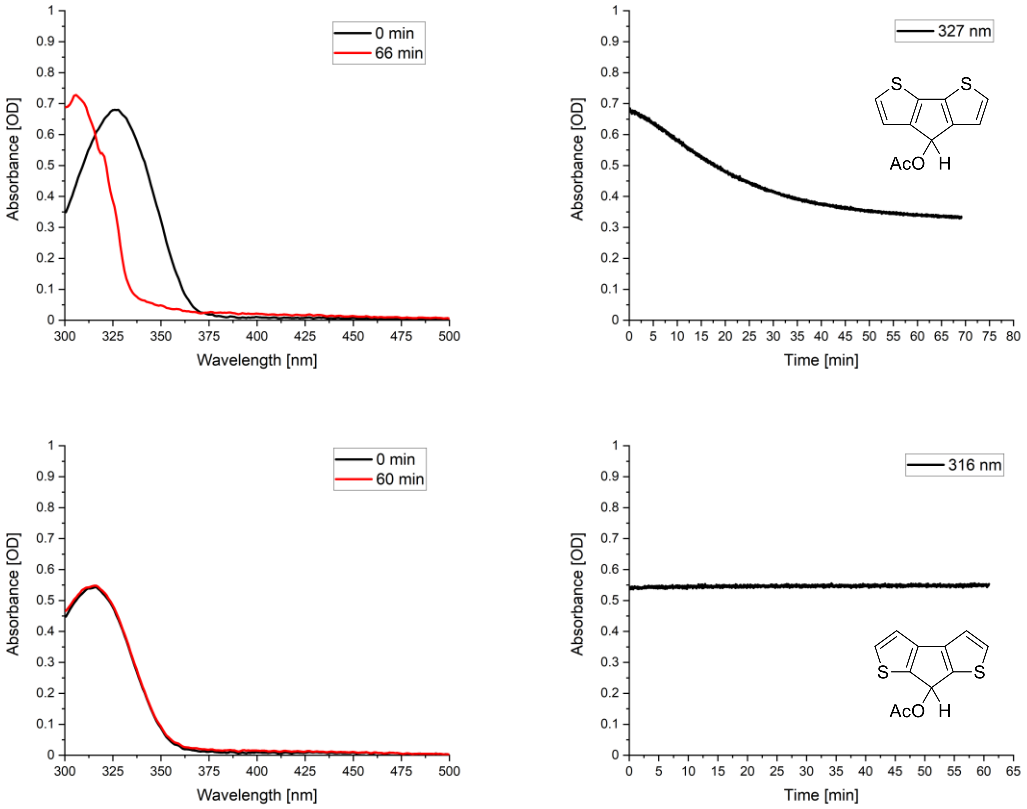

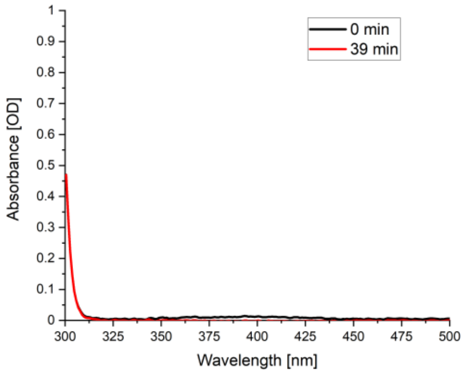
Absorbance [OD]
Wavelength [nm]
0 min
39 min

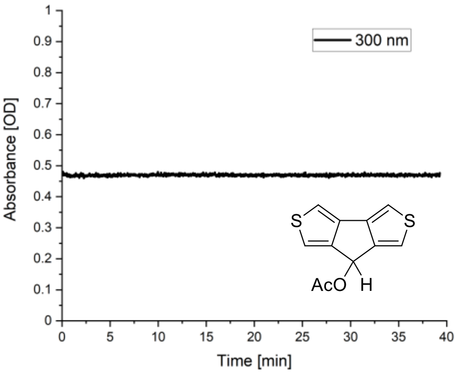
Absorbance [OD]
Time [min]
300 nm
S
S
AcO H

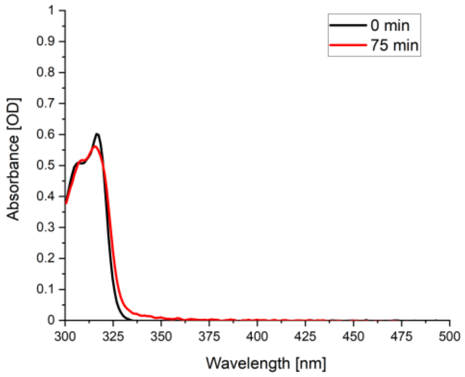
Absorbance [OD]
Wavelength [nm]
0 min
75 min

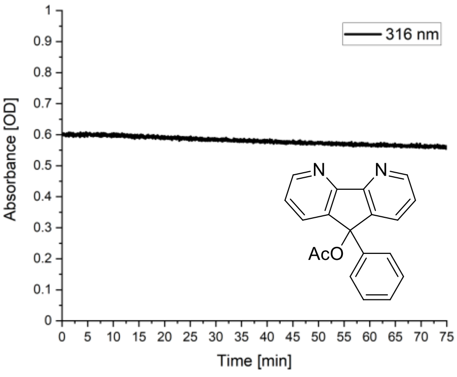
Absorbance [OD]
Time [min]
316 nm
N
N
AcO

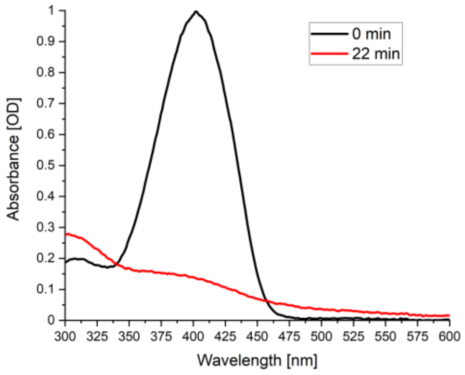
Absorbance [OD]
Wavelength [nm]
0 min
22 min

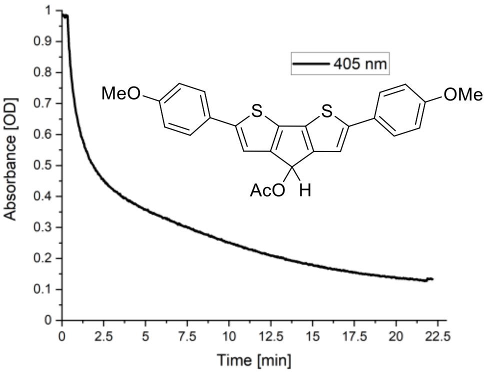
Absorbance [OD]
Time [min]
405 nm
MeO
S
S
OMe
AcO H

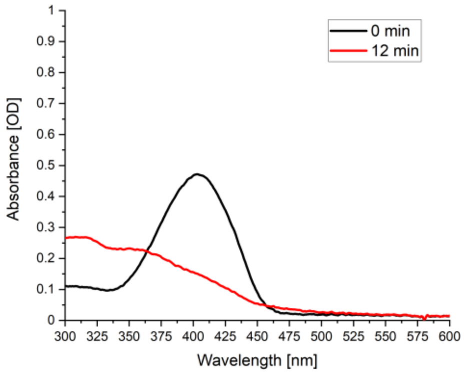
Absorbance [OD]
Wavelength [nm]
0 min
12 min

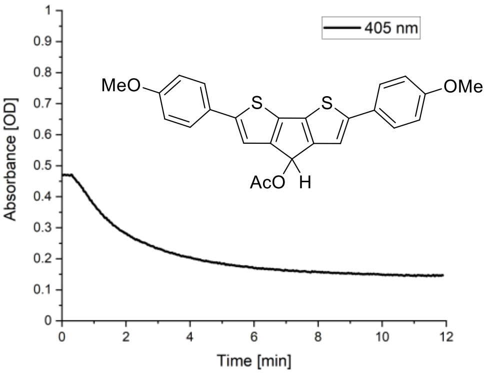
Absorbance [OD]
Time [min]
405 nm
MeO
S
S
OMe
AcO H

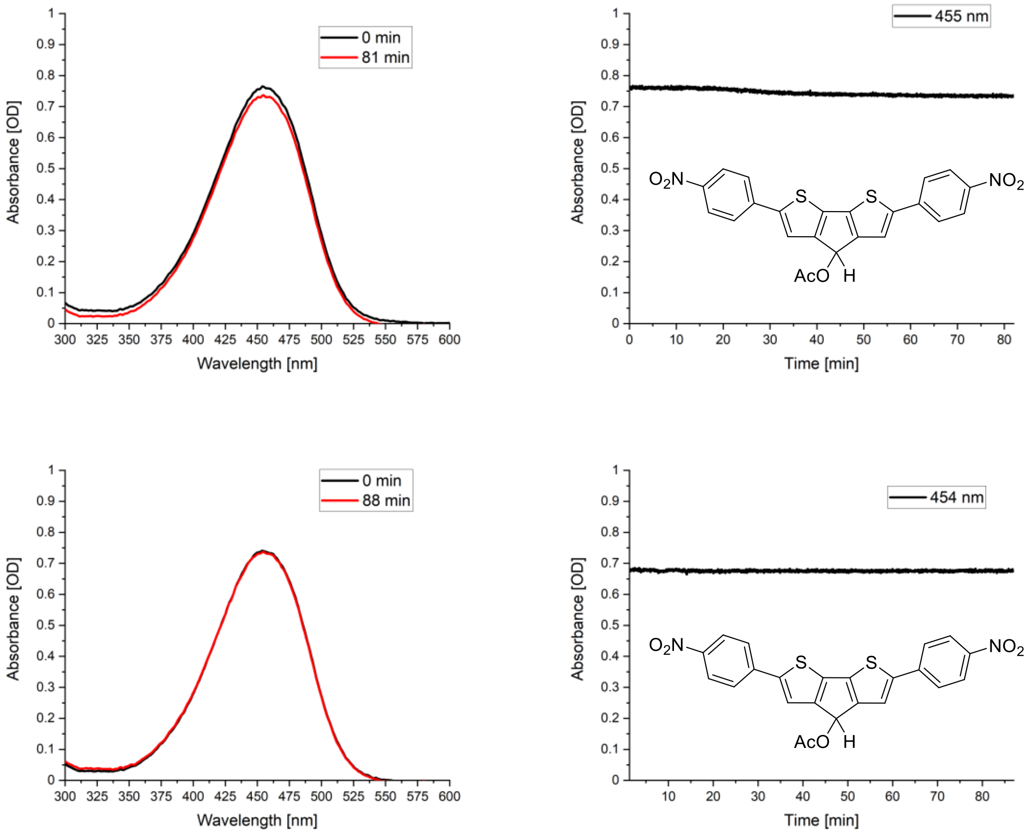

Figure S1: Photolysis and absorption behavior for selected compounds during irradiation at 310 nm, 365 nm or 405 nm.

4-Phenyl-4H-cyclopenta[1,2-b:5,4-b']dithiophene-4-yl-acetate (**1c**) was irradiated for 1.5 h (365 nm, 700 mA, 11.2 mW) in a mixture of MeOD-δ_4 and D_2O und (4:1). Red: bevor irradiation. Blue: After irradiation. It showed no thermal degradation after 2 days in the dark in the same solvent mixture.

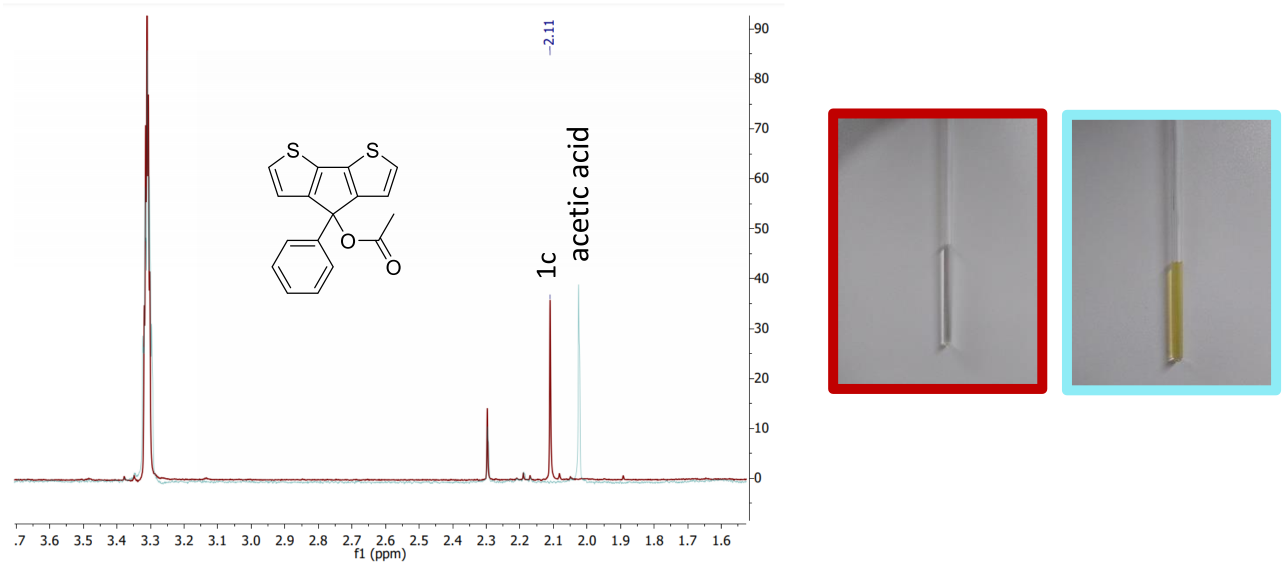

Figure S2: Photolysis observed via ^{1}H NMR (left) and color change after photolysis in NMR tube (right).

Determination of the Quantum yields

Table S1: HPLC-solvent gradients used for determination of the quantum yields.

Gradient 1		Gradient 2	
Time (min)	Solvent A (%)	Time (min)	Solvent A (%)
0 to 3	70	0 to 5	5
3 to 13	70 to 100	5 to 10	5 to 80
13 to 25	100	10 to 20	80 to 100
		20 to 22	100

Gradient 1: Solvent A – MeCN; solvent B – ultra pure water + 0.1% TFA

Gradient 2: Solvent A – MeCN; solvent B – ultra pure water + 0.1% TFA

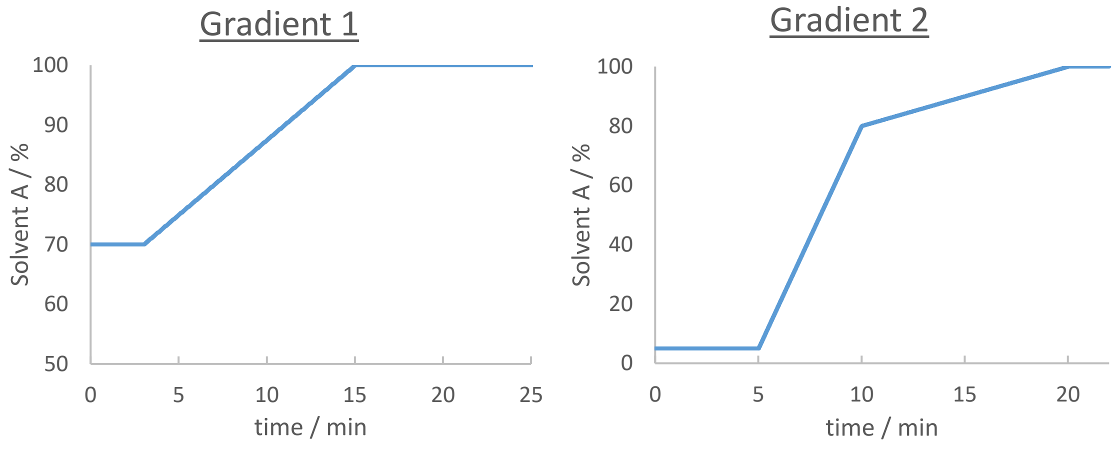

Figure S3: HPLC gradients.

Three stock solutions of each compound were prepared by weighting small amounts of each sample (approx. 1.0 mg) in three different glass vials and dissolving each probe with 2 mL dimethylsulfoxide. An aliquot of each stock solution was dissolved with 0.1 M PBS-buffer (13.7 mM NaCl, 0.27 mM KCl, 1 mM Na_2HPO_4, 0.18 mM KH_2PO_4) and if precipitation occured, additional DMSO was added. From each sample (three samples for each compound), 50 µL were taken and irradiated at nine different irradiation times (t = 0 s, 5 s, 10 s, 20 s, 45 s, 90 s, 180 s, 360 s, 720 s) with the respective LED (see General information) resulting in 27 different irradiated solutions. The photon flux for this setup was determined with a robust fulgide-derivative as described in literature.[1] The photolysis of the starting material was analyzed via RP-HPLC as the ratio of the peak areas of the internal standard and the starting material (detection at 254 nm) related to literature.[6] Uridine was used as an internal standard. An example is shown in figure S4 and S5.

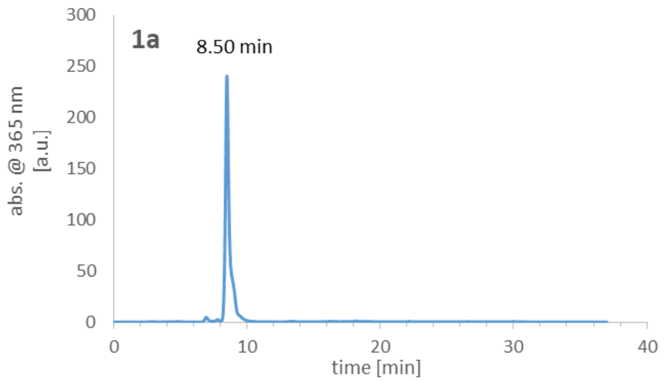
1a
8.50 min
abs. @ 365 nm [a.u.]
time [min]

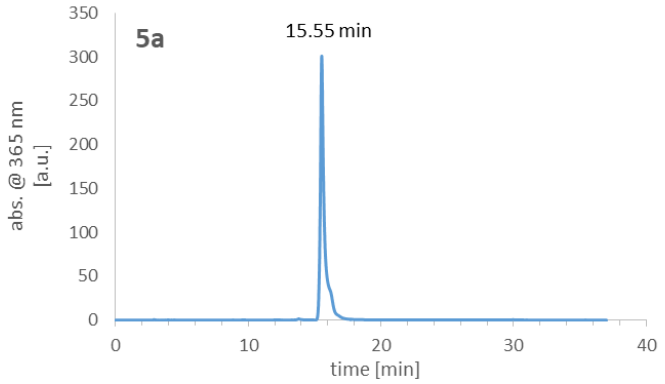
5a
15.55 min
abs. @ 365 nm [a.u.]
time [min]

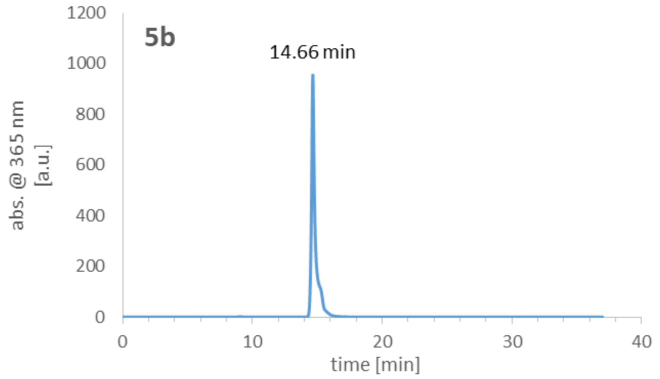
5b
14.66 min
abs. @ 365 nm [a.u.]
time [min]

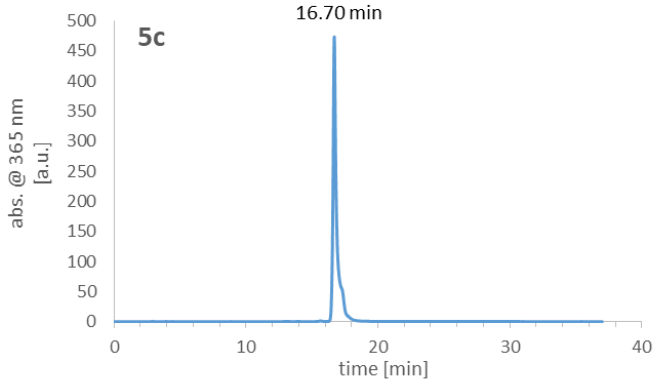
5c
16.70 min
abs. @ 365 nm [a.u.]
time [min]

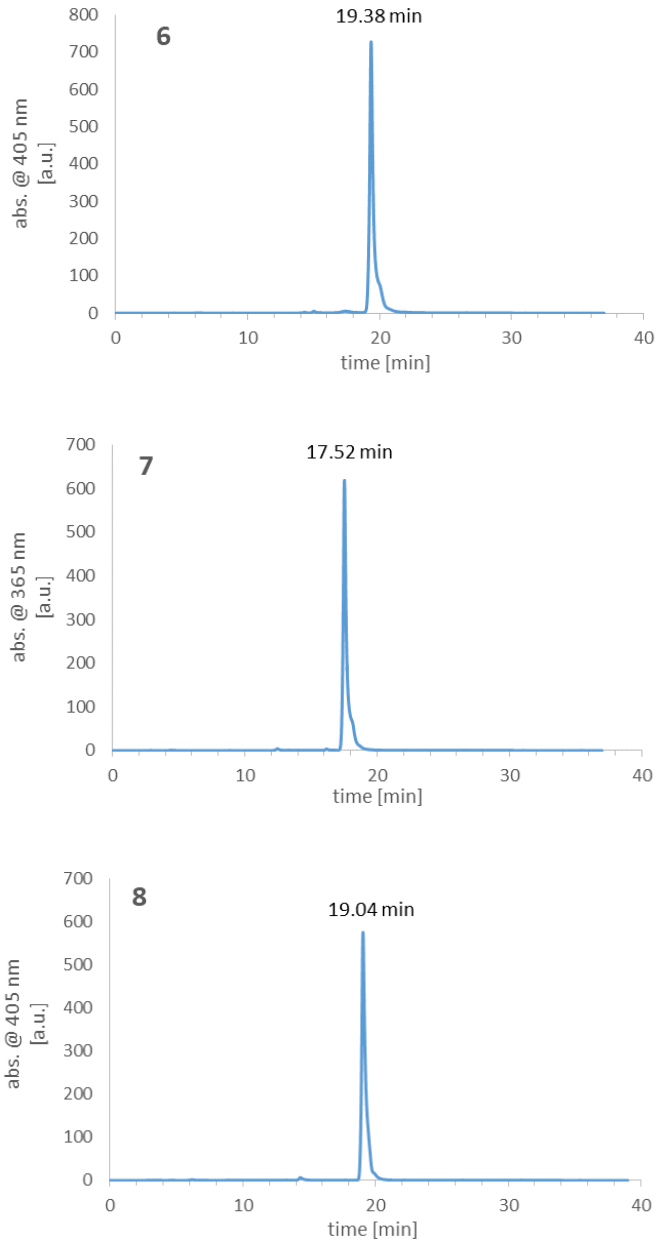

Figure S4: Analytical RP-HPLC chromatograms of selected compounds.

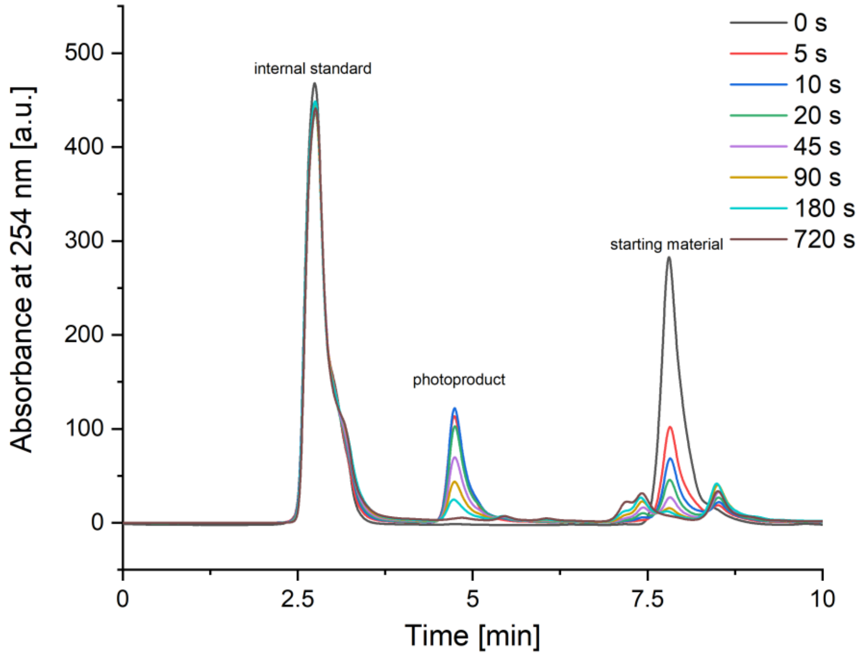

Figure S5: Photolysis observed for compound **1b** via HPLC after several irradiation times.

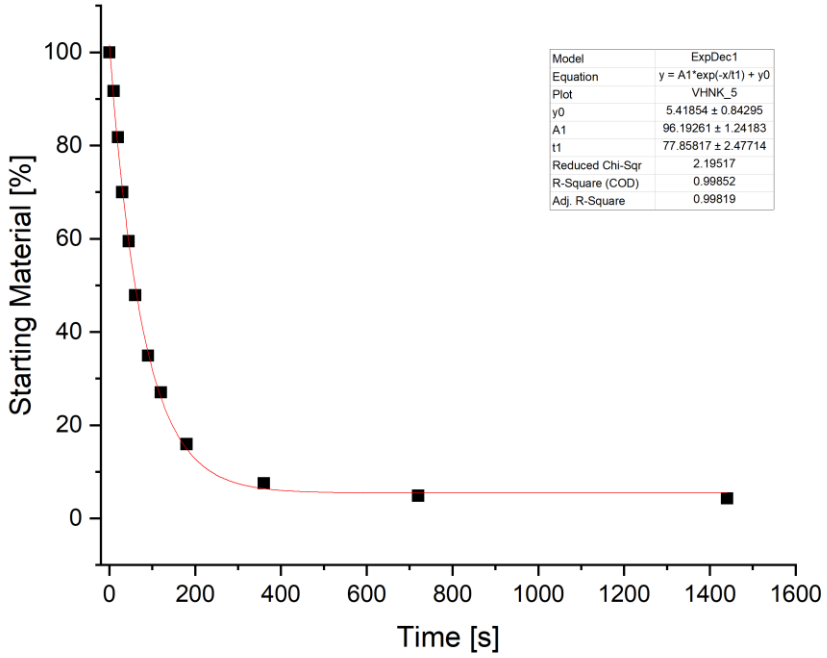

Figure S6: Amount of starting material (%) at different irradiation times and corresponding decay function.

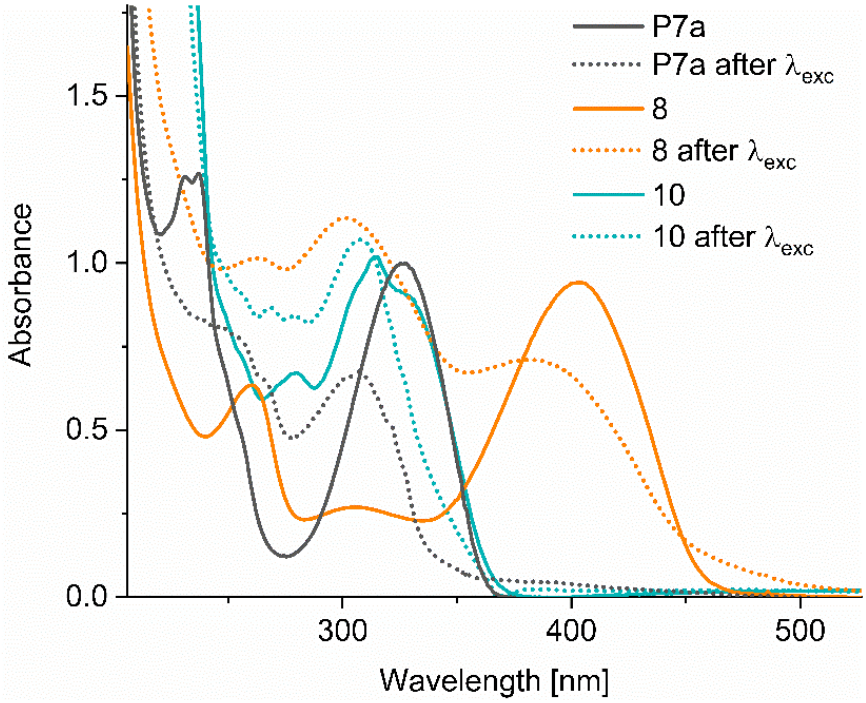

Figure S7: Absorption spectra in MeOH before and after 8 minutes of illumination at 365 nm for **P7a** and **10** and 420 nm for **8** (normalized to the main peak of the dark spectrum @ 327 nm and 405 nm respectively).

4. Theoretical calculations

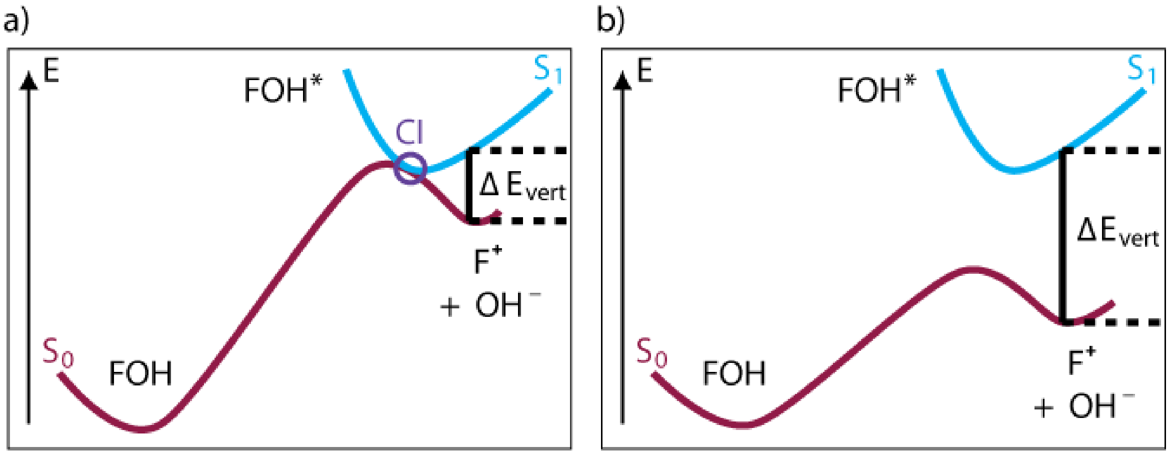

Figure S8: Illustration of computational screening procedure for fluorenol-like (FOH) compounds adapted from Winter *et al.*.[7] A productive photocage a) is photoexcited (FOH*) from the electronic ground state S0 to the first singlet excited state S1 and arrives in the product state (F^+ + OH^-) via a conical intersection (CI). This is possible due to a stabilized excited state and an energetically high lying product state. As such, the vertical absorption energy of the product, ΔE_{vert}, is expected to be rather small. On the contrary, an unproductive photocage b) with an energetically low product does not have a CI between S0 and S1 and will consequently show a large ΔE_{vert}. Thus, the smaller ΔE_{vert}, the more likely is the existence of a productive CI channel for efficient uncaging.

Table S2: Calculated vertical excitation energies of cationic species in water.

**Compound**	**Vertical Excitation Energies [eV]**
**3a** (*meta*-Thio-cation)	2.0984
**2a** (Azo-cation)	1.8029
Fluorenyl-cation	1.6131
**4a** (*ortho*-Thio-cation)	1.2141
**1a** (*para*-Thio-cation)	0.7282

Table S3: NICS(0) calculations performed at the CAM-B3LYP/def2-SVP level of theory.

**Compound**	**NICS(0) outer rings [ppm]**	**NICS(0) inner ring [ppm]**
fluorenyl cation	7.79	29.39
**2a** cation	6.97	30.69
**1a** cation	22.67	96.83
**3a** cation	0.13	15.79
**4a** cation	7.13	46.97

5. NMR spectra of key compounds

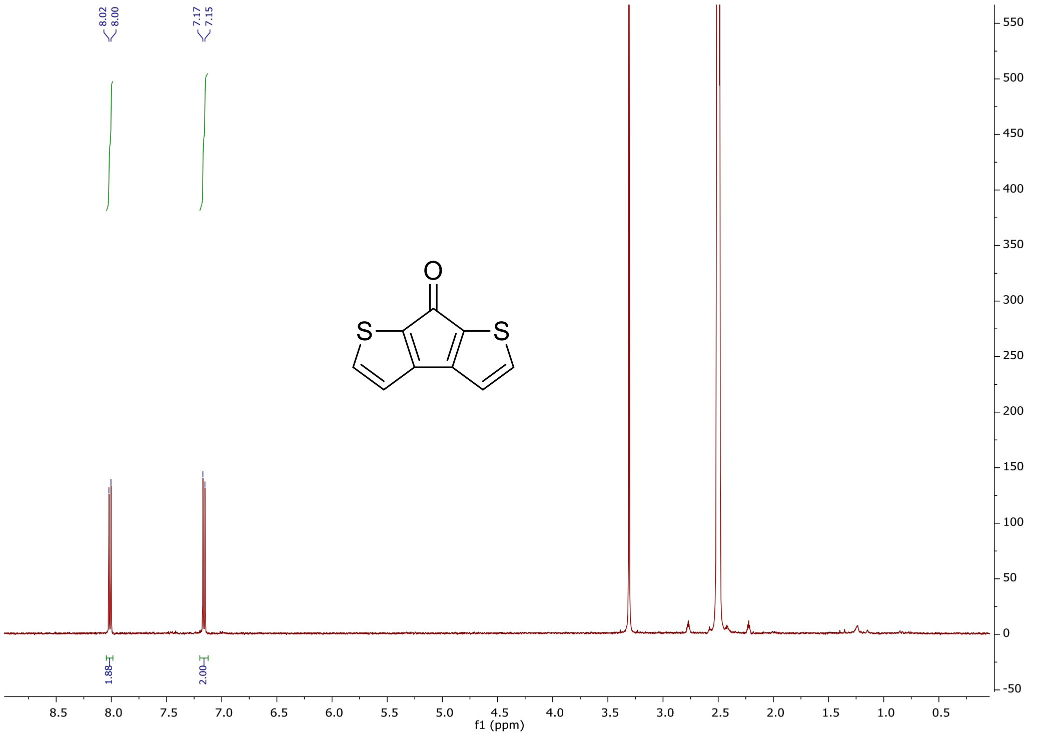

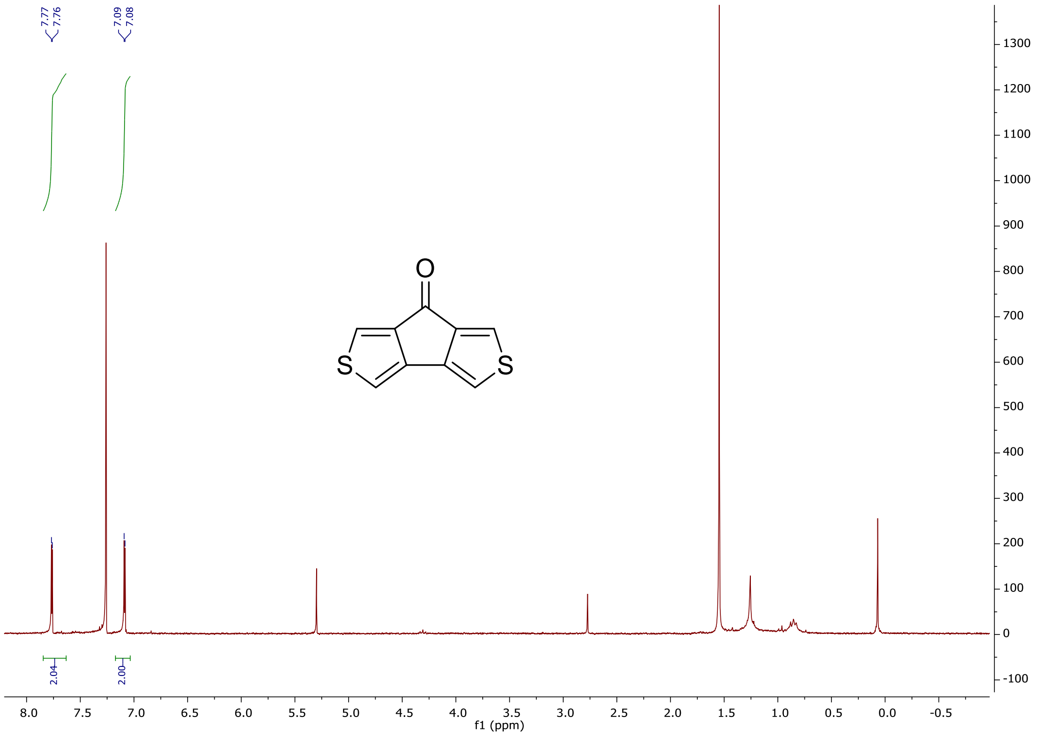

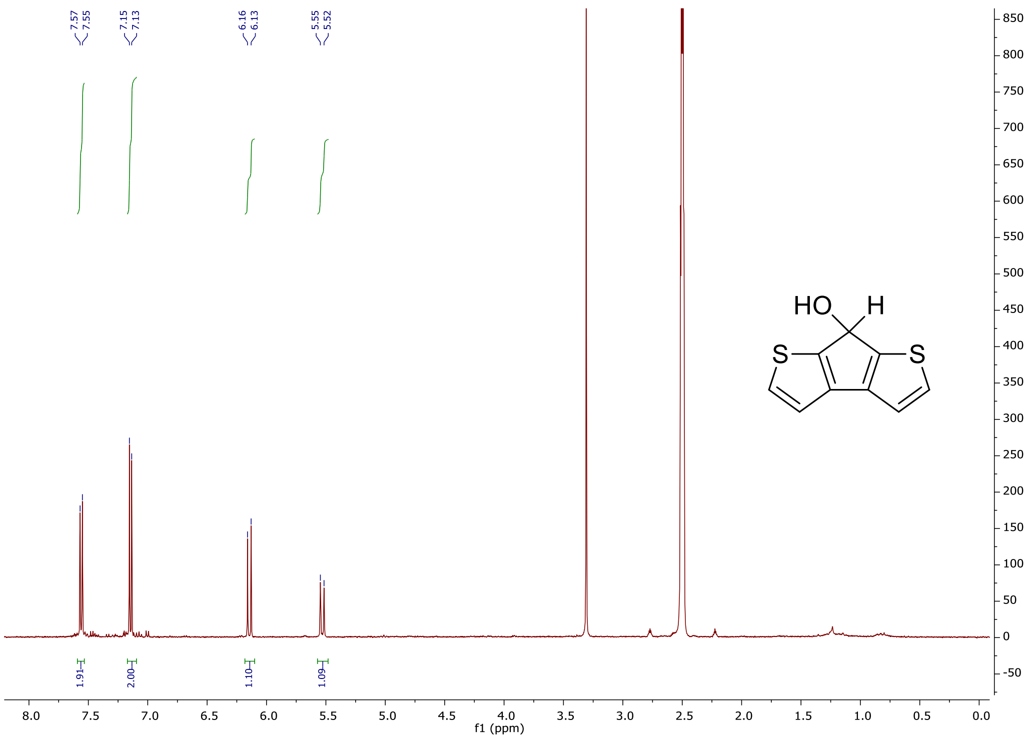

7.57
7.55
7.15
7.13
6.16
6.13
5.55
5.52
HO
H
S
S
1.91
2.00
1.10
1.09
f1 (ppm)

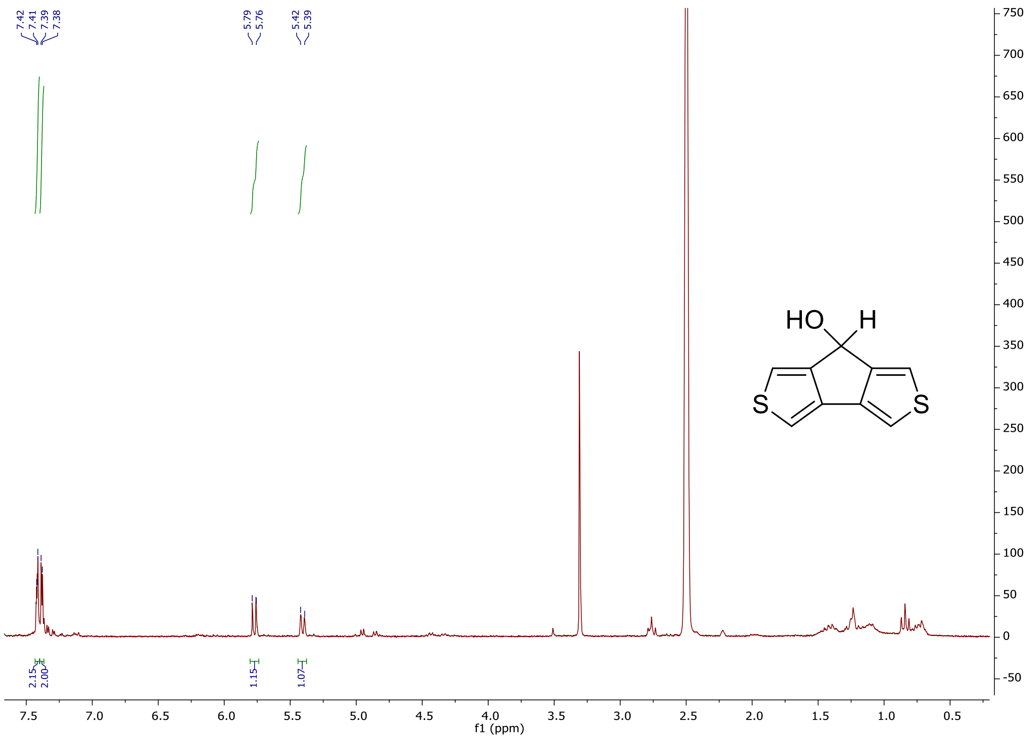

7.42
7.41
7.39
7.38
5.79
5.76
5.42
5.39
HO
H
S
S
2.15
2.00
1.15
1.07
f1 (ppm)

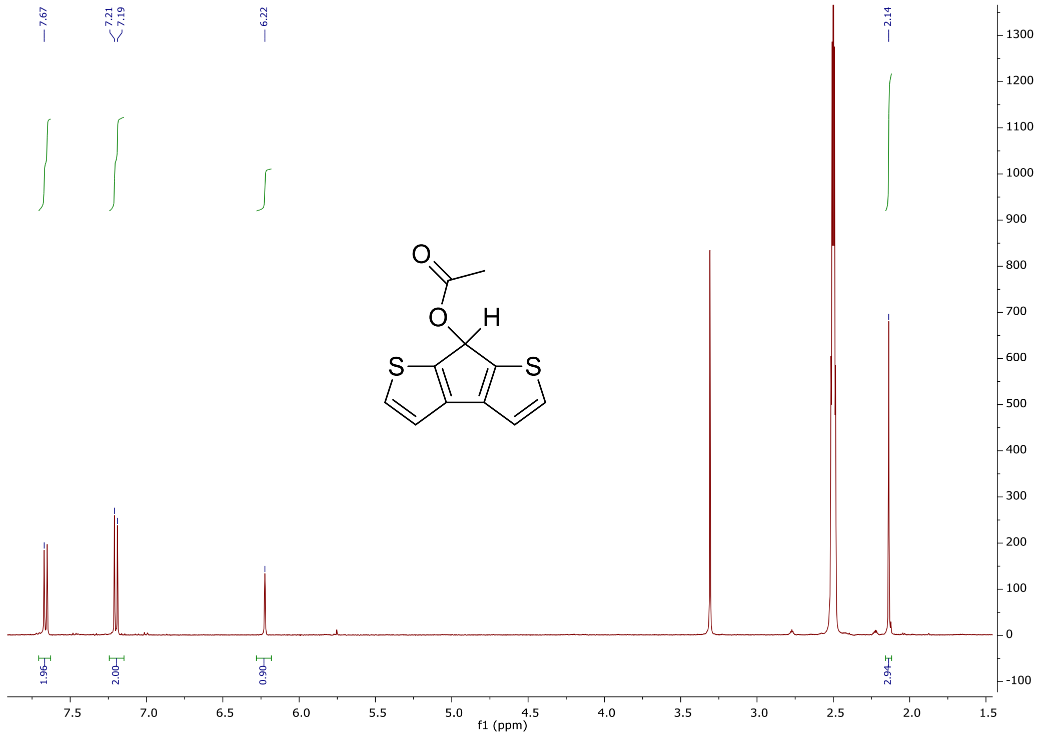

7.67
7.21
7.19
6.22
2.14
1.96
2.00
0.90
2.94
f1 (ppm)
O
O
H
S
S

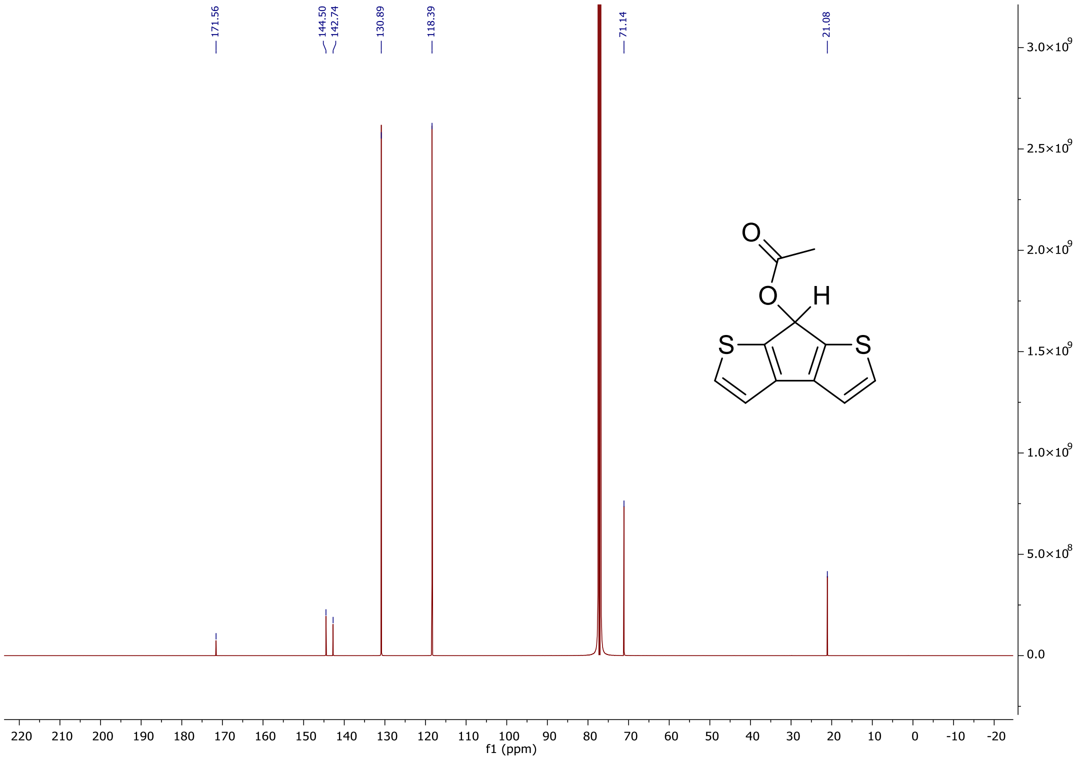

171.56
144.50
142.74
130.89
118.39
71.14
21.08
f1 (ppm)
O
O
H
S
S

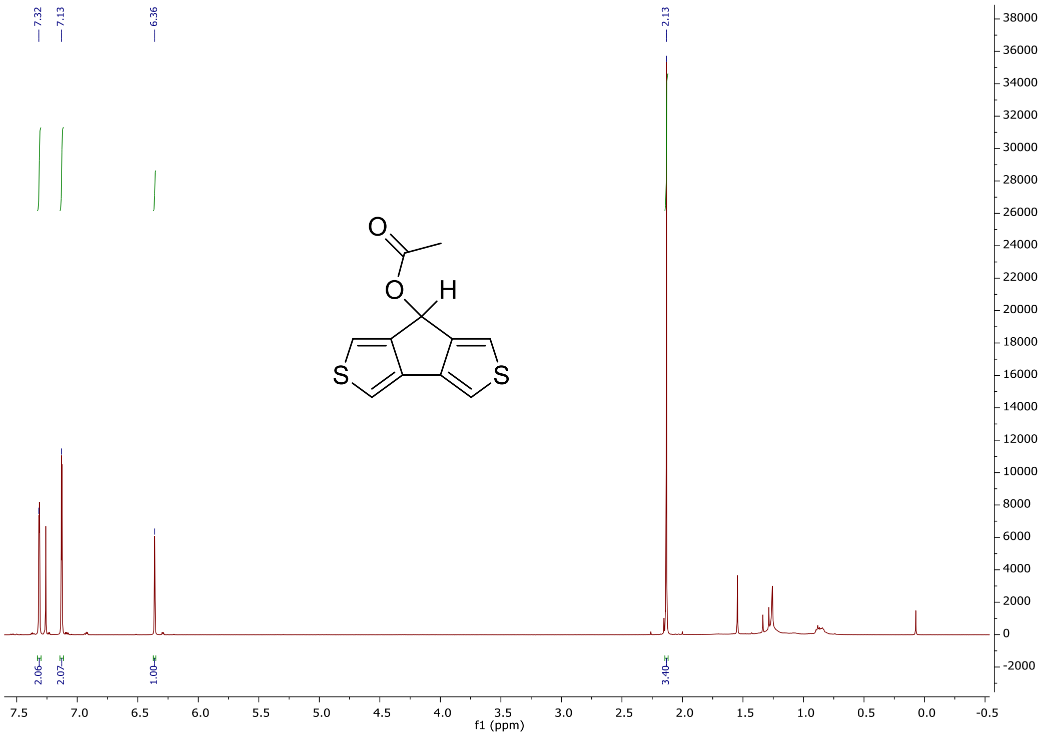
7.32
7.13
6.36
2.13
2.06
2.07
1.00
3.40
O
O
H
S
S
f1 (ppm)

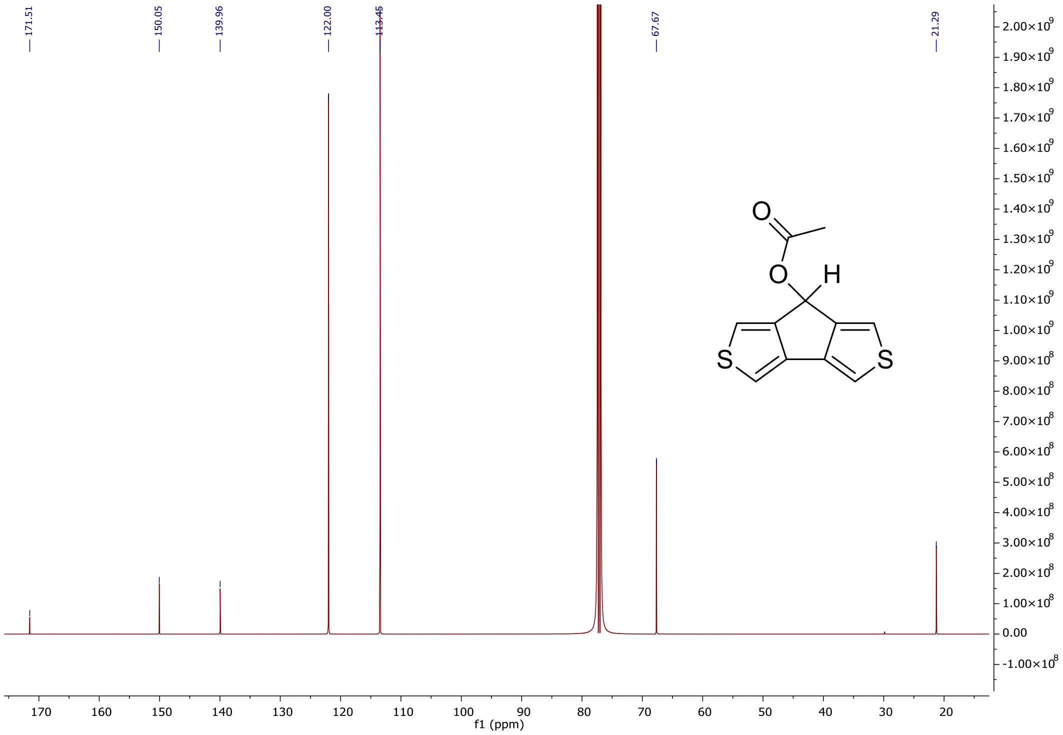
171.51
150.05
139.96
122.00
113.45
67.67
21.29
O
O
H
S
S
f1 (ppm)

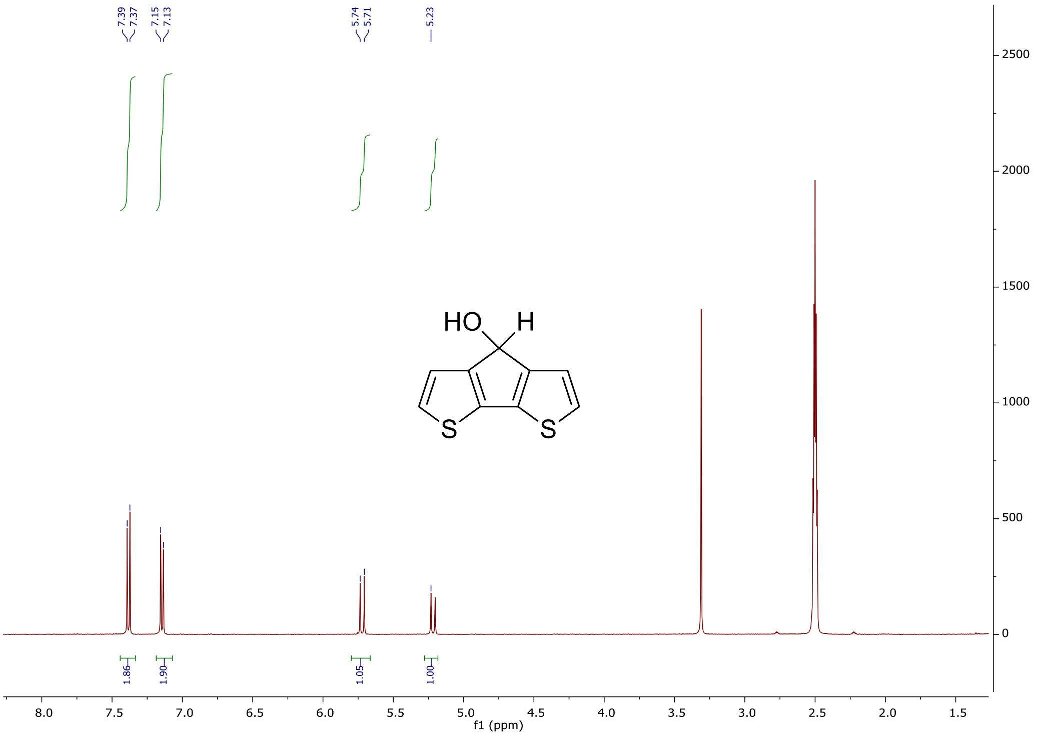
7.39
7.37
7.15
7.13
5.74
5.71
5.23
HO
H
S
S
1.86
1.90
1.05
1.00
f1 (ppm)

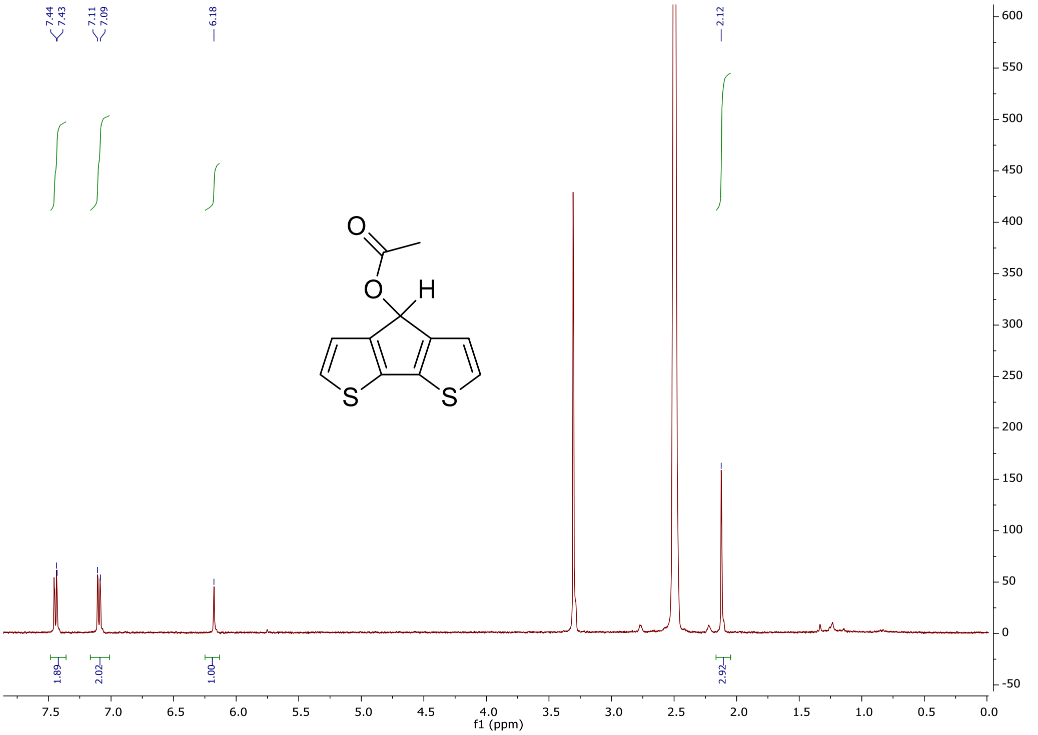
7.44
7.43
7.11
7.09
6.18
2.12
O
O
H
S
S
1.89
2.02
1.00
2.92
f1 (ppm)

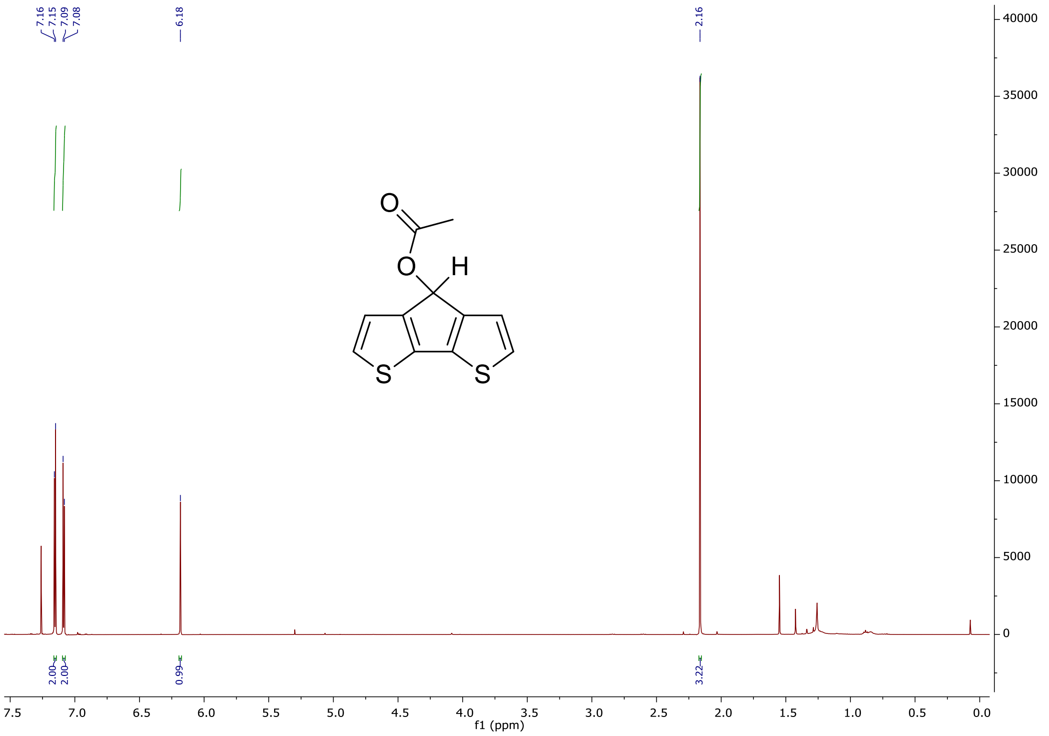
7.16
7.15
7.09
7.08
6.18
2.16
2.00
2.00
0.99
3.22
O
O
H
S
S
f1 (ppm)

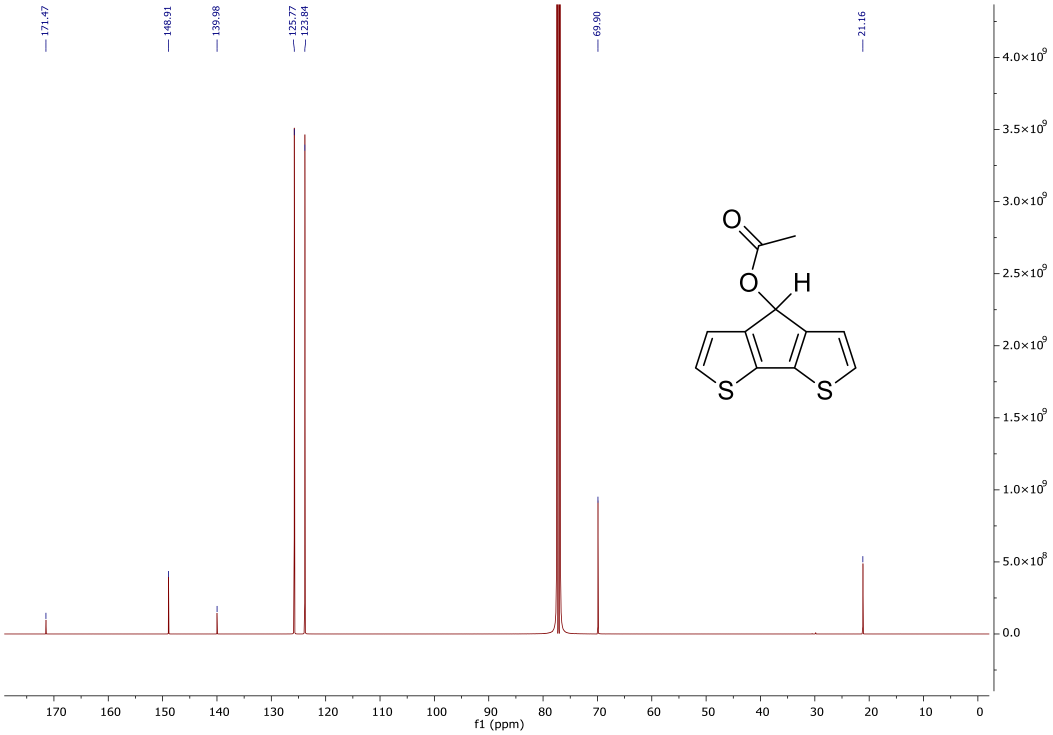
171.47
148.91
139.98
125.77
123.84
69.90
21.16
O
O
H
S
S
f1 (ppm)

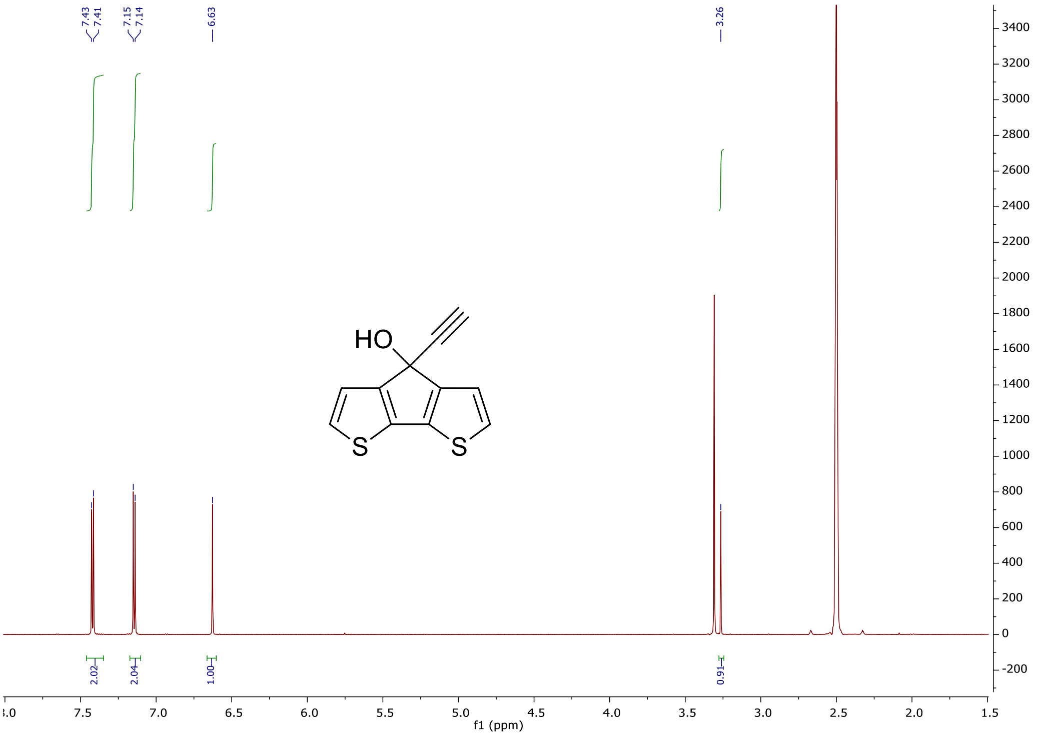
7.43
7.41
7.15
7.14
6.63
3.26
HO
S
S
2.02
2.04
1.00
0.91
f1 (ppm)

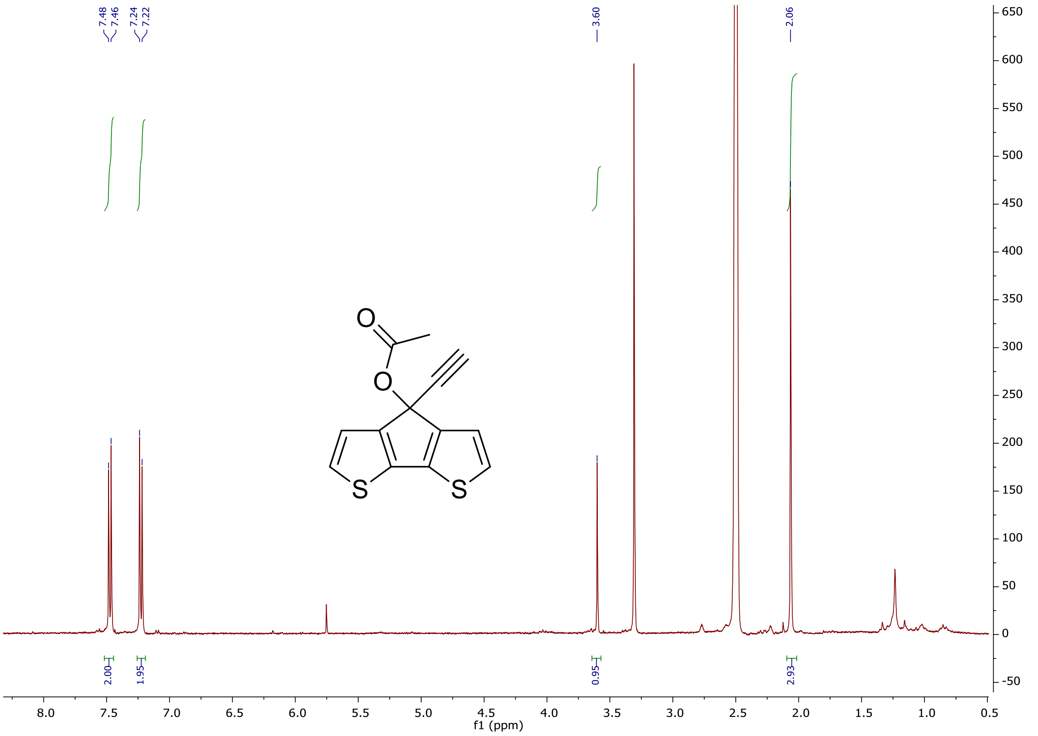
7.48
7.46
7.24
7.22
3.60
2.06
O
O
S
S
2.00
1.95
0.95
2.93
f1 (ppm)

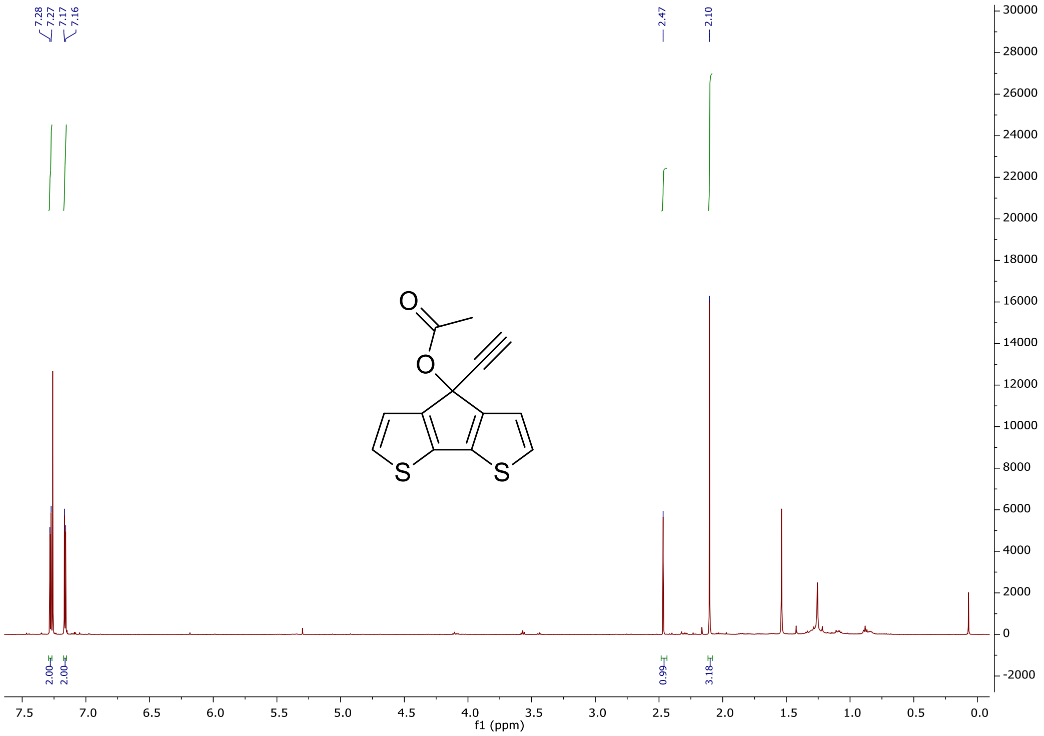
7.28
7.27
7.17
7.16
2.47
2.10
2.00
2.00
0.99
3.18
f1 (ppm)
O
O
S
S

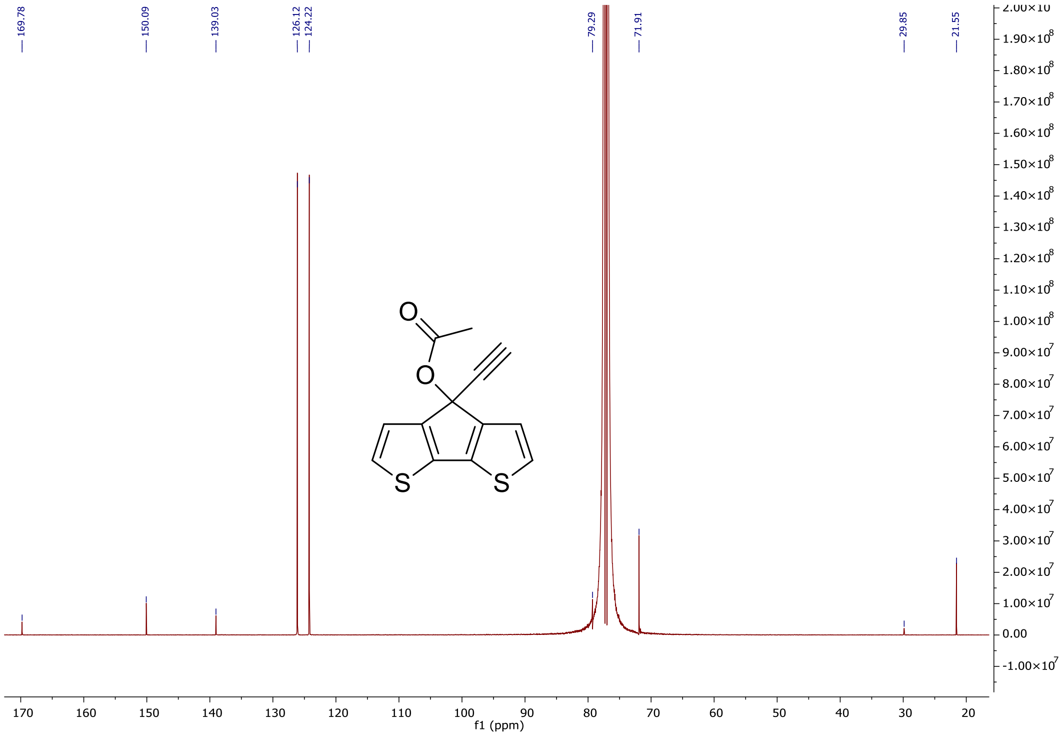
169.78
150.09
139.03
126.12
124.22
79.29
71.91
29.85
21.55
f1 (ppm)
O
O
S
S

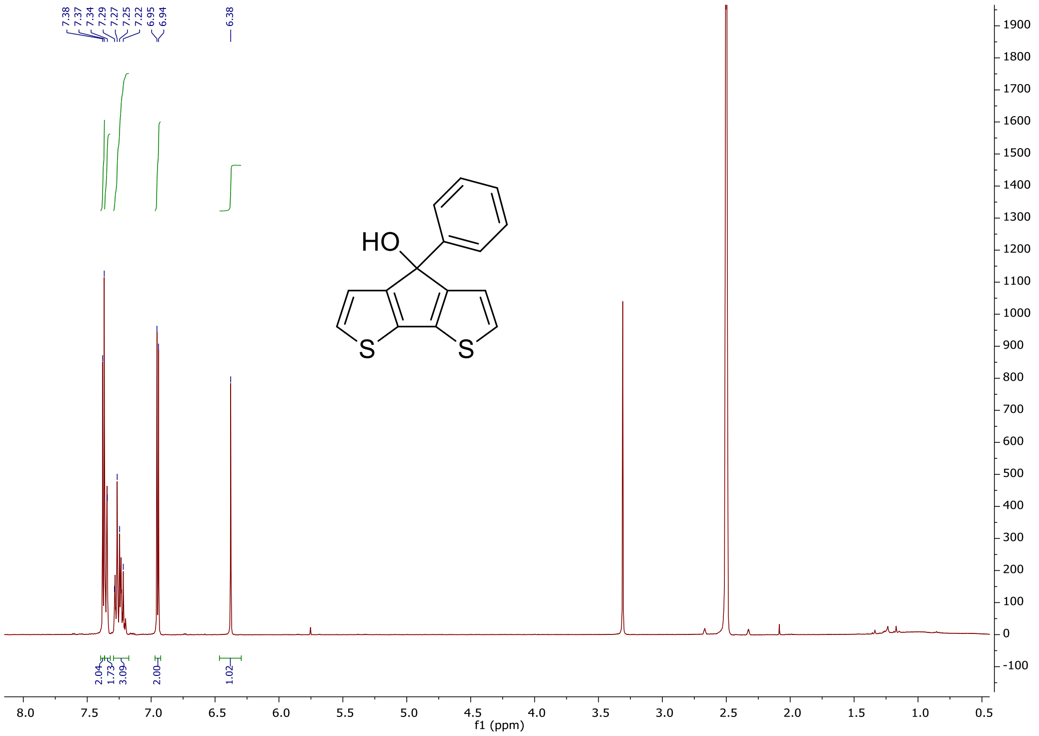
7.38
7.37
7.34
7.29
7.27
7.25
7.22
6.95
6.94
6.38
HO
S
S
2.04
1.73
3.09
2.00
1.02
f1 (ppm)

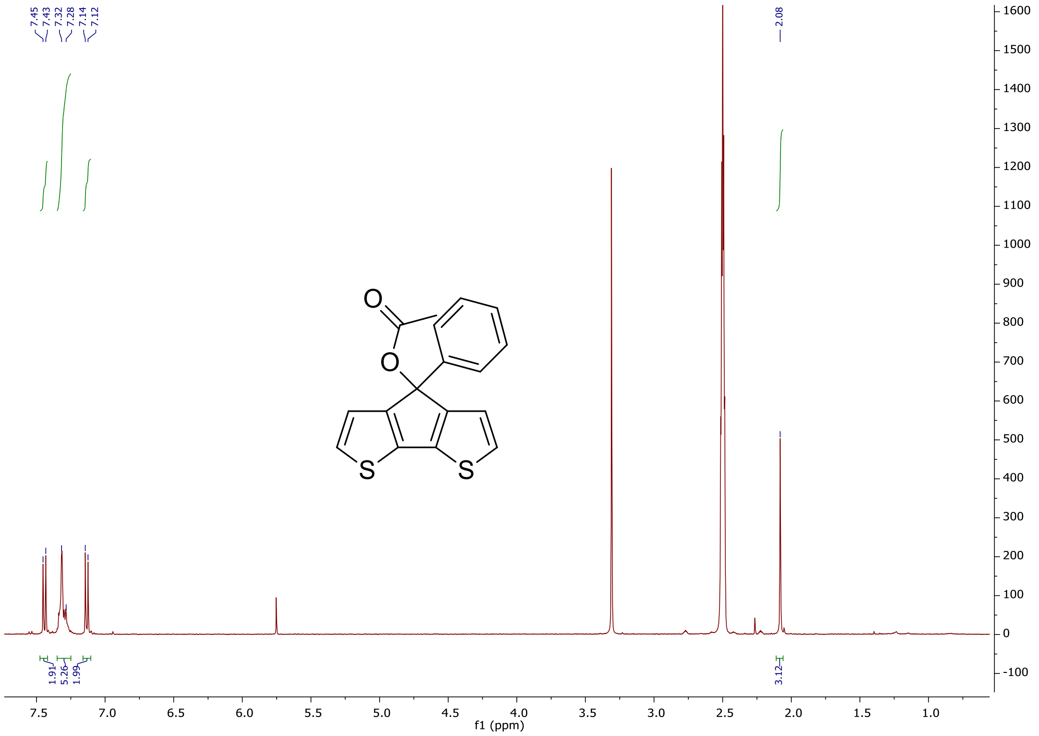
7.45
7.43
7.32
7.28
7.14
7.12
2.08
O
O
S
S
1.91
5.26
1.99
3.12
f1 (ppm)

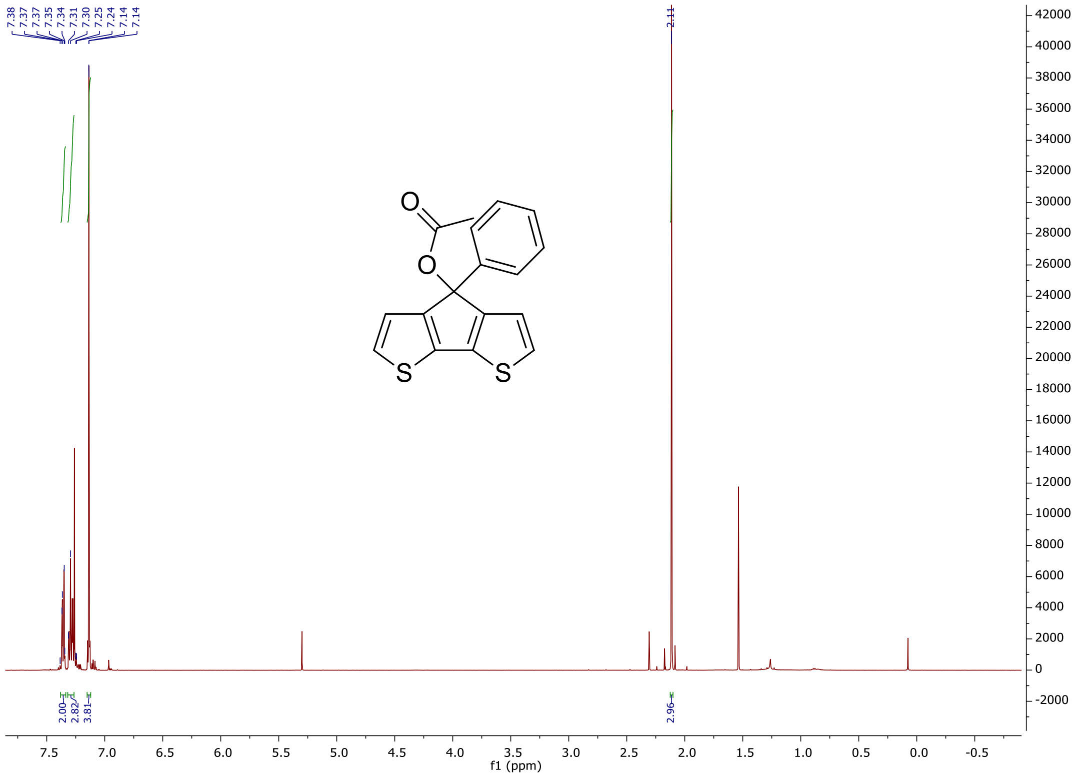
7.38
7.37
7.37
7.35
7.34
7.31
7.30
7.25
7.24
7.14
7.14
2.11
2.00
2.82
3.81
2.96
f1 (ppm)
O
O
S
S

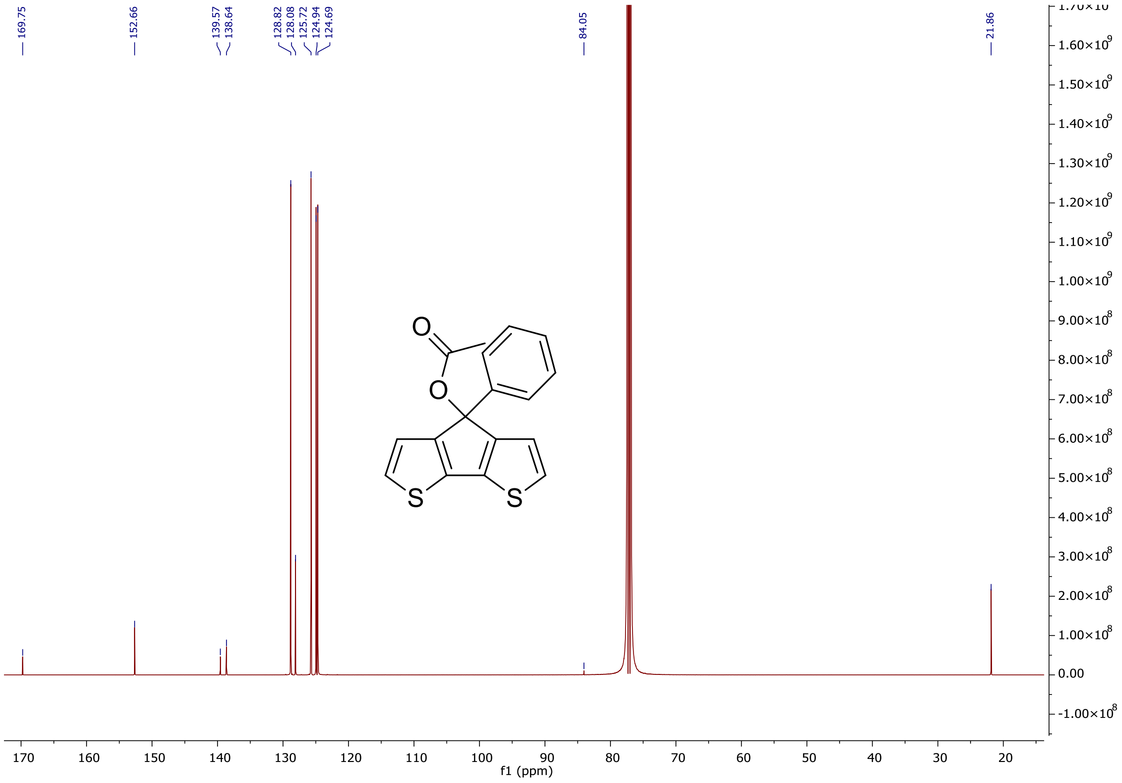
169.75
152.66
139.57
138.64
128.82
128.08
125.72
124.94
124.69
84.05
21.86
f1 (ppm)
O
O
S
S

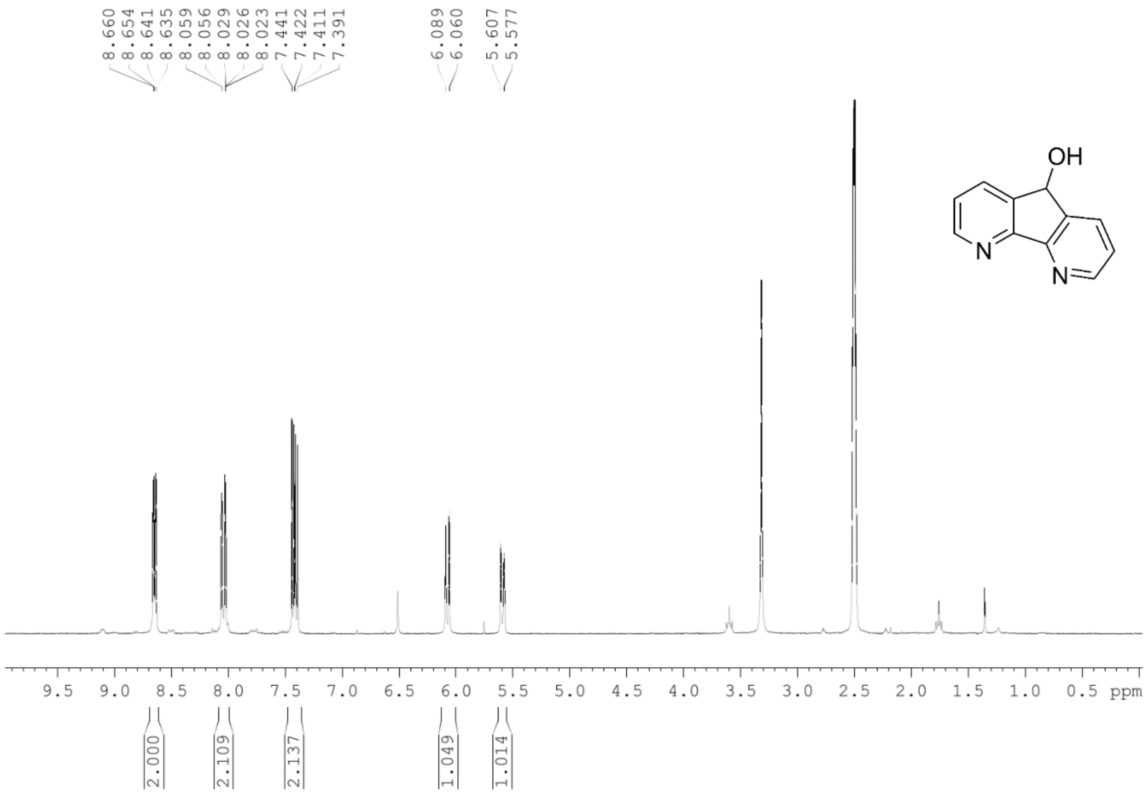
8.660
8.654
8.641
8.635
8.059
8.056
8.029
8.026
8.023
7.441
7.422
7.411
7.391
6.089
6.060
5.607
5.577
OH
N
N
9.5 9.0 8.5 8.0 7.5 7.0 6.5 6.0 5.5 5.0 4.5 4.0 3.5 3.0 2.5 2.0 1.5 1.0 0.5 ppm
2.000
2.109
2.137
1.049
1.014

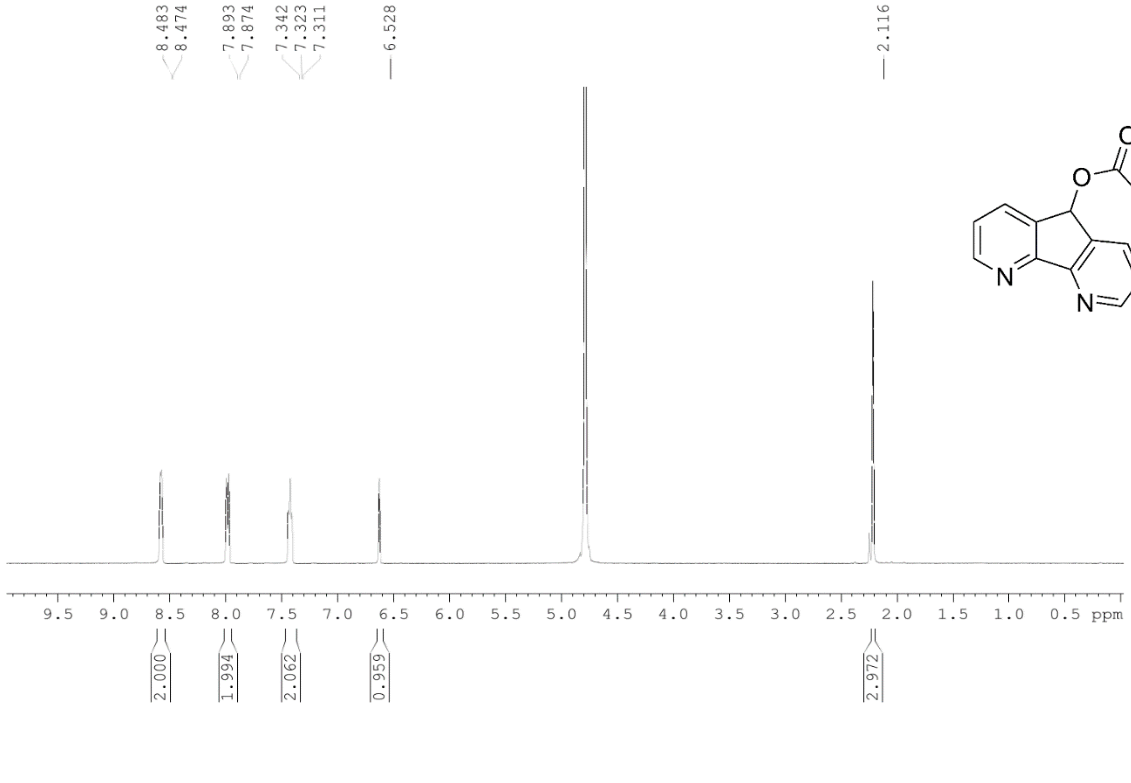
8.483
8.474
7.893
7.874
7.342
7.323
7.311
6.528
2.116
O
O
N
N
9.5 9.0 8.5 8.0 7.5 7.0 6.5 6.0 5.5 5.0 4.5 4.0 3.5 3.0 2.5 2.0 1.5 1.0 0.5 ppm
2.000
1.994
2.062
0.959
2.972

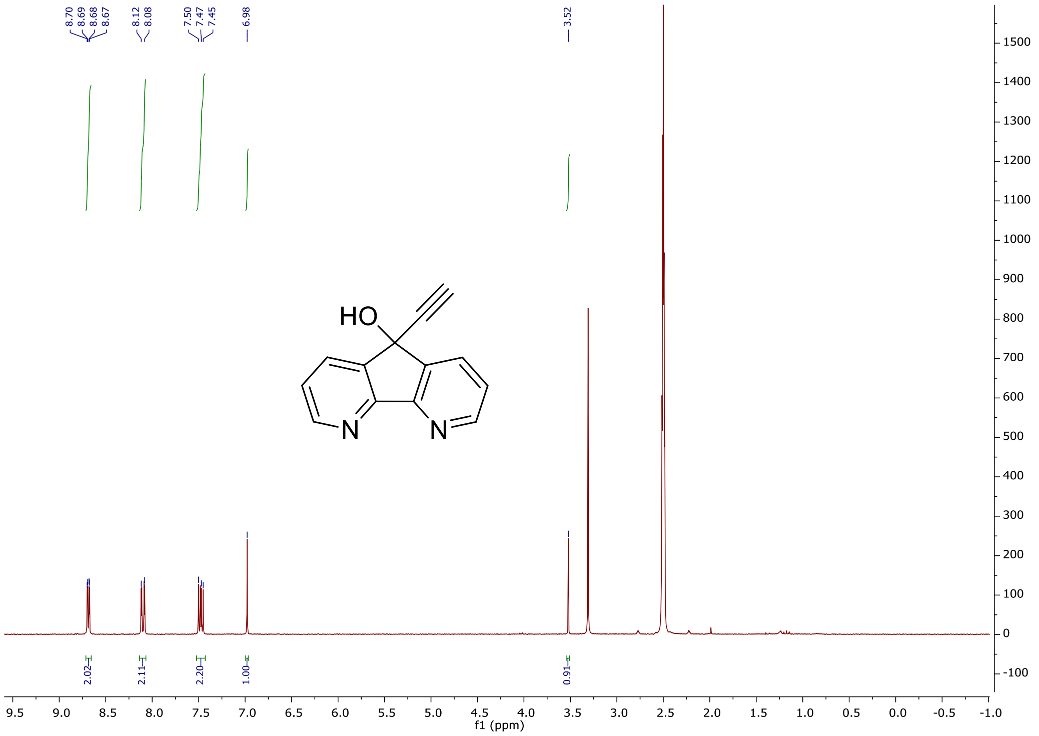

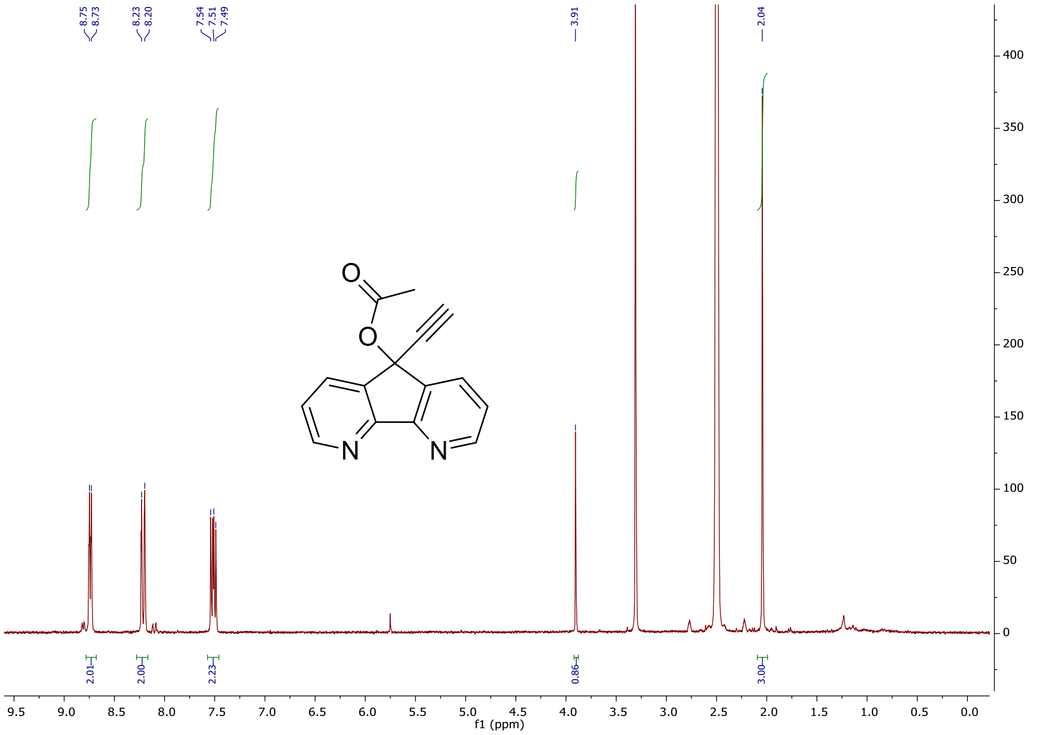

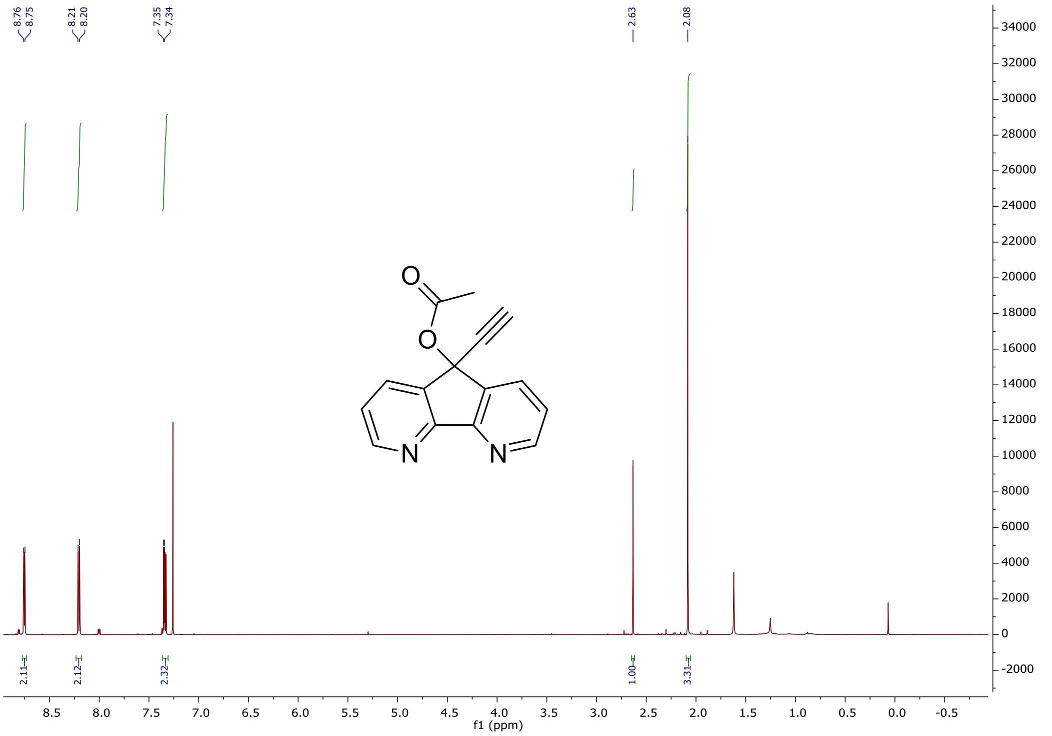
8.76
8.75
8.21
8.20
7.35
7.34
2.63
2.08
2.11
2.12
2.32
1.00
3.31
O
O
N
N
f1 (ppm)

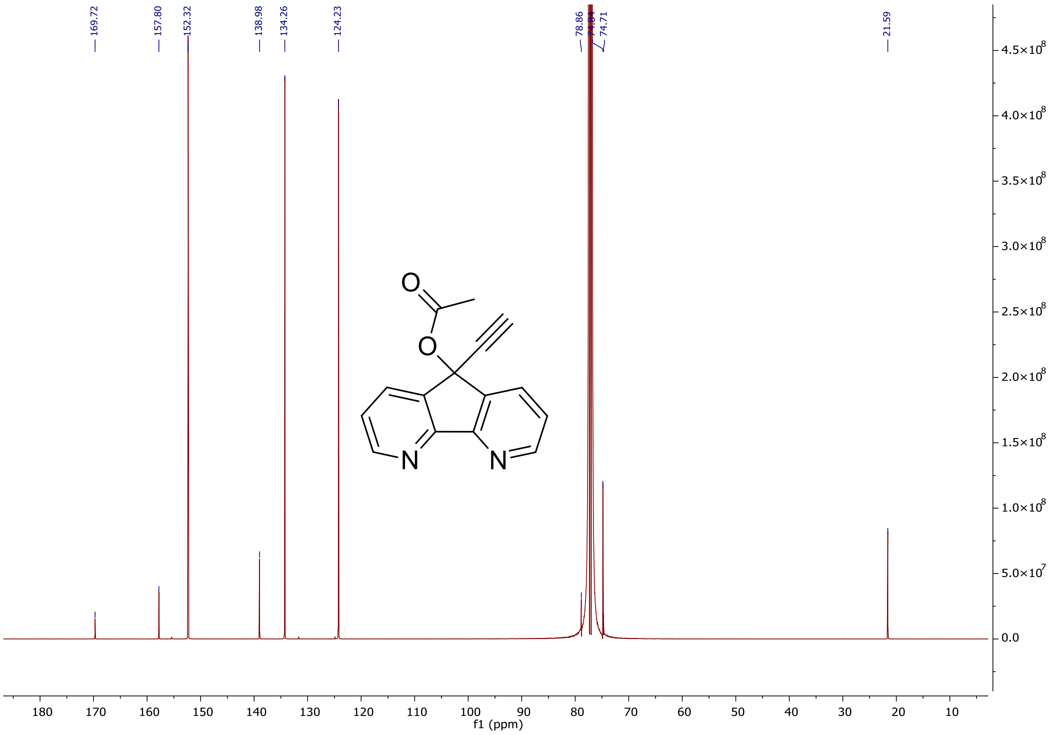
169.72
157.80
152.32
138.98
134.26
124.23
78.86
74.84
74.71
21.59
O
O
N
N
f1 (ppm)

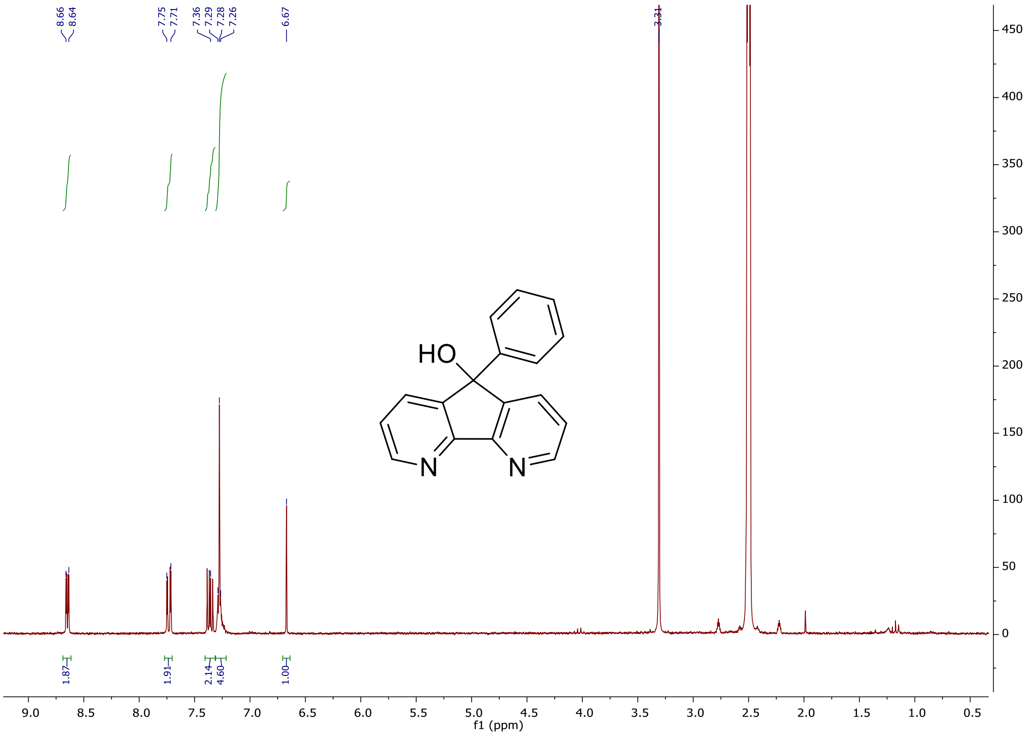
8.66
8.64
7.75
7.71
7.36
7.29
7.28
7.26
6.67
3.31
HO
N
N
1.87
1.91
2.14
4.60
1.00
f1 (ppm)

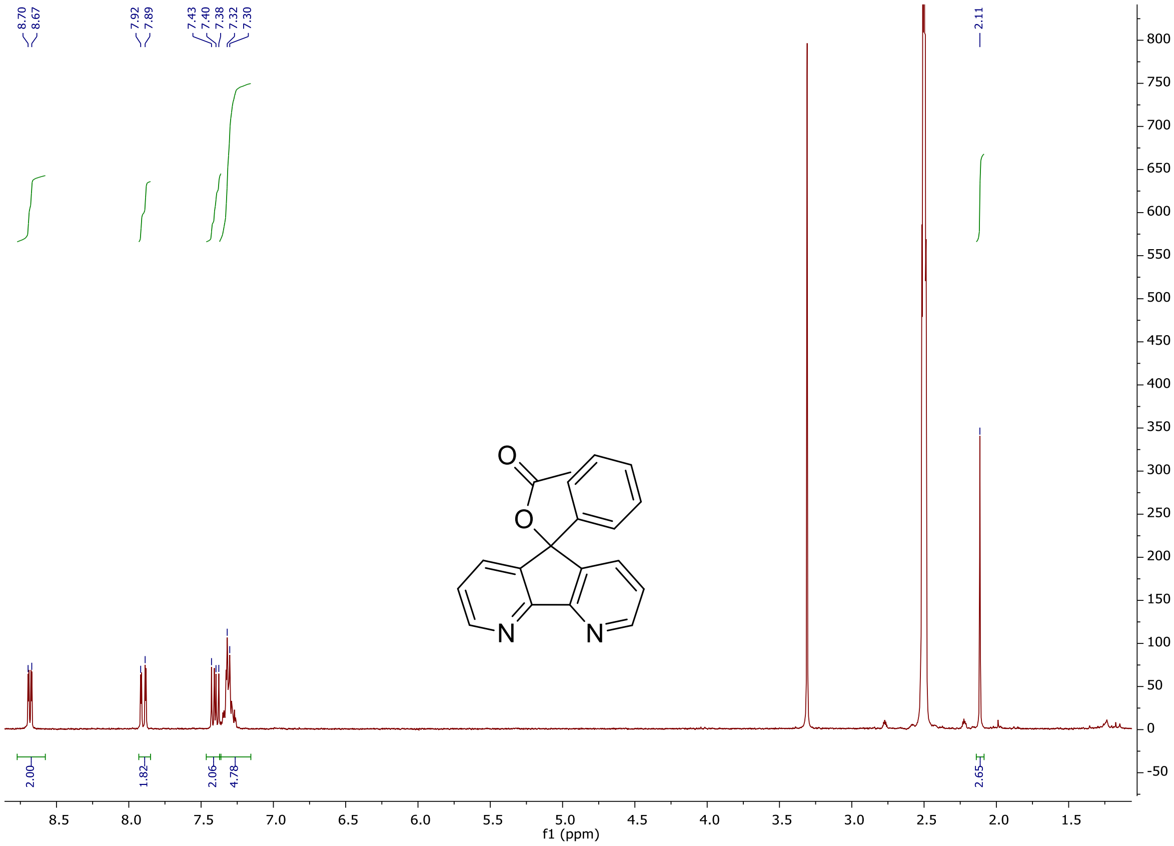
8.70
8.67
7.92
7.89
7.43
7.40
7.38
7.32
7.30
2.11
O
O
N
N
2.00
1.82
2.06
4.78
2.65
f1 (ppm)

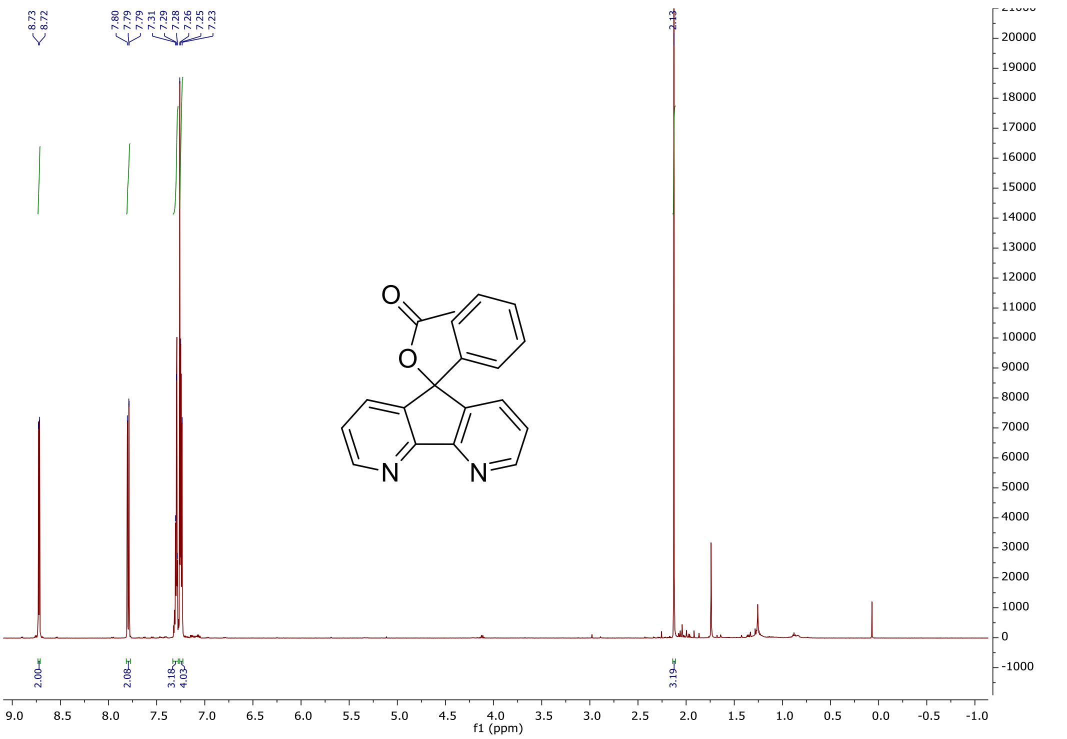
8.73
8.72
7.80
7.79
7.79
7.31
7.29
7.28
7.26
7.25
7.23
2.13
2.00
2.08
3.18
4.03
3.19
O
O
N
N
f1 (ppm)

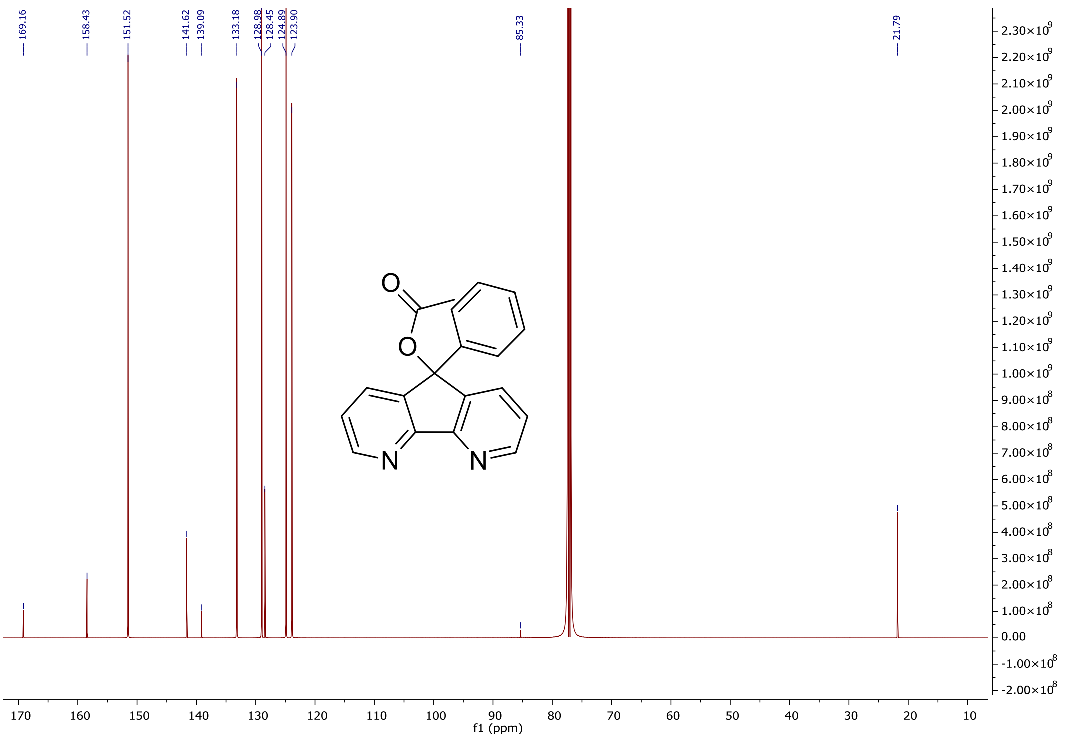
169.16
158.43
151.52
141.62
139.09
133.18
128.98
128.45
124.89
123.90
85.33
21.79
O
O
N
N
f1 (ppm)

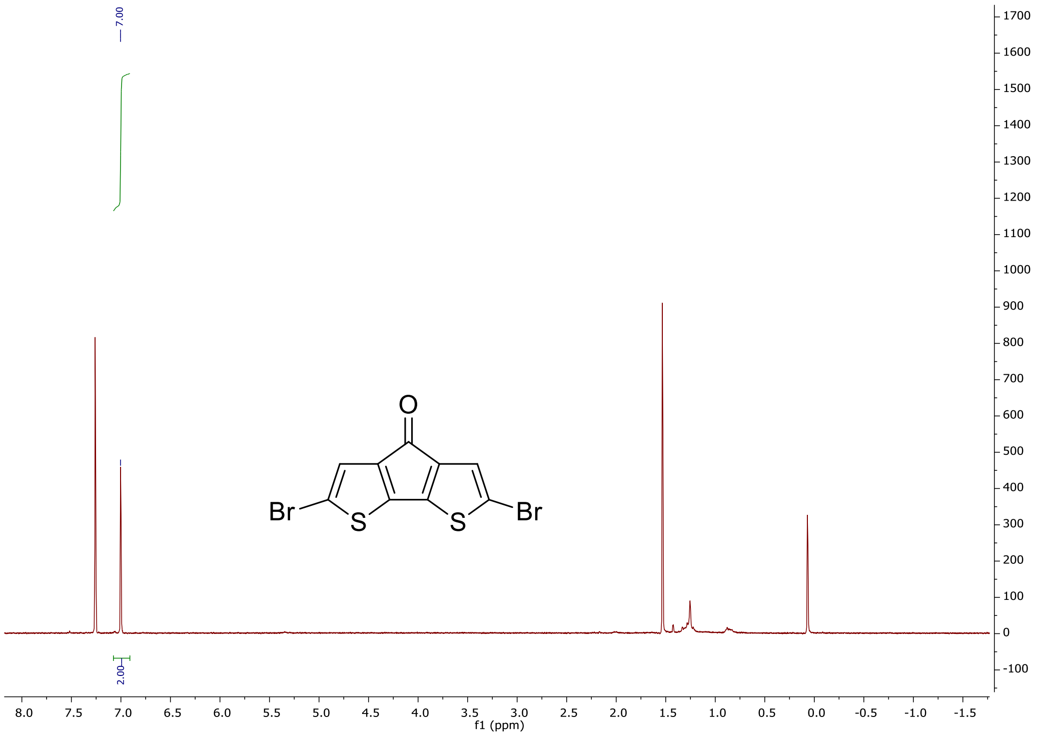
7.00
2.00
O
Br
S
S
Br
f1 (ppm)

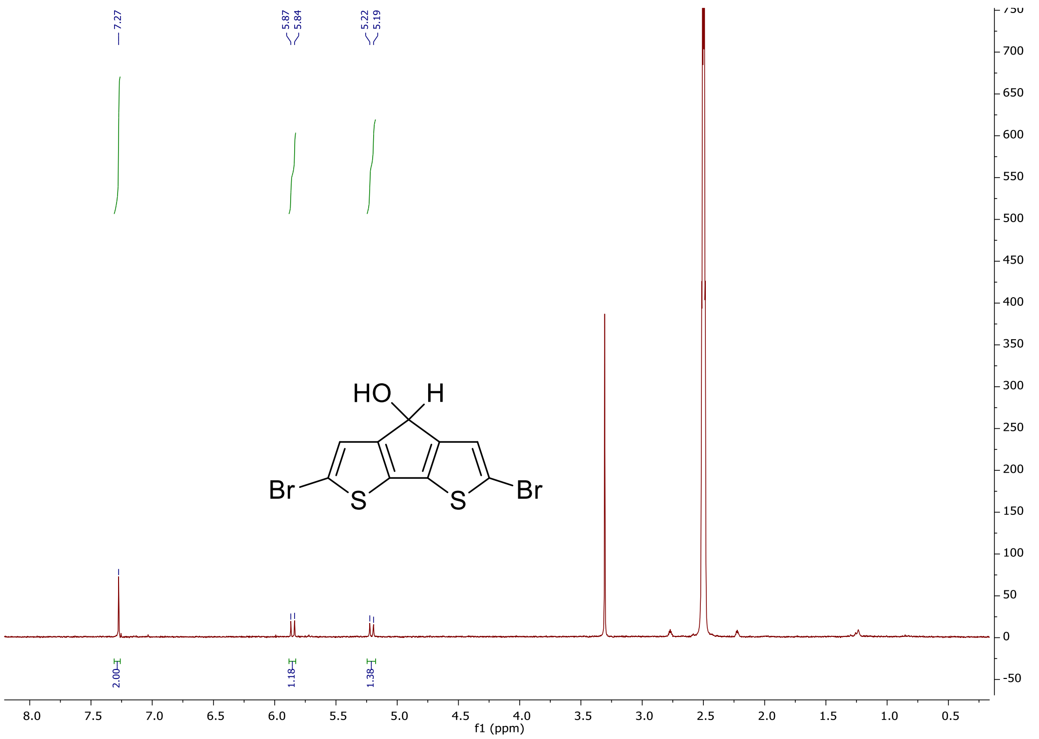
7.27
5.87
5.84
5.22
5.19
2.00
1.18
1.38
HO
H
Br
S
S
Br
f1 (ppm)

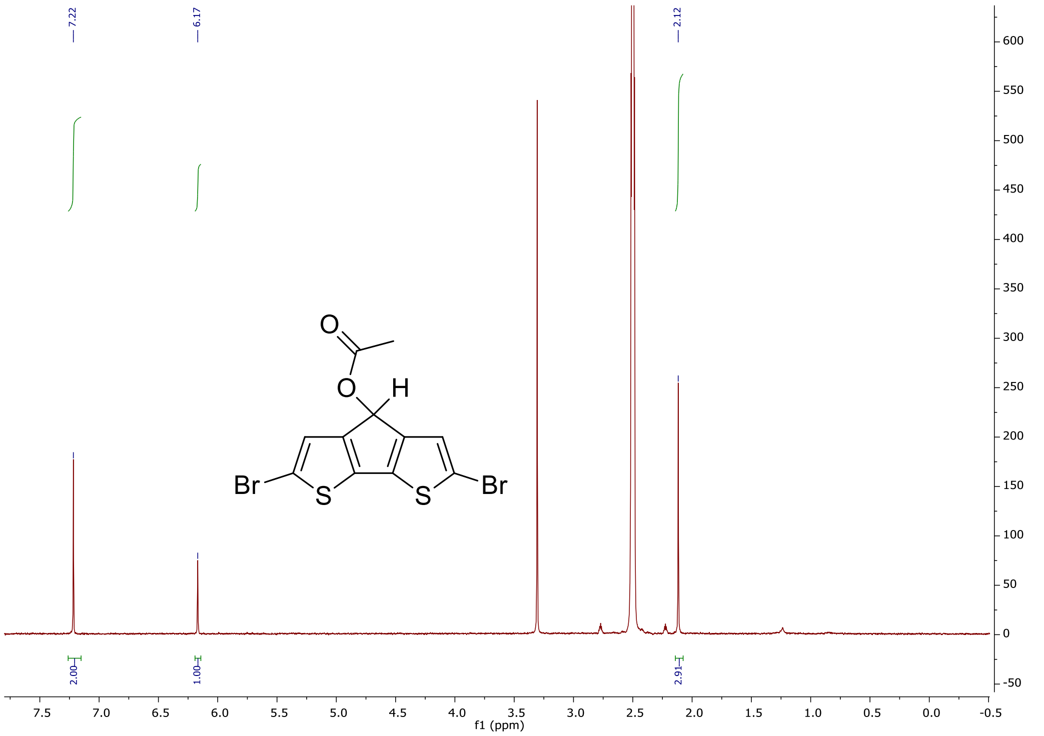
7.22
6.17
2.12
2.00
1.00
2.91
O
O
H
Br
S
S
Br
f1 (ppm)

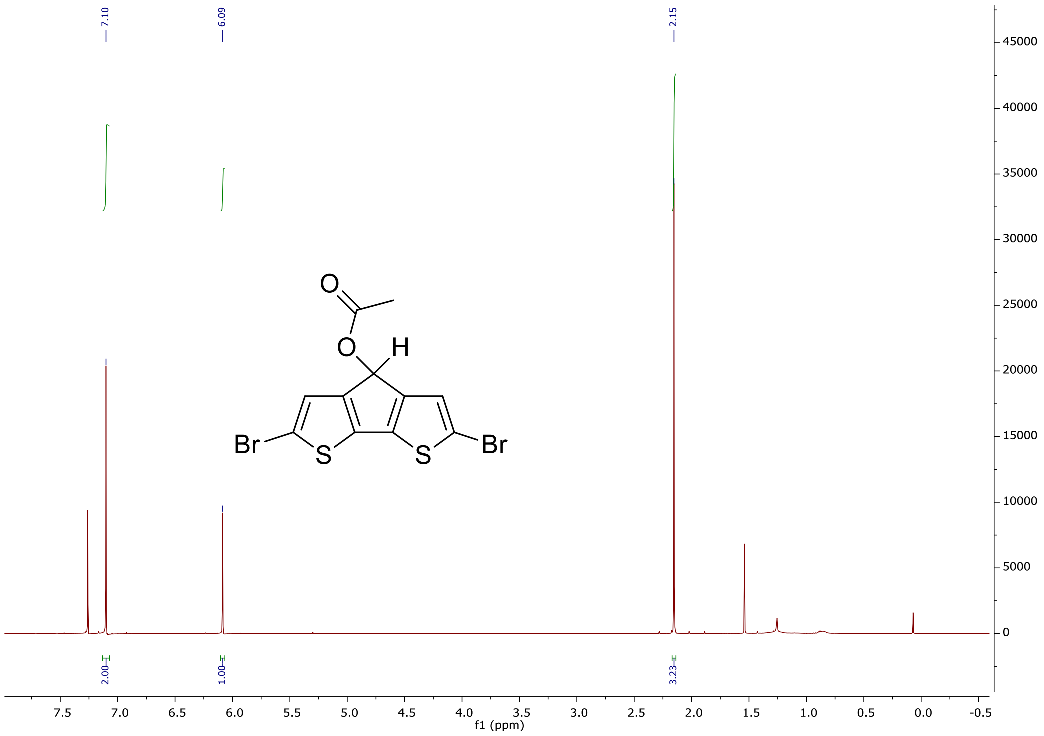
7.10
6.09
2.15
2.00
1.00
3.23
O
O
H
Br
S
S
Br
f1 (ppm)

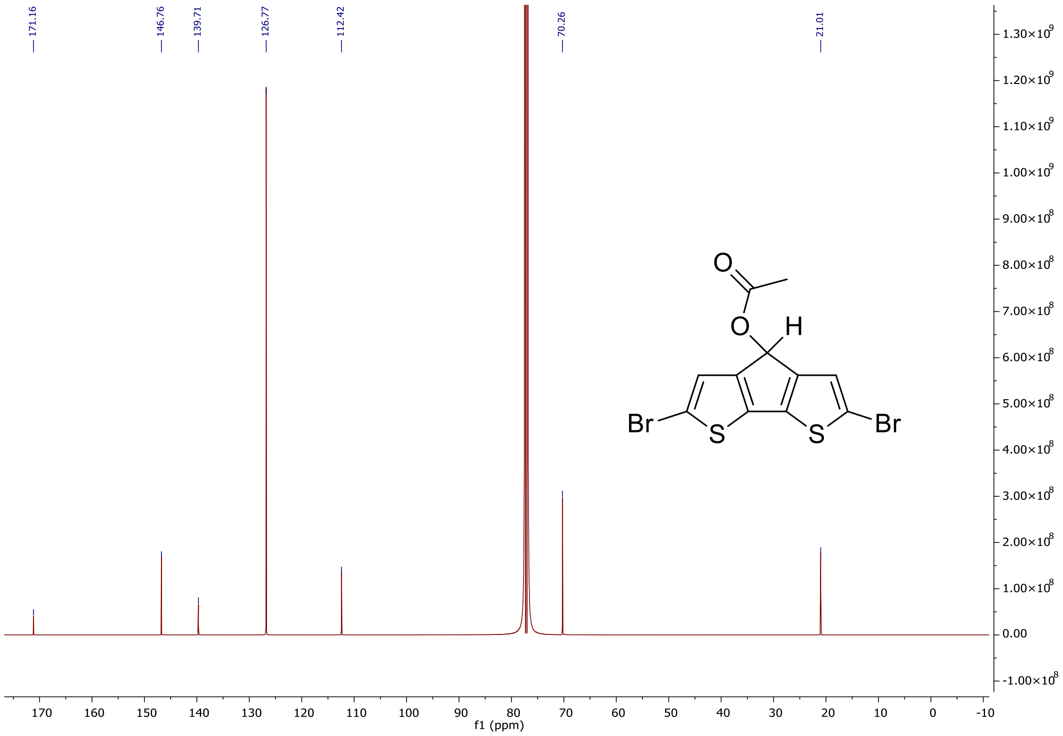
171.16
146.76
139.71
126.77
112.42
70.26
21.01
O
O
H
Br
S
S
Br
f1 (ppm)

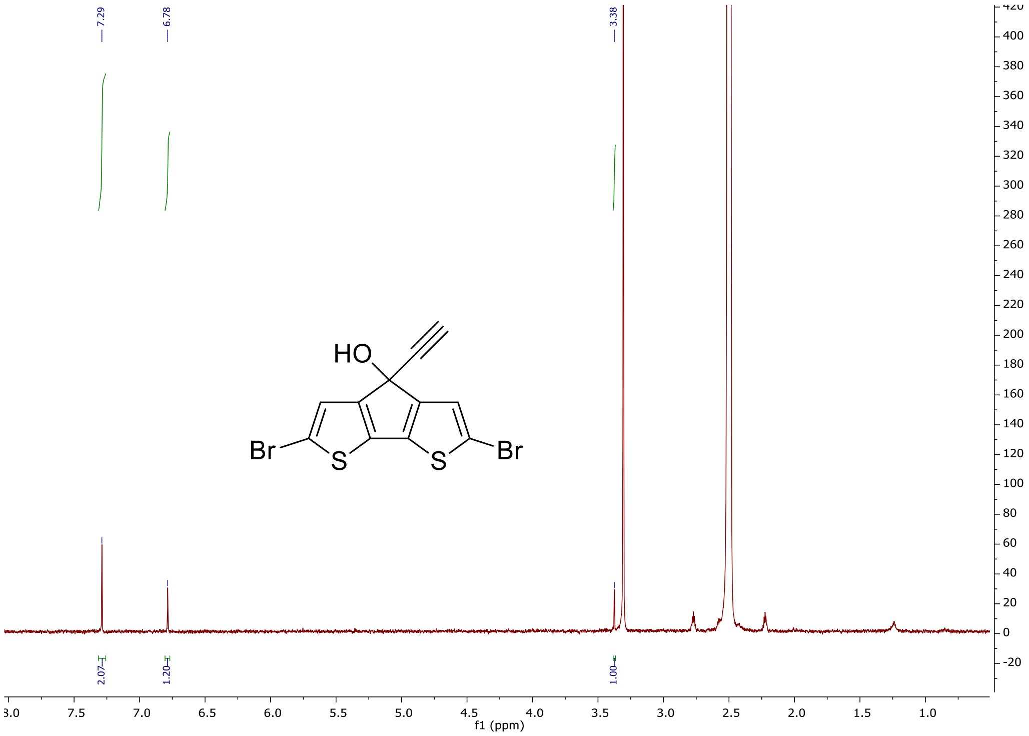
7.29
6.78
3.38
HO
Br
S
S
Br
2.07
1.20
1.00
f1 (ppm)

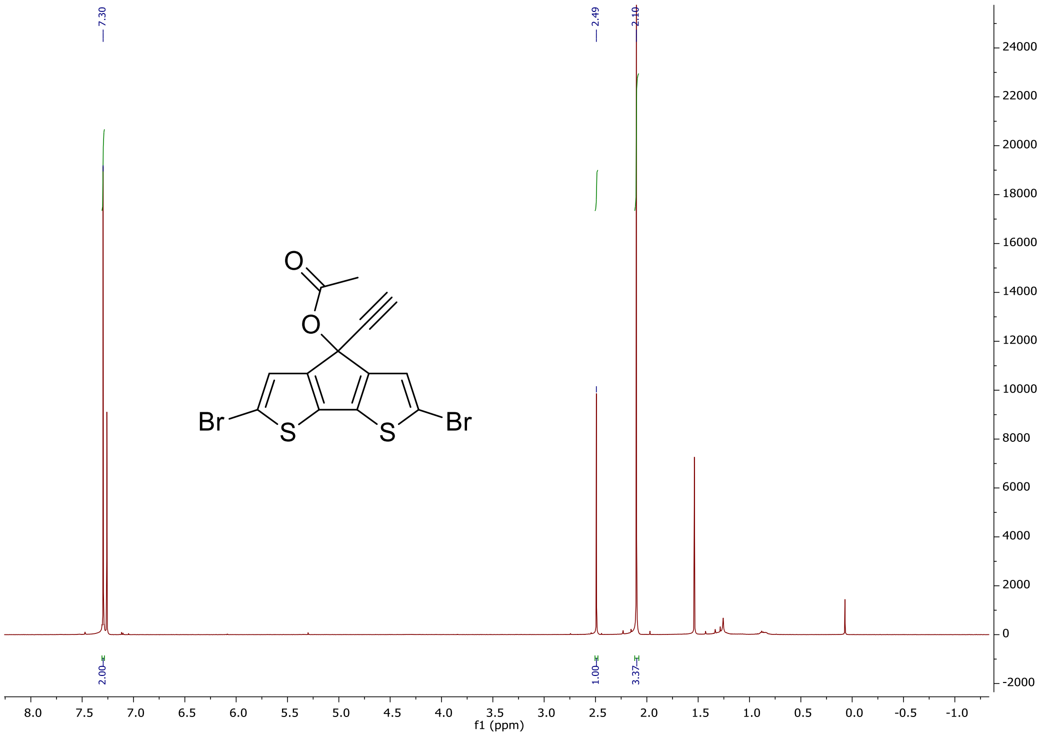
7.30
2.49
2.10
2.00
1.00
3.37
f1 (ppm)
O
O
Br
S
S
Br

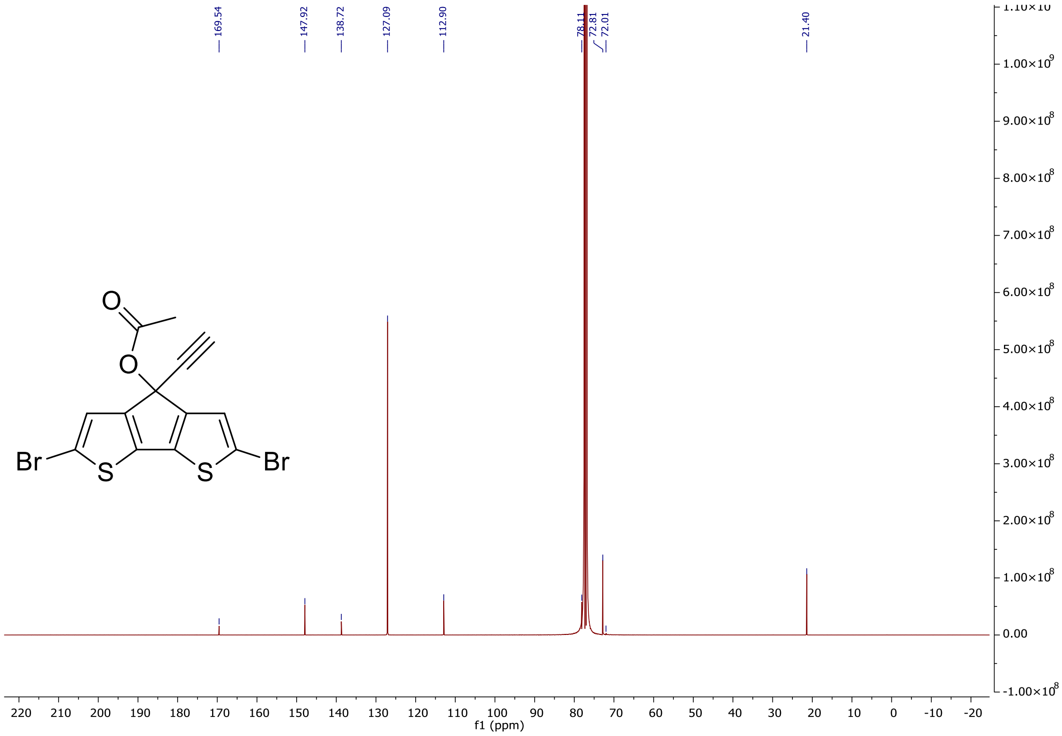
169.54
147.92
138.72
127.09
112.90
78.11
72.81
72.01
21.40
f1 (ppm)
O
O
Br
S
S
Br

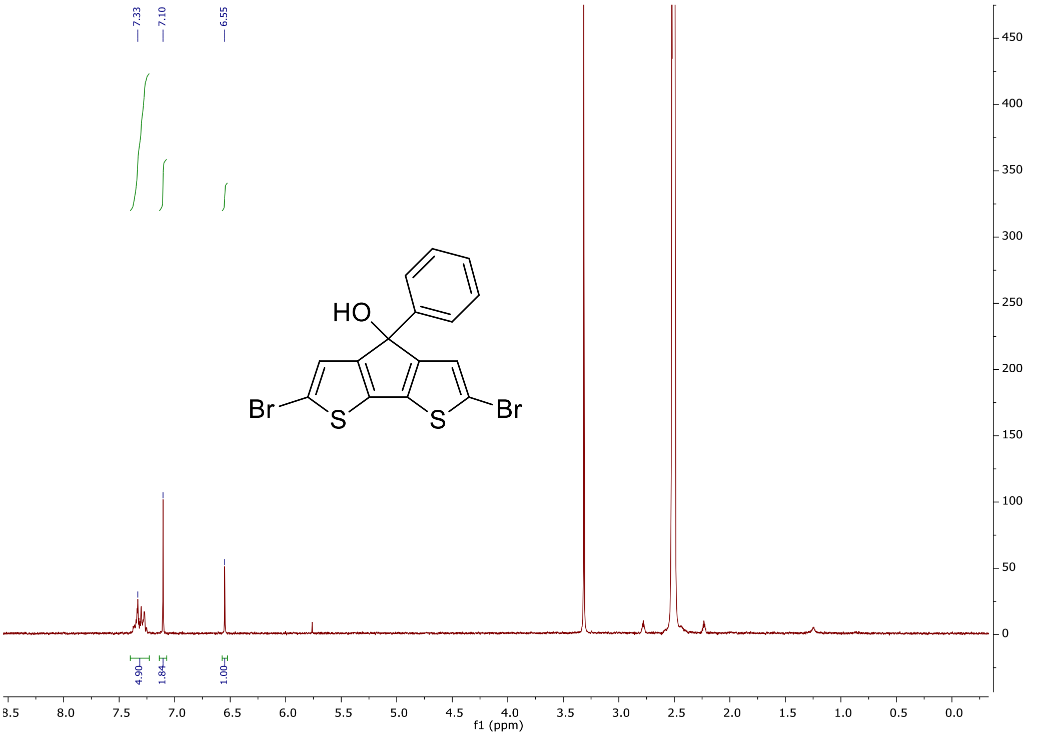

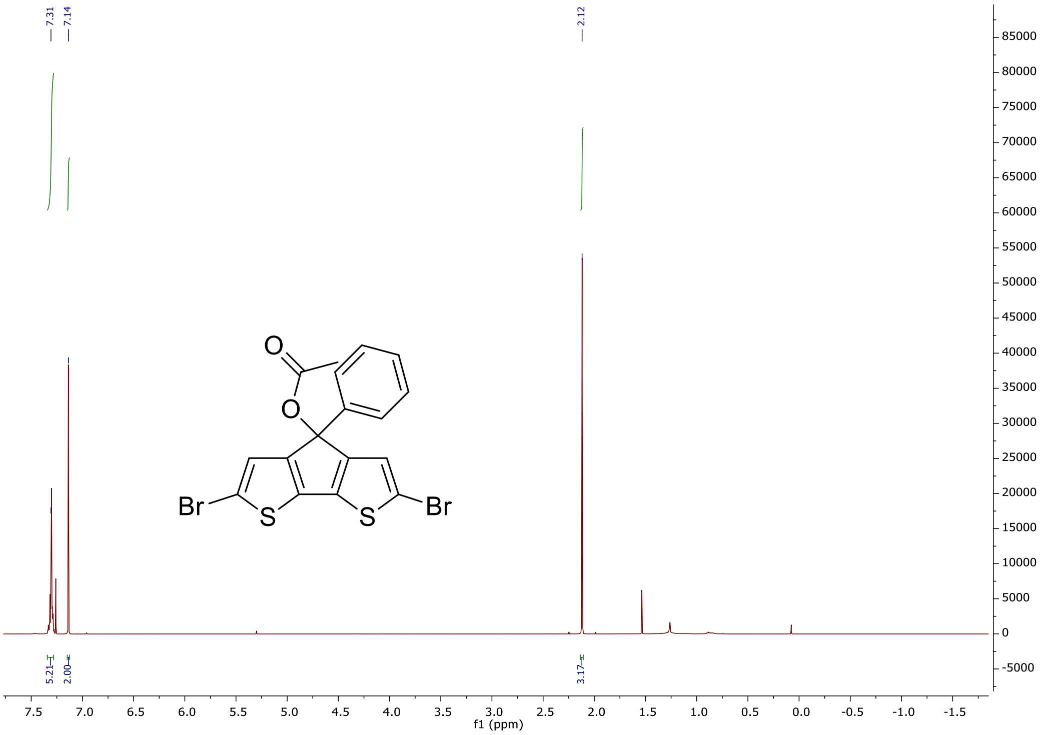

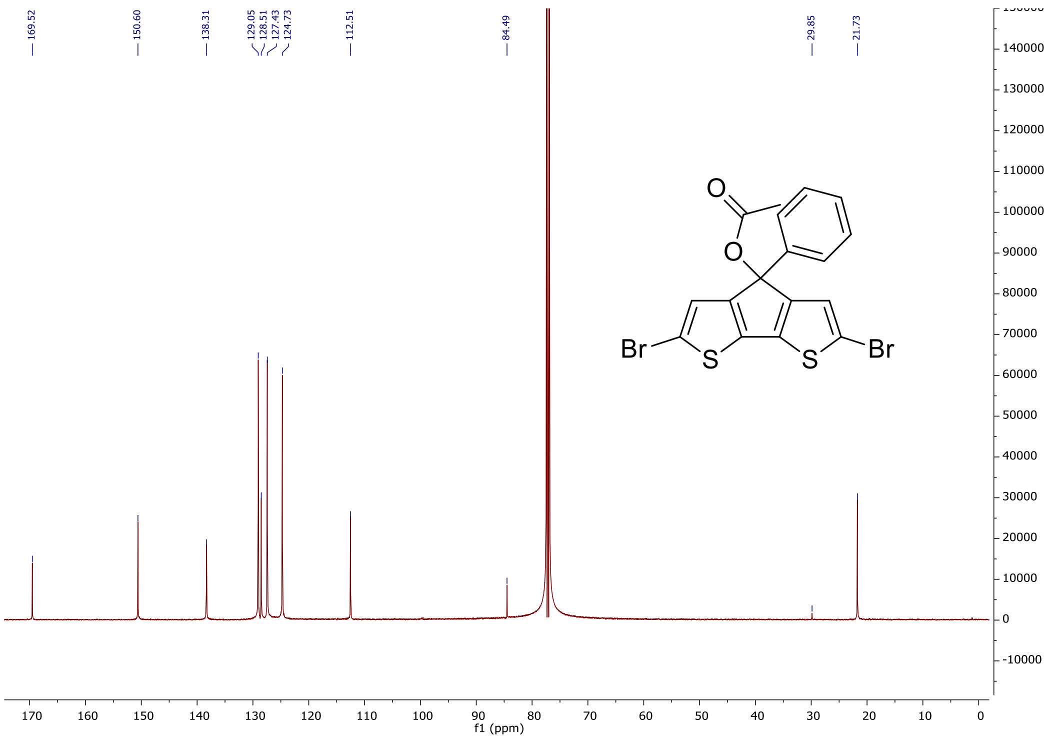

169.52
150.60
138.31
129.05
128.51
127.43
124.73
112.51
84.49
29.85
21.73
Br
S
S
Br
O
O
f1 (ppm)

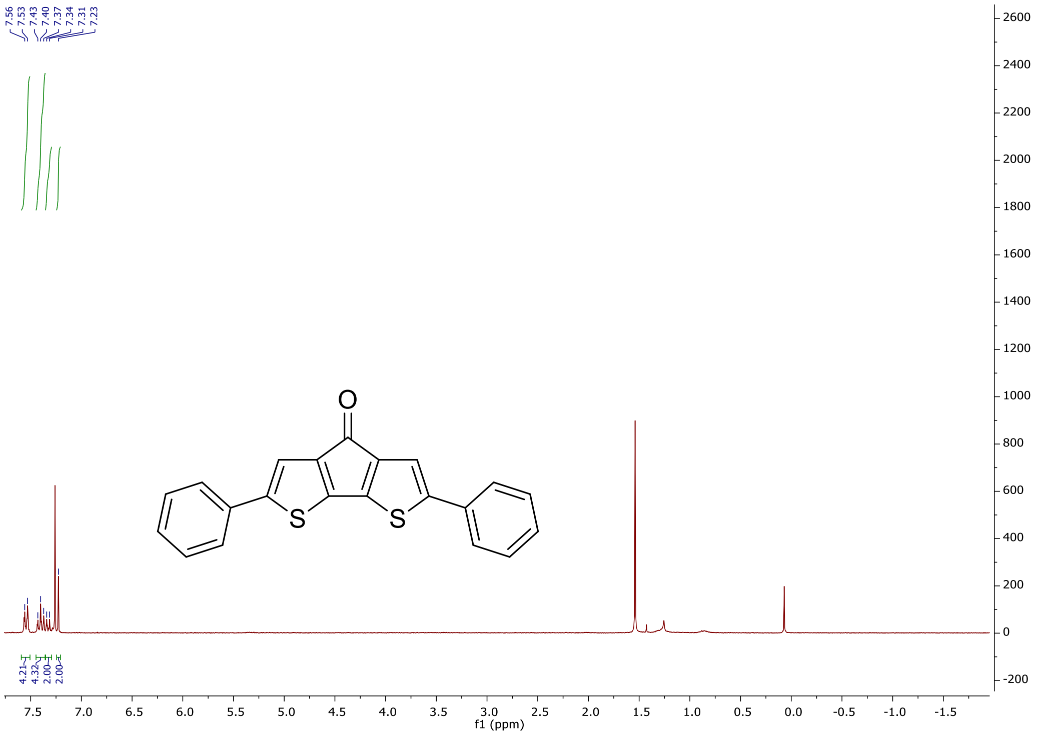

7.56
7.53
7.43
7.40
7.37
7.34
7.31
7.23
O
S
S
4.21
4.32
2.00
2.00
f1 (ppm)

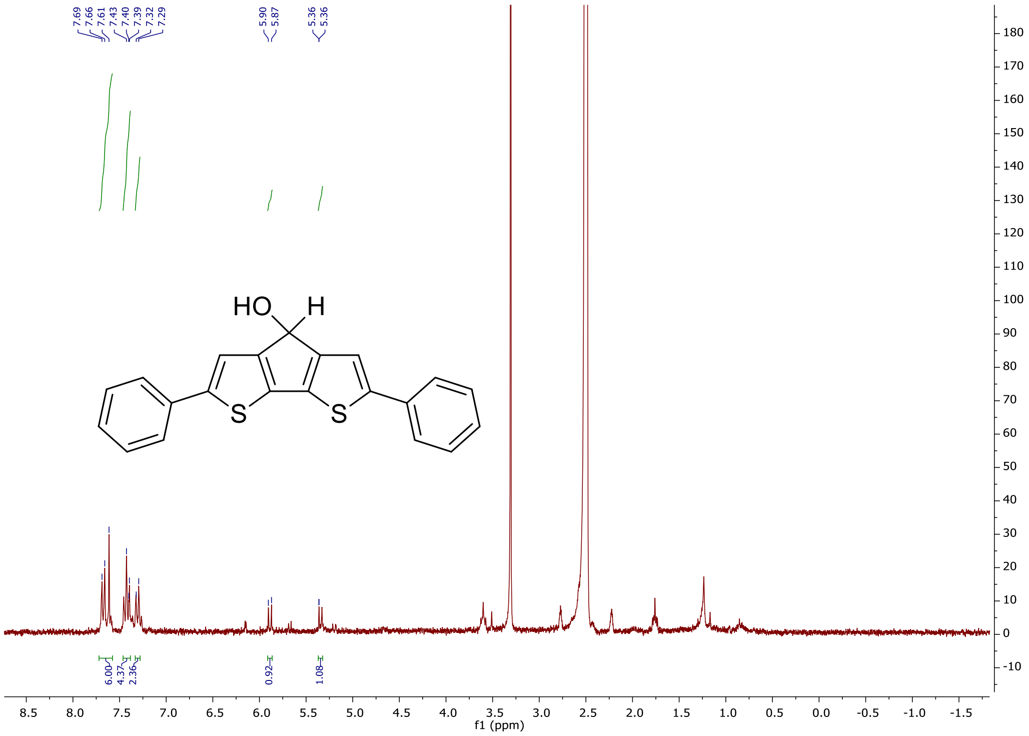
HO
H
S
S
f1 (ppm)

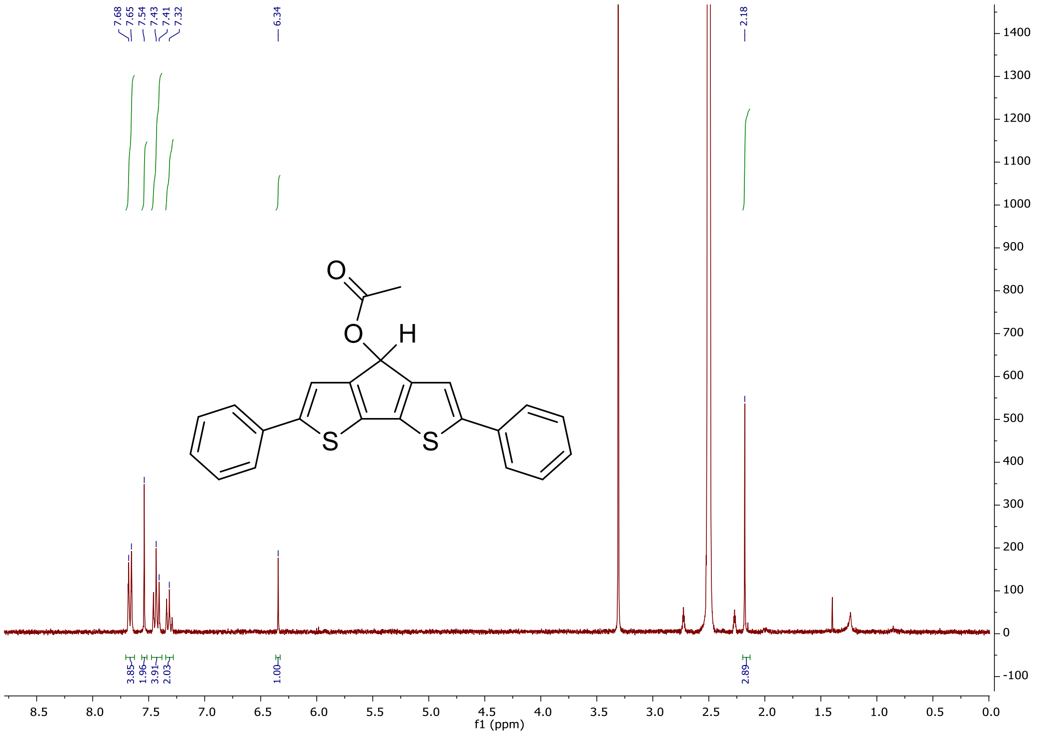
O
O
H
S
S
f1 (ppm)

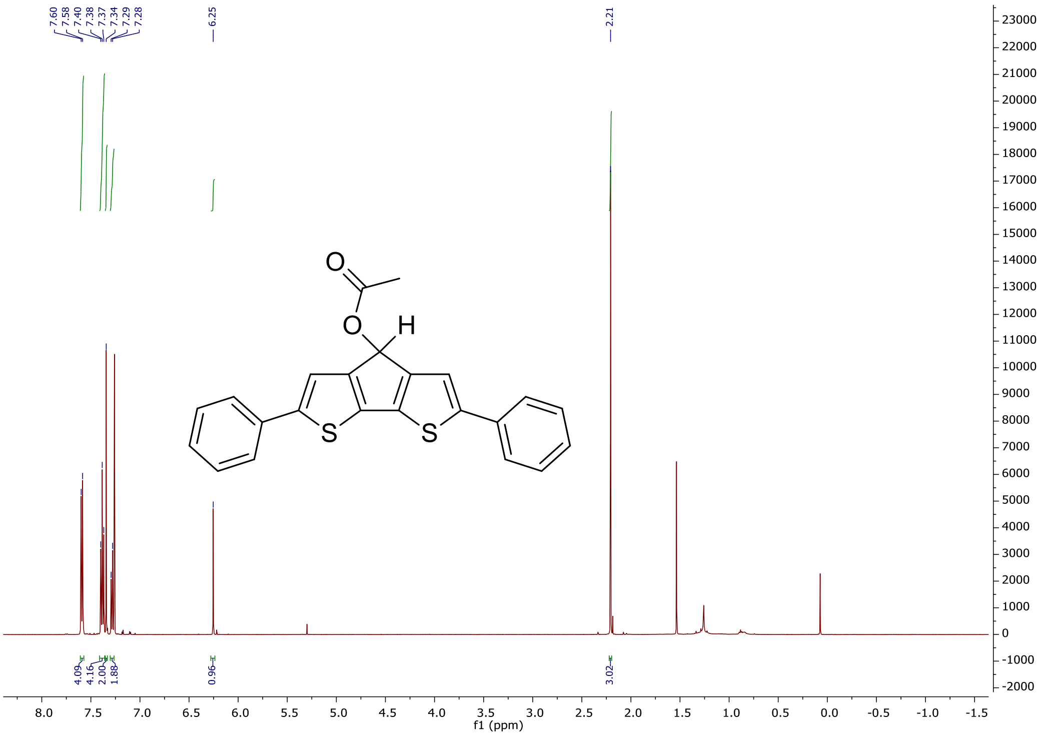
7.60
7.58
7.40
7.38
7.37
7.34
7.29
7.28
6.25
2.21
4.09
4.16
2.00
1.88
0.96
3.02
f1 (ppm)
O
O
H
S
S

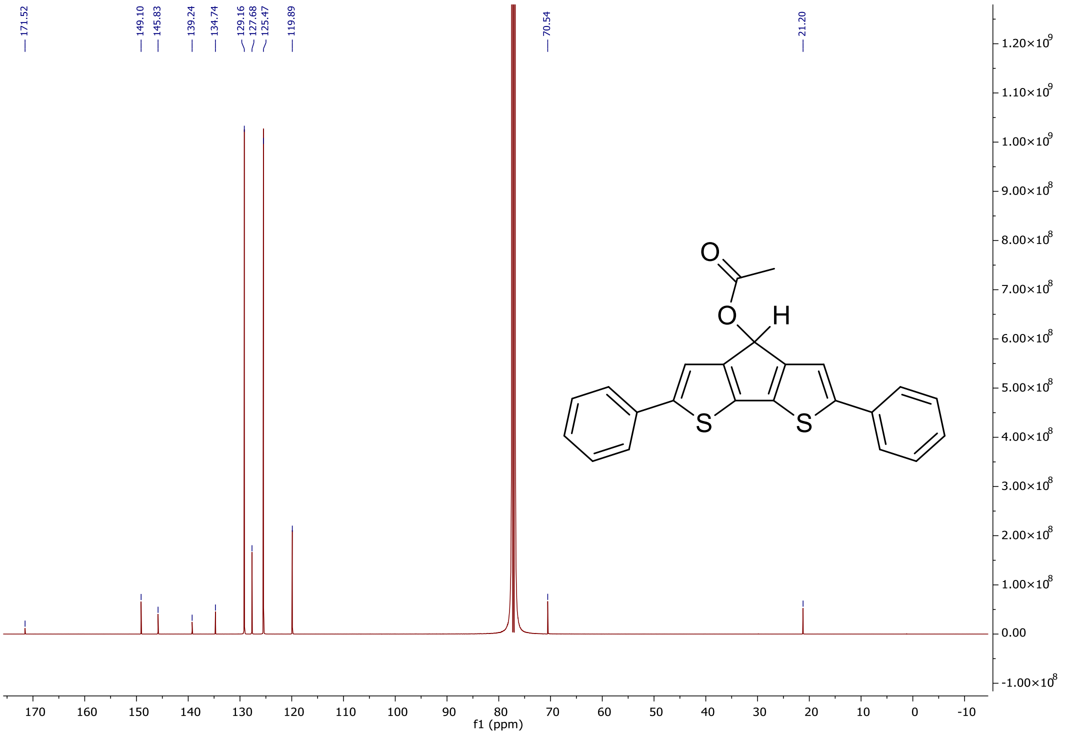
171.52
149.10
145.83
139.24
134.74
129.16
127.68
125.47
119.89
70.54
21.20
f1 (ppm)
O
O
H
S
S

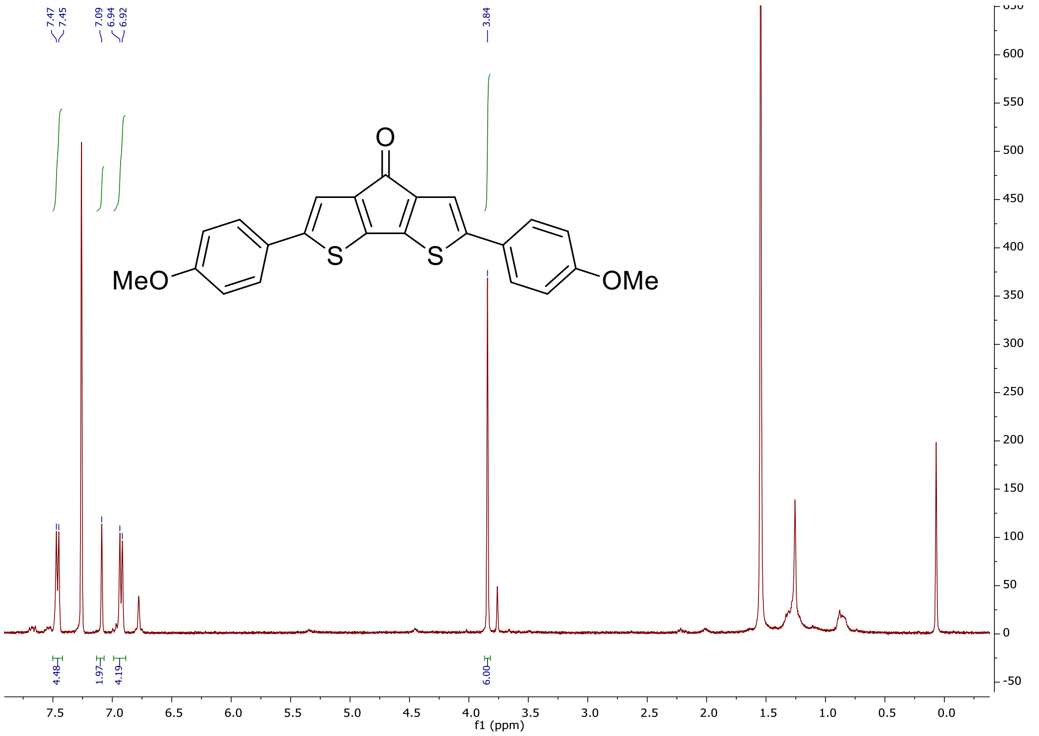
7.47
7.45
7.09
6.94
6.92
3.84
O
S
S
MeO
OMe
4.48
1.97
4.19
6.00
f1 (ppm)

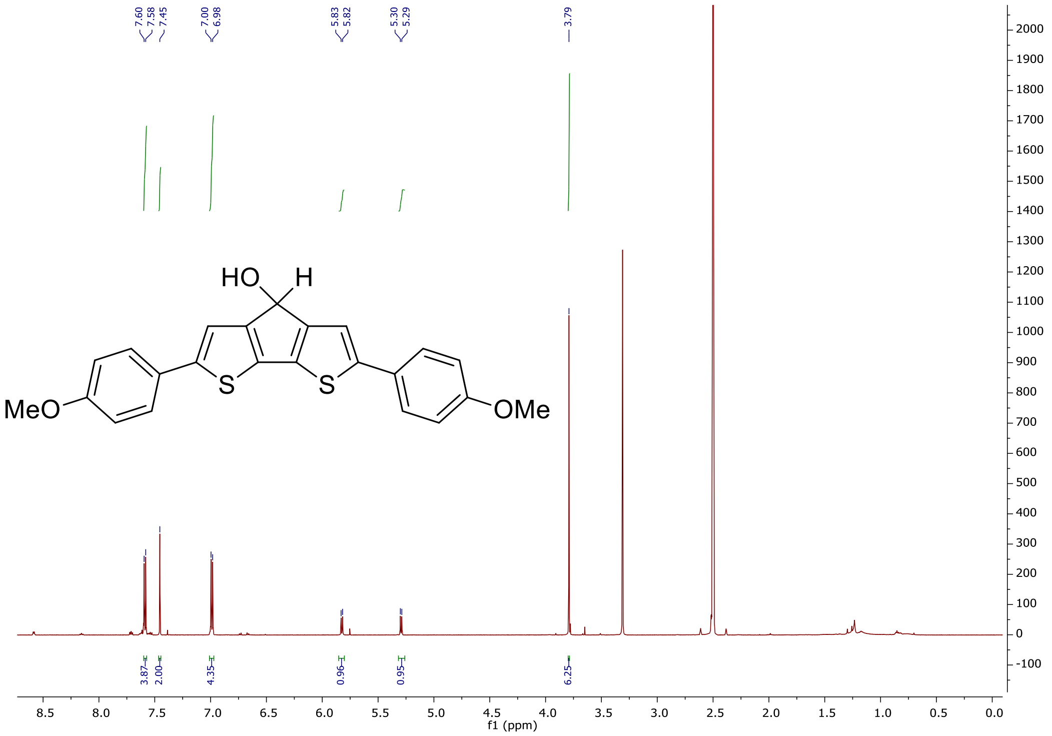
7.60
7.58
7.45
7.00
6.98
5.83
5.82
5.30
5.29
3.79
HO
H
S
S
MeO
OMe
3.87
2.00
4.35
0.96
0.95
6.25
f1 (ppm)

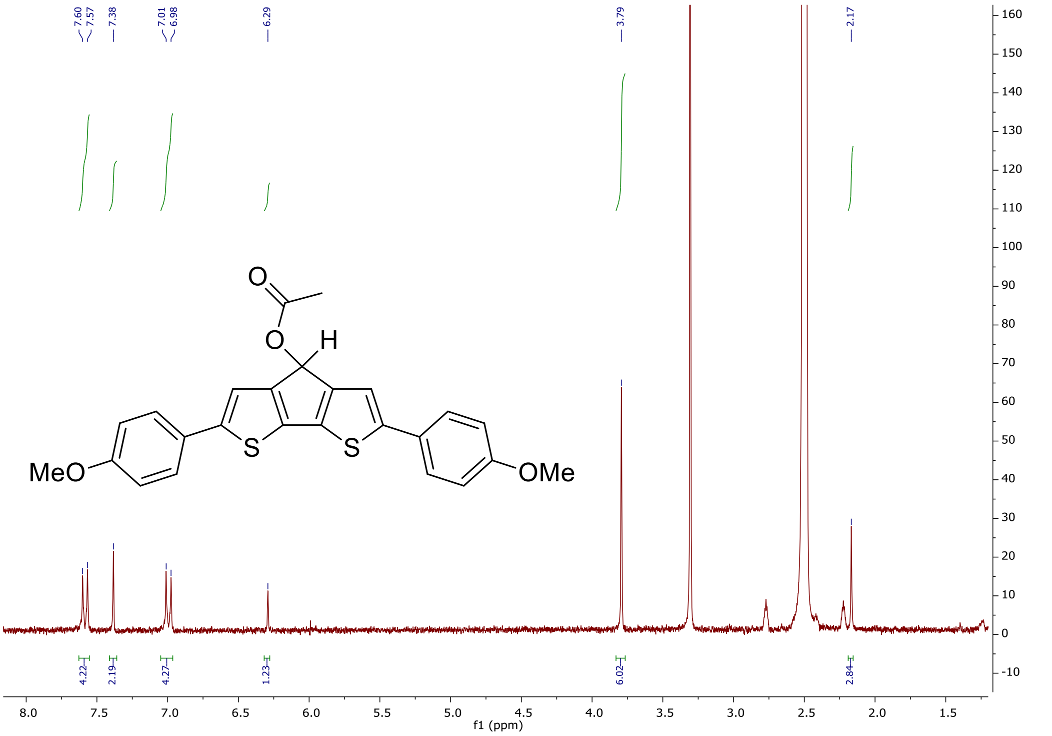

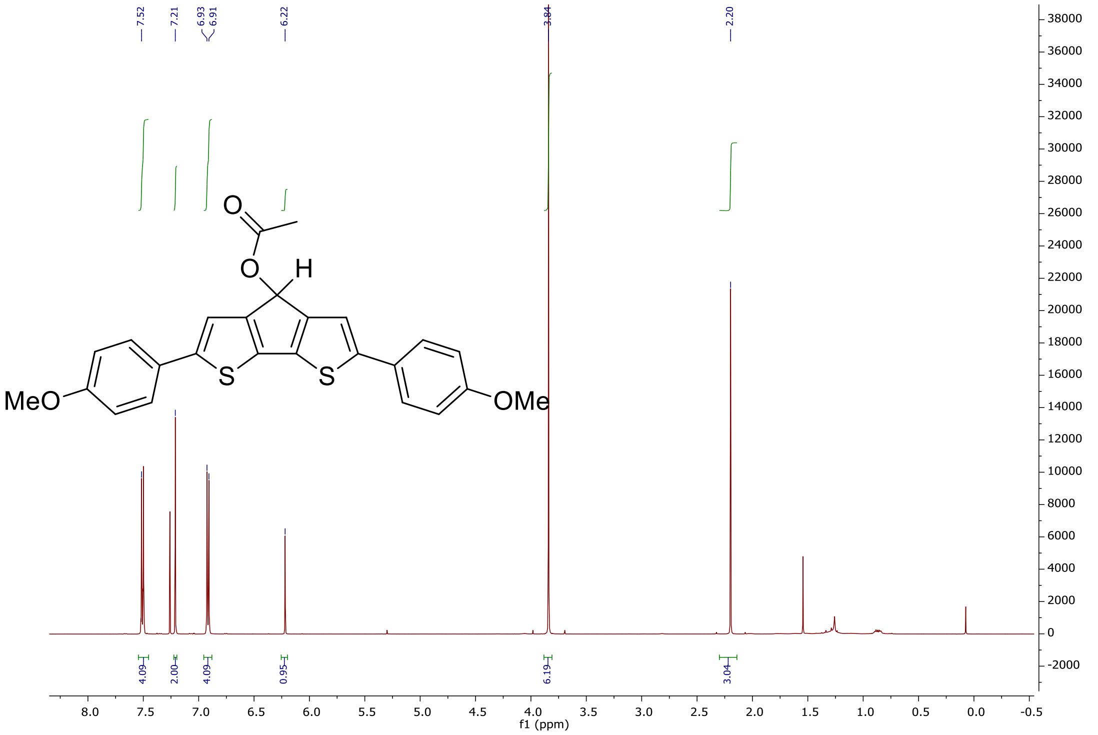

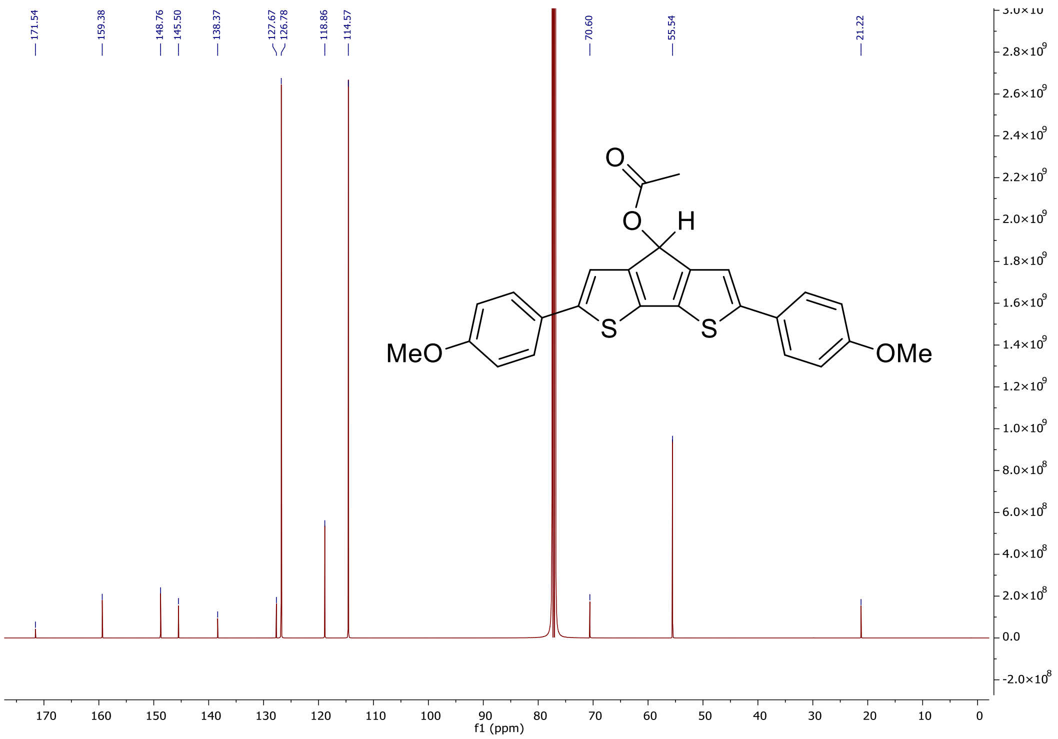
171.54
159.38
148.76
145.50
138.37
127.67
126.78
118.86
114.57
70.60
55.54
21.22
O
O
H
S
S
MeO
OMe
f1 (ppm)

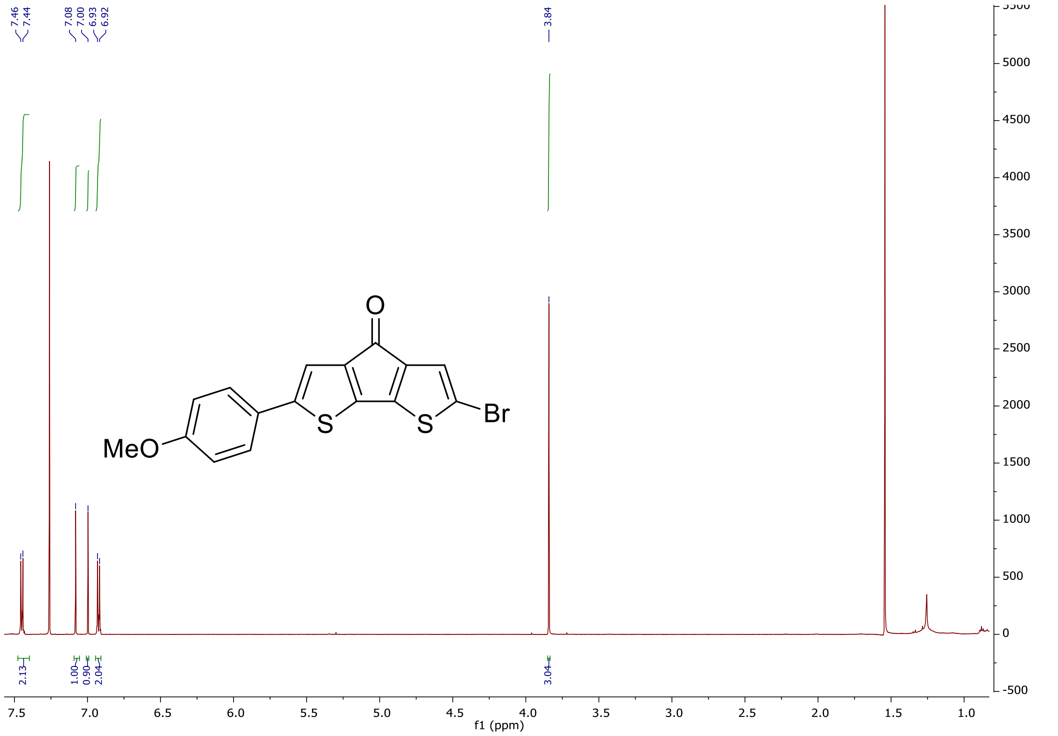
7.46
7.44
7.08
7.00
6.93
6.92
3.84
O
S
S
Br
MeO
2.13
1.00
0.90
2.04
3.04
f1 (ppm)

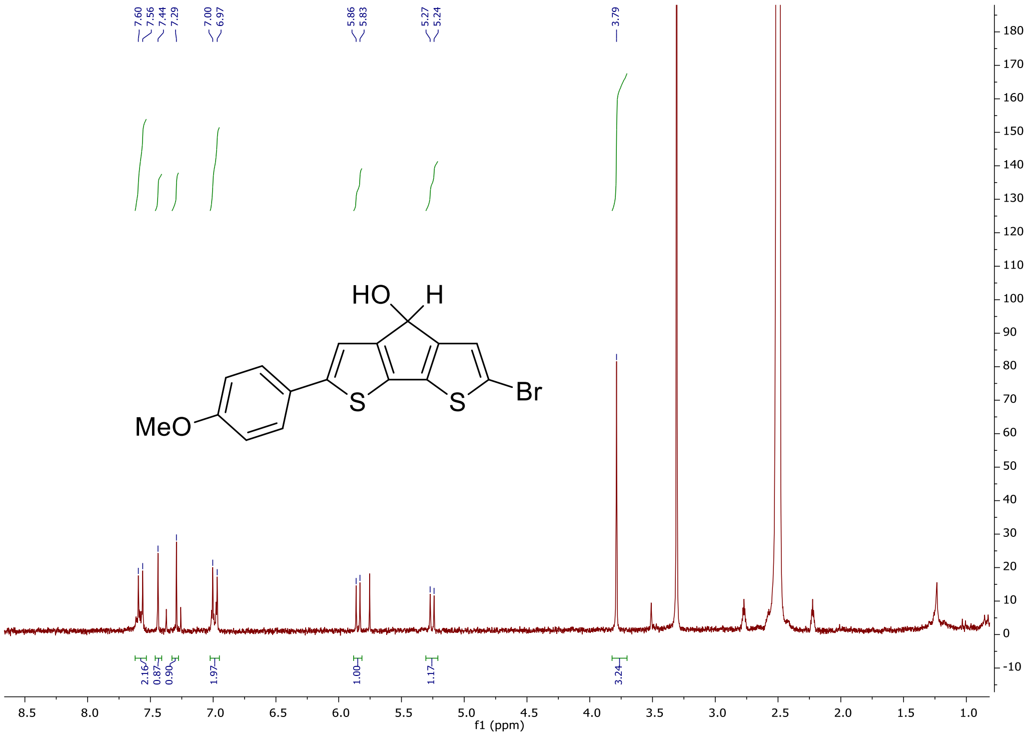

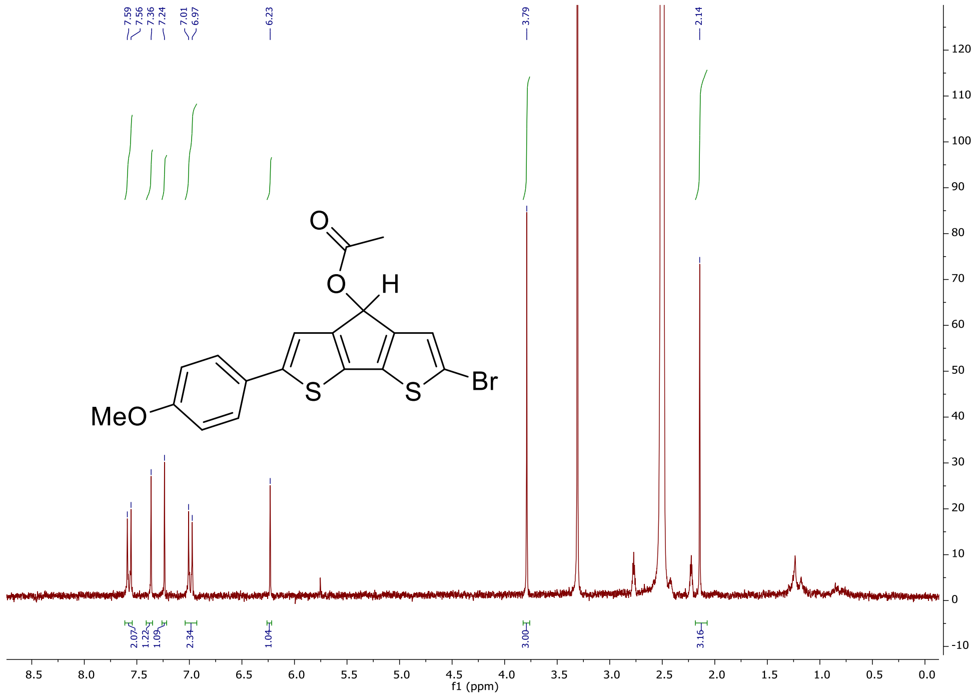

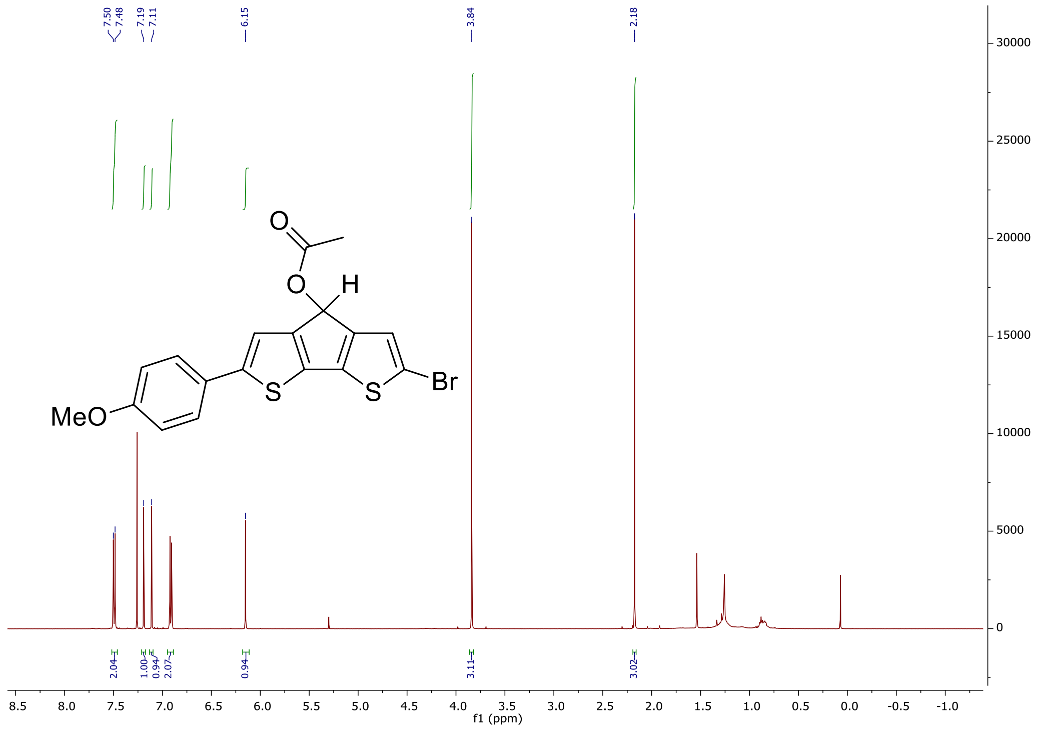
7.50
7.48
7.19
7.11
6.15
3.84
2.18
MeO
O
O
H
S
S
Br
2.04
1.00
0.94
2.07
0.94
3.11
3.02
30000
25000
20000
15000
10000
5000
0
8.5 8.0 7.5 7.0 6.5 6.0 5.5 5.0 4.5 4.0 3.5 3.0 2.5 2.0 1.5 1.0 0.5 0.0 -0.5 -1.0
f1 (ppm)

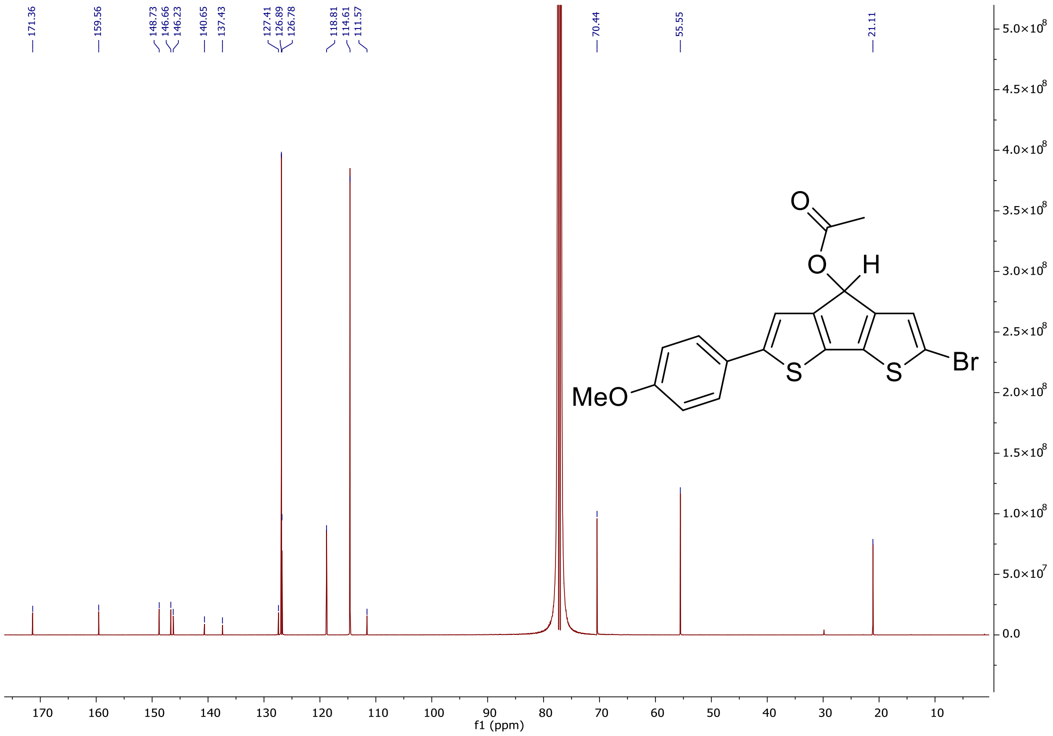
171.36
159.56
148.73
146.66
146.23
140.65
137.43
127.41
126.89
126.78
118.81
114.61
111.57
70.44
55.55
21.11
MeO
O
O
H
S
S
Br
5.0×10^8
4.5×10^8
4.0×10^8
3.5×10^8
3.0×10^8
2.5×10^8
2.0×10^8
1.5×10^8
1.0×10^8
5.0×10^7
0.0
170 160 150 140 130 120 110 100 90 80 70 60 50 40 30 20 10
f1 (ppm)

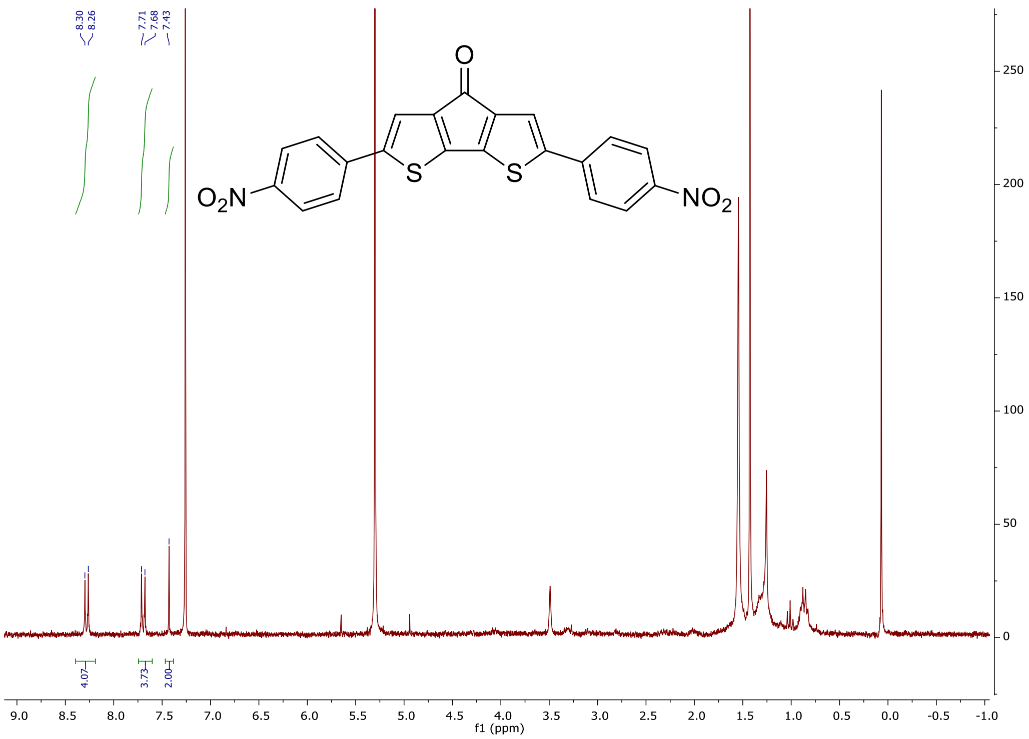
8.30
8.26
7.71
7.68
7.43
O
S
S
O_2N
NO_2
4.07
3.73
2.00
f1 (ppm)

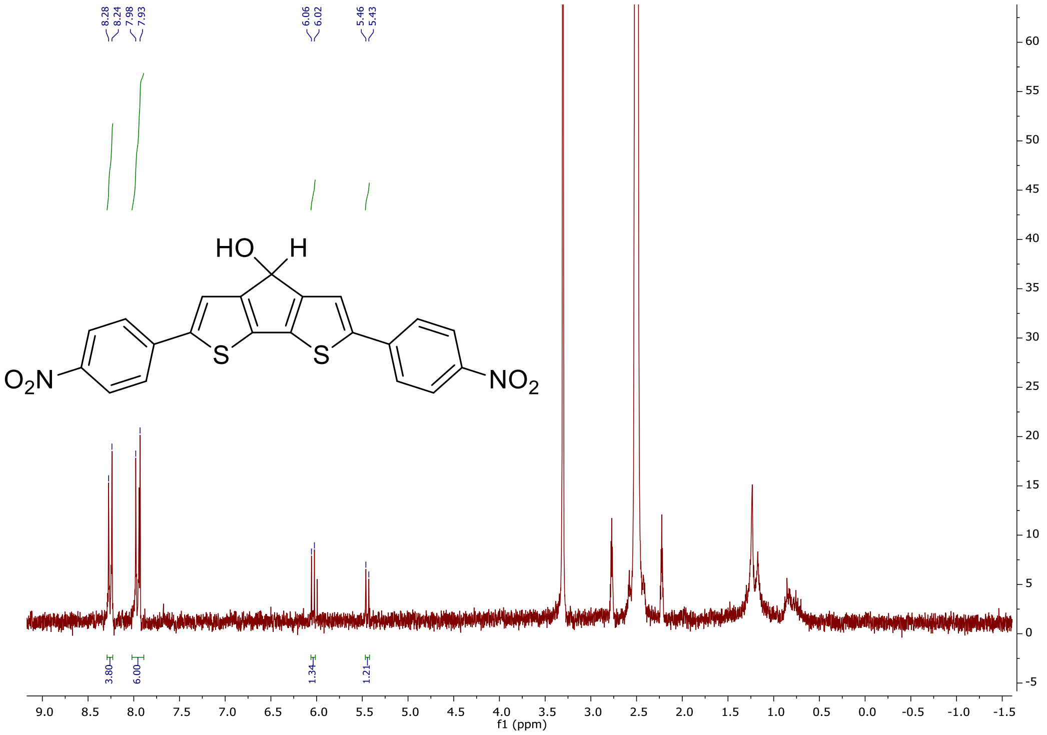
8.28
8.24
7.98
7.93
6.06
6.02
5.46
5.43
HO
H
S
S
O_2N
NO_2
3.80
6.00
1.34
1.21
f1 (ppm)

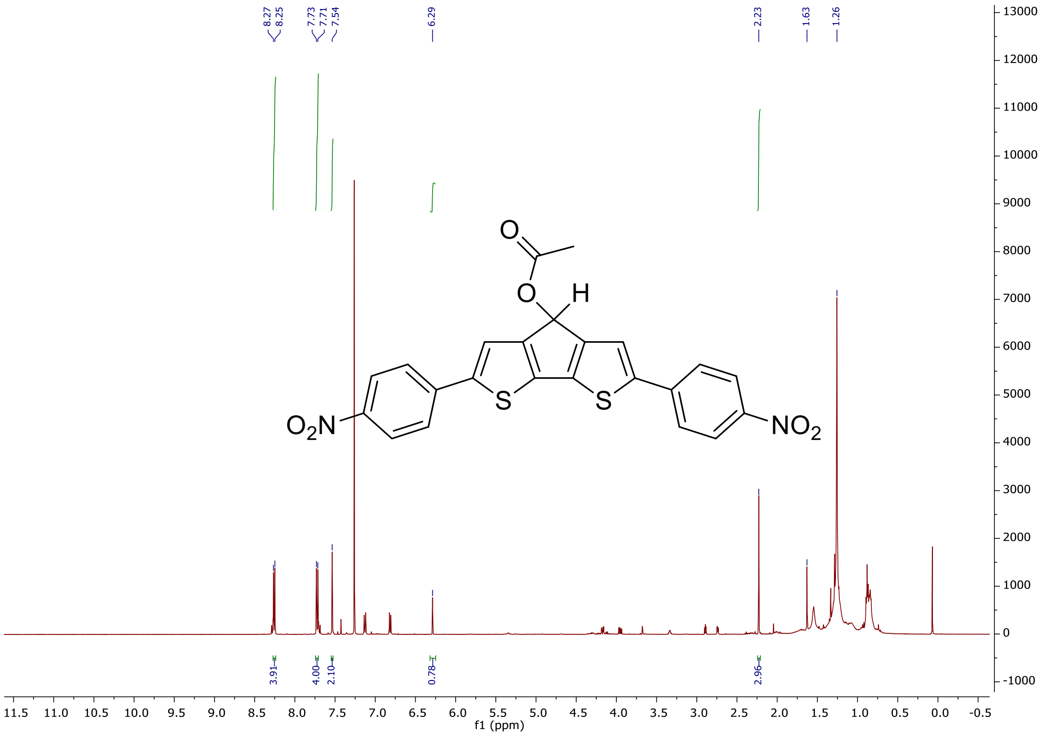

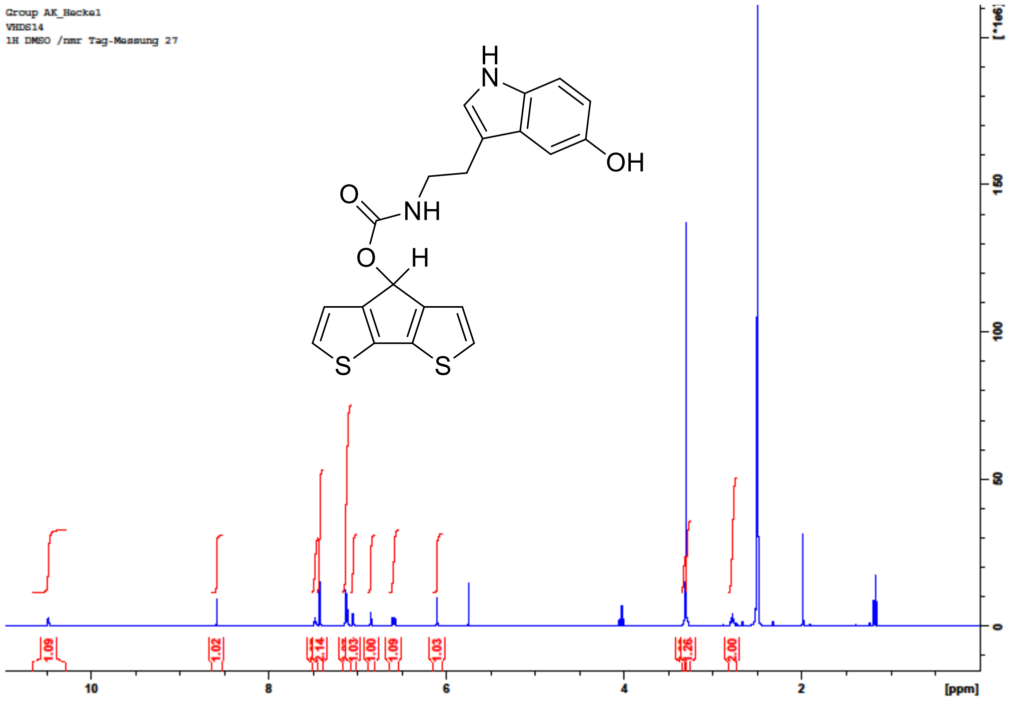
Group AK_Heckel
VHDS14
1H DMSO /nmr Tag-Messung 27

6. Mass spectra of key compounds

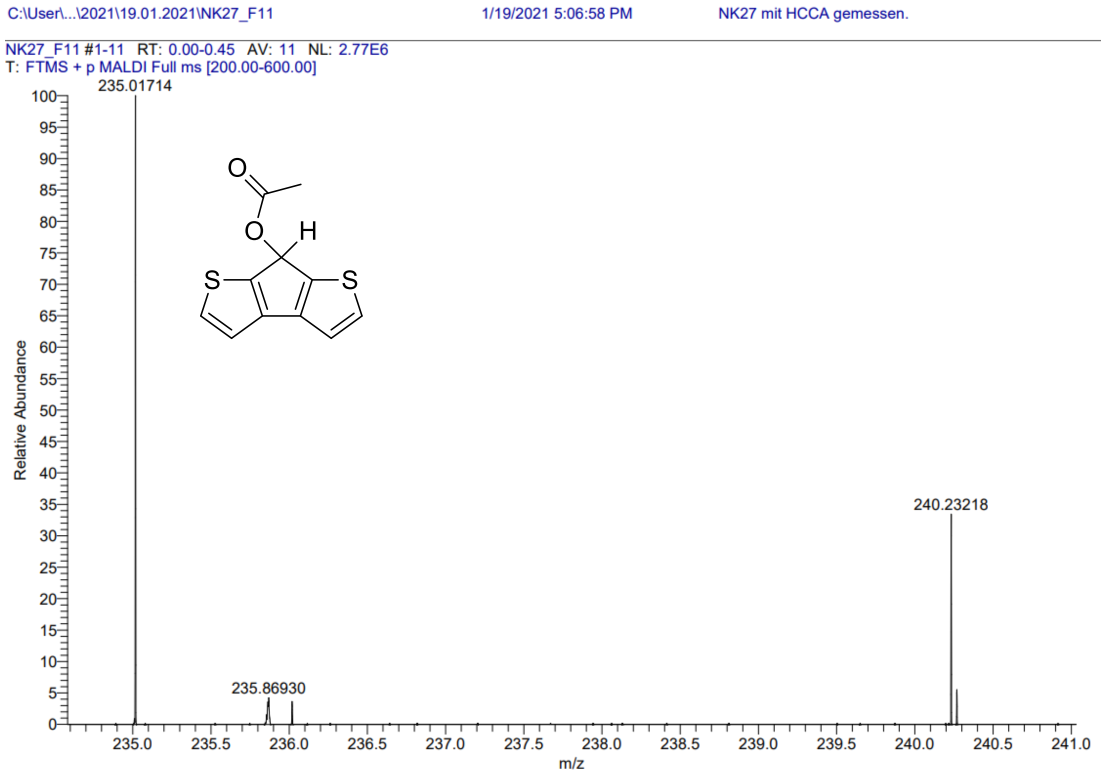

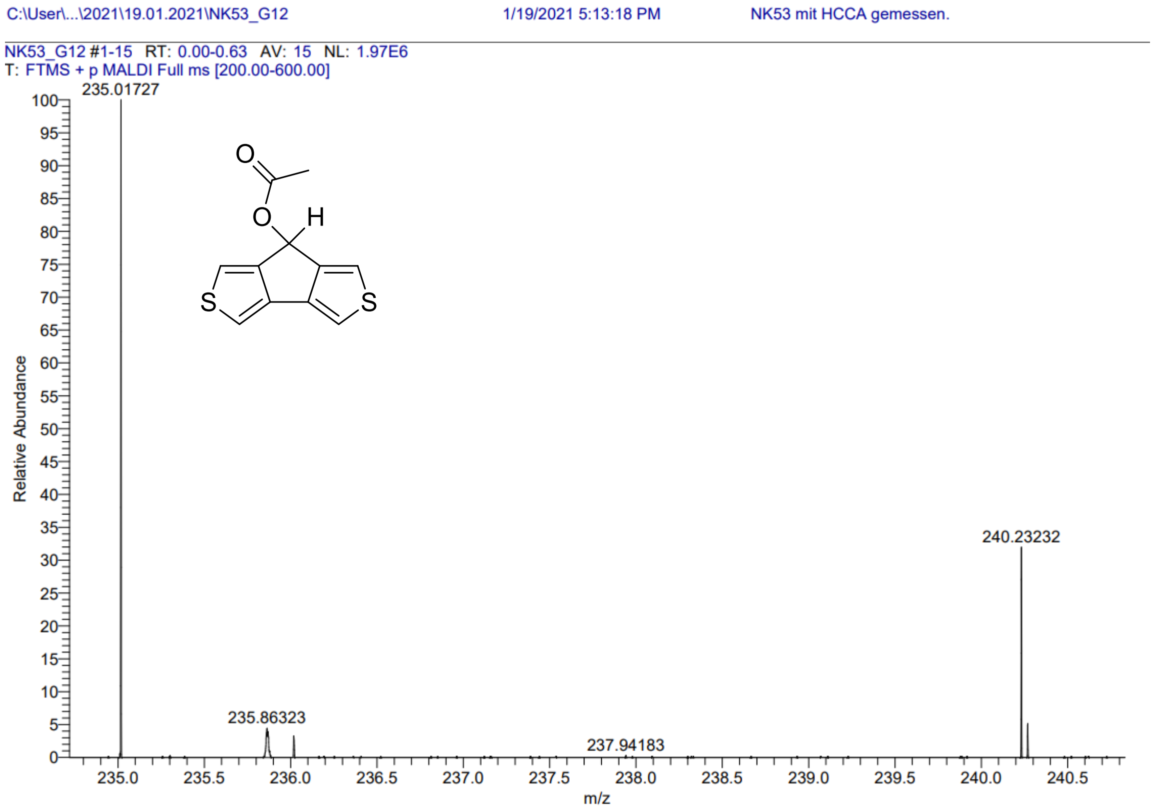

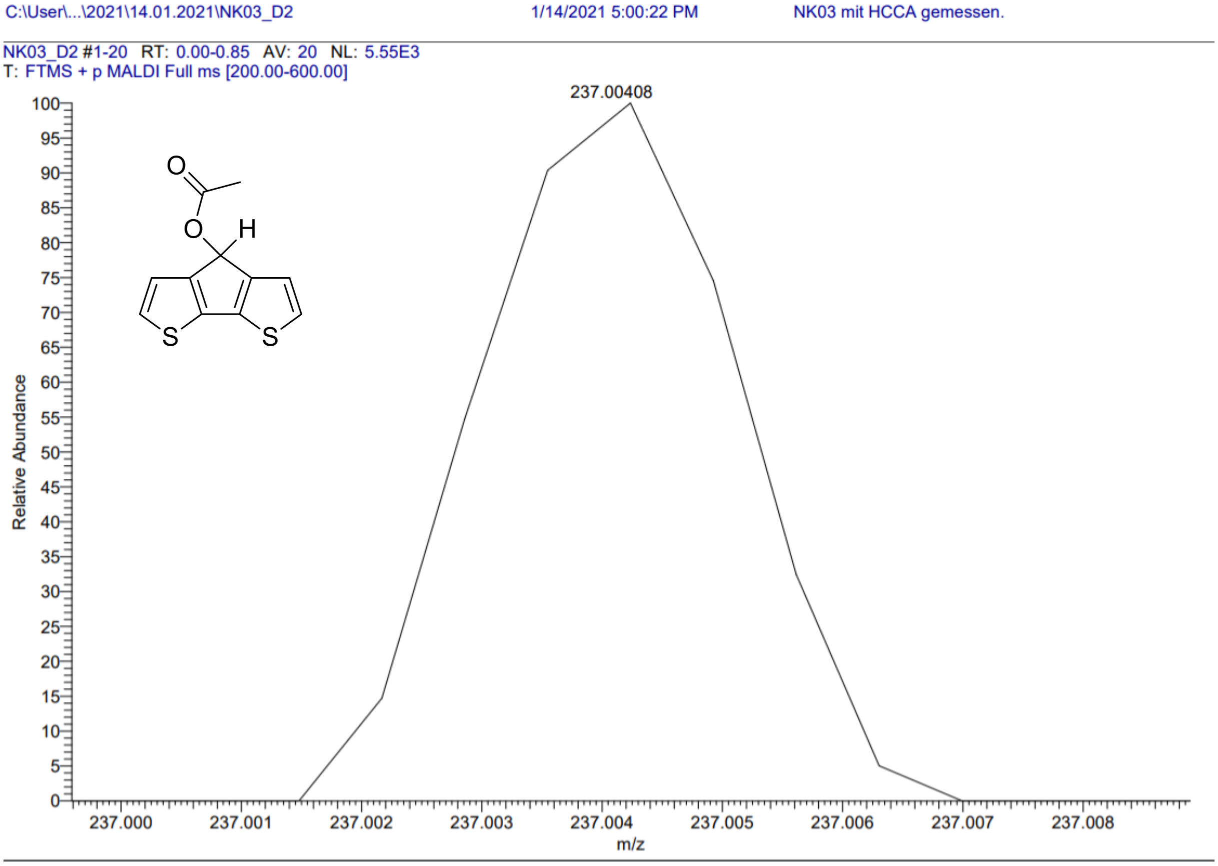

C:\User\...\2021\14.01.2021\NK03_D2
1/14/2021 5:00:22 PM
NK03 mit HCCA gemessen.
NK03_D2 #1-20 RT: 0.00-0.85 AV: 20 NL: 5.55E3
T: FTMS + p MALDI Full ms [200.00-600.00]
237.00408
Relative Abundance
m/z

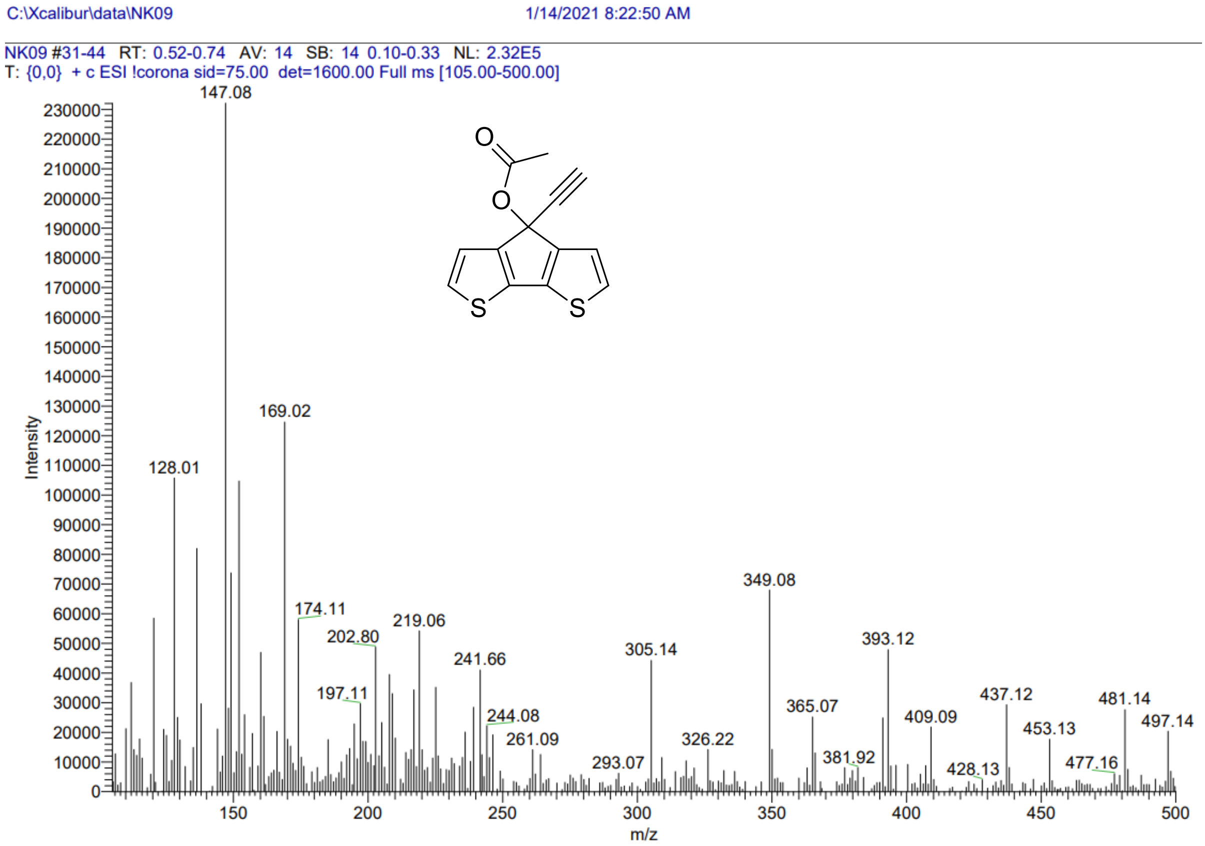

C:\Xcalibur\data\NK09
1/14/2021 8:22:50 AM
NK09 #31-44 RT: 0.52-0.74 AV: 14 SB: 14 0.10-0.33 NL: 2.32E5
T: {0,0} + c ESI !corona sid=75.00 det=1600.00 Full ms [105.00-500.00]
147.08
169.02
128.01
174.11
202.80
219.06
241.66
197.11
244.08
261.09
293.07
305.14
326.22
349.08
365.07
381.92
393.12
409.09
428.13
437.12
453.13
477.16
481.14
497.14
Intensity
m/z

NK22_D6 #1-17 RT: 0.00-0.72 AV: 17 NL: 7.90E4
T: FTMS + p MALDI Full ms [200.00-600.00]

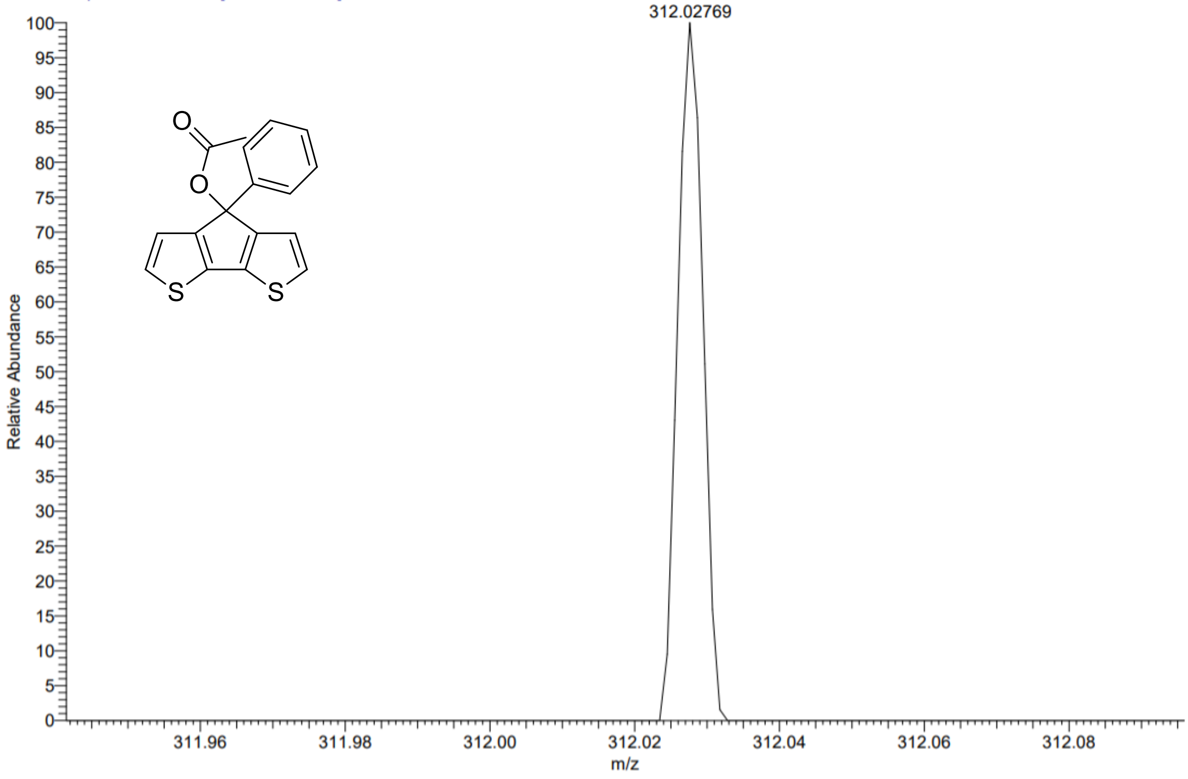

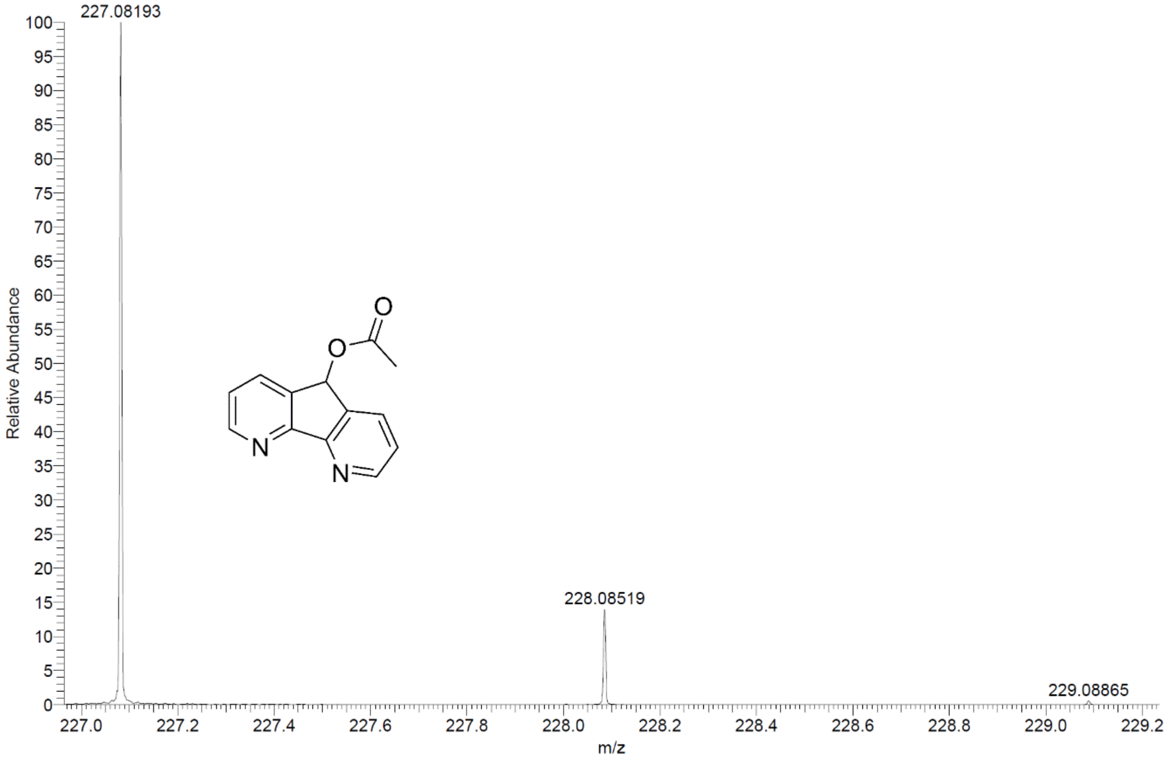

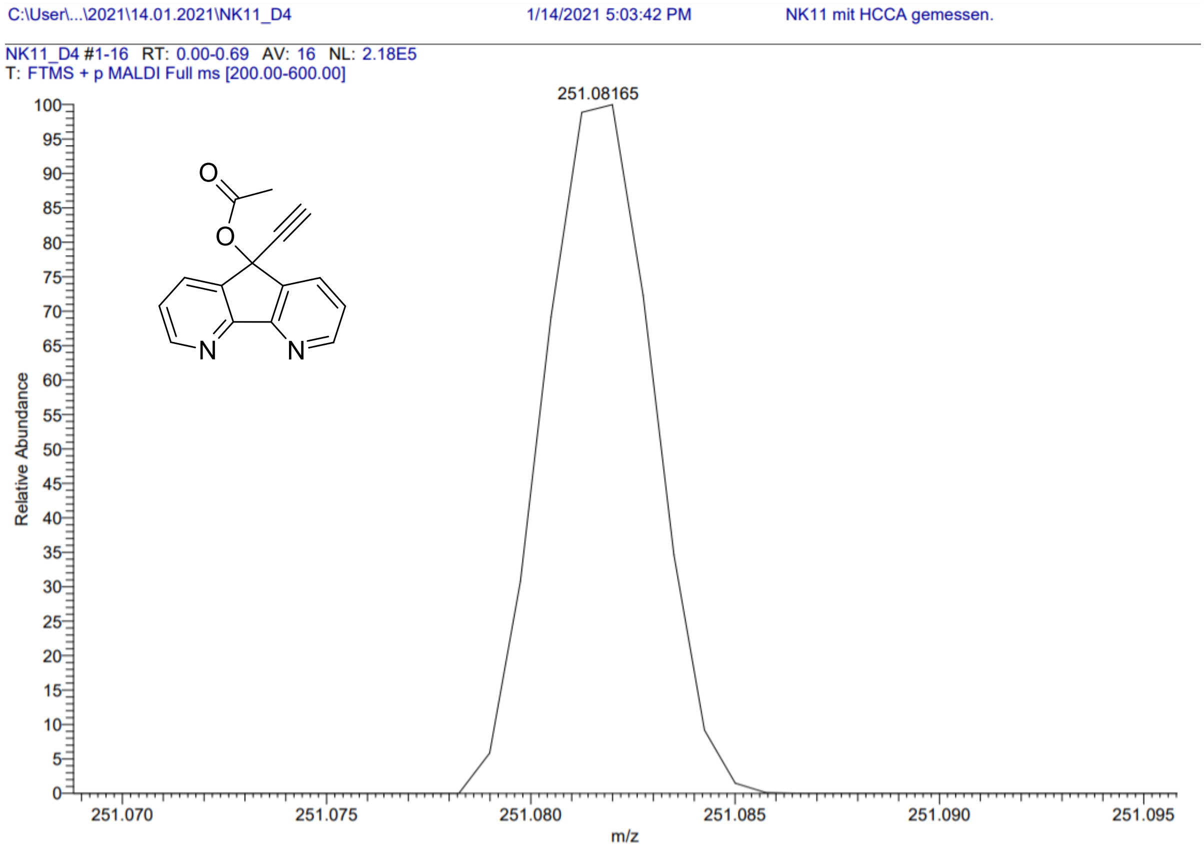
C:\User\...\2021\14.01.2021\NK11_D4
1/14/2021 5:03:42 PM
NK11 mit HCCA gemessen.
NK11_D4 #1-16 RT: 0.00-0.69 AV: 16 NL: 2.18E5
T: FTMS + p MALDI Full ms [200.00-600.00]
251.08165
Relative Abundance
m/z
N
N
O
O

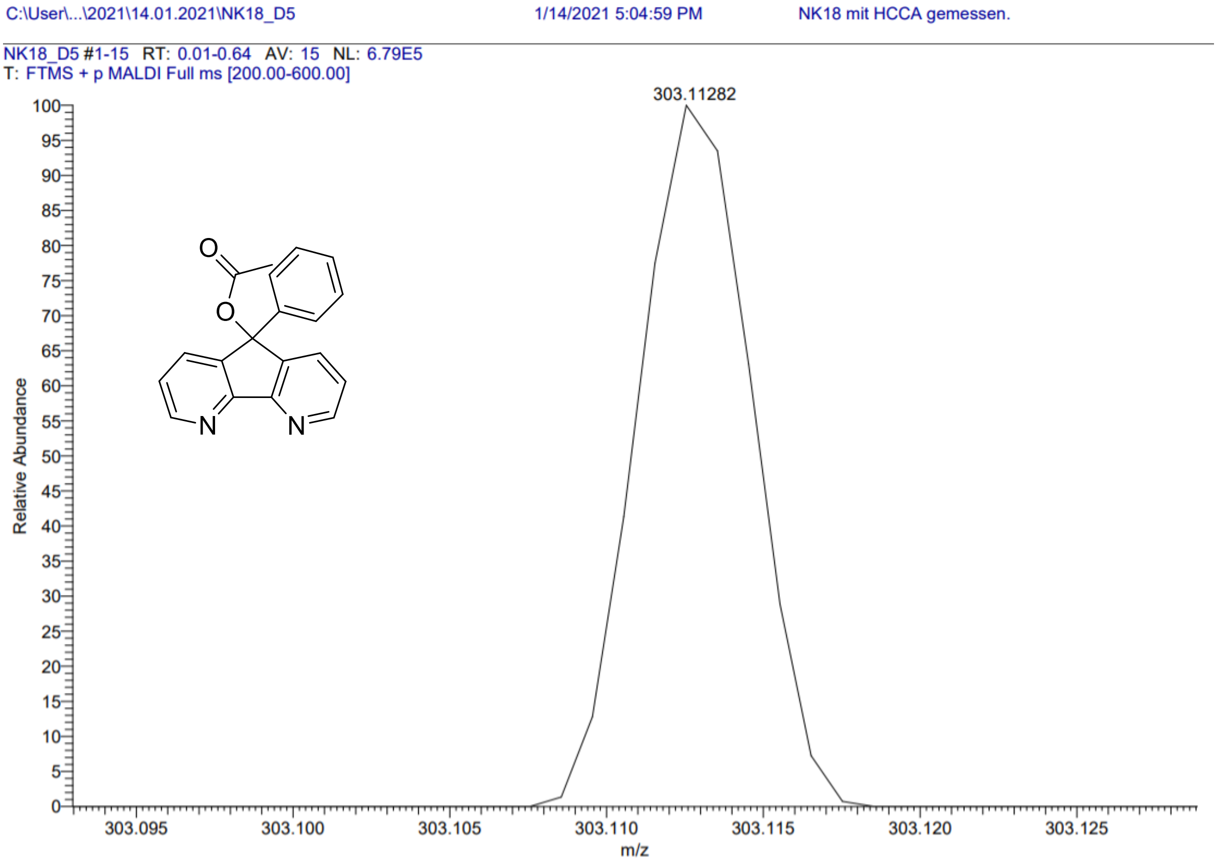
C:\User\...\2021\14.01.2021\NK18_D5
1/14/2021 5:04:59 PM
NK18 mit HCCA gemessen.
NK18_D5 #1-15 RT: 0.01-0.64 AV: 15 NL: 6.79E5
T: FTMS + p MALDI Full ms [200.00-600.00]
303.11282
Relative Abundance
m/z
N
N
O
O

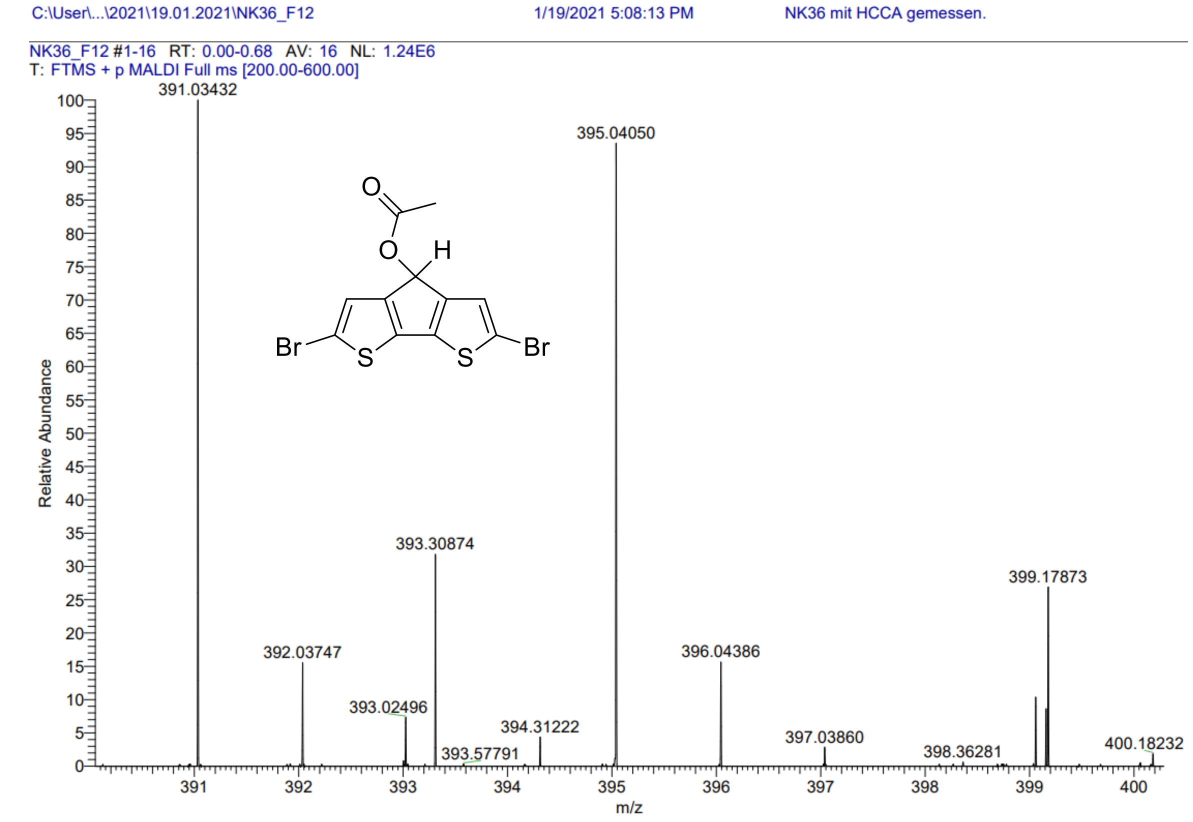

C:\User\...\2021\19.01.2021\NK36_F12
1/19/2021 5:08:13 PM
NK36 mit HCCA gemessen.
NK36_F12 #1-16 RT: 0.00-0.68 AV: 16 NL: 1.24E6
T: FTMS + p MALDI Full ms [200.00-600.00]
391.03432
395.04050
393.30874
399.17873
392.03747
396.04386
393.02496
394.31222
393.57791
397.03860
398.36281
400.18232
Relative Abundance
m/z
O
O
H
Br
S
S
Br

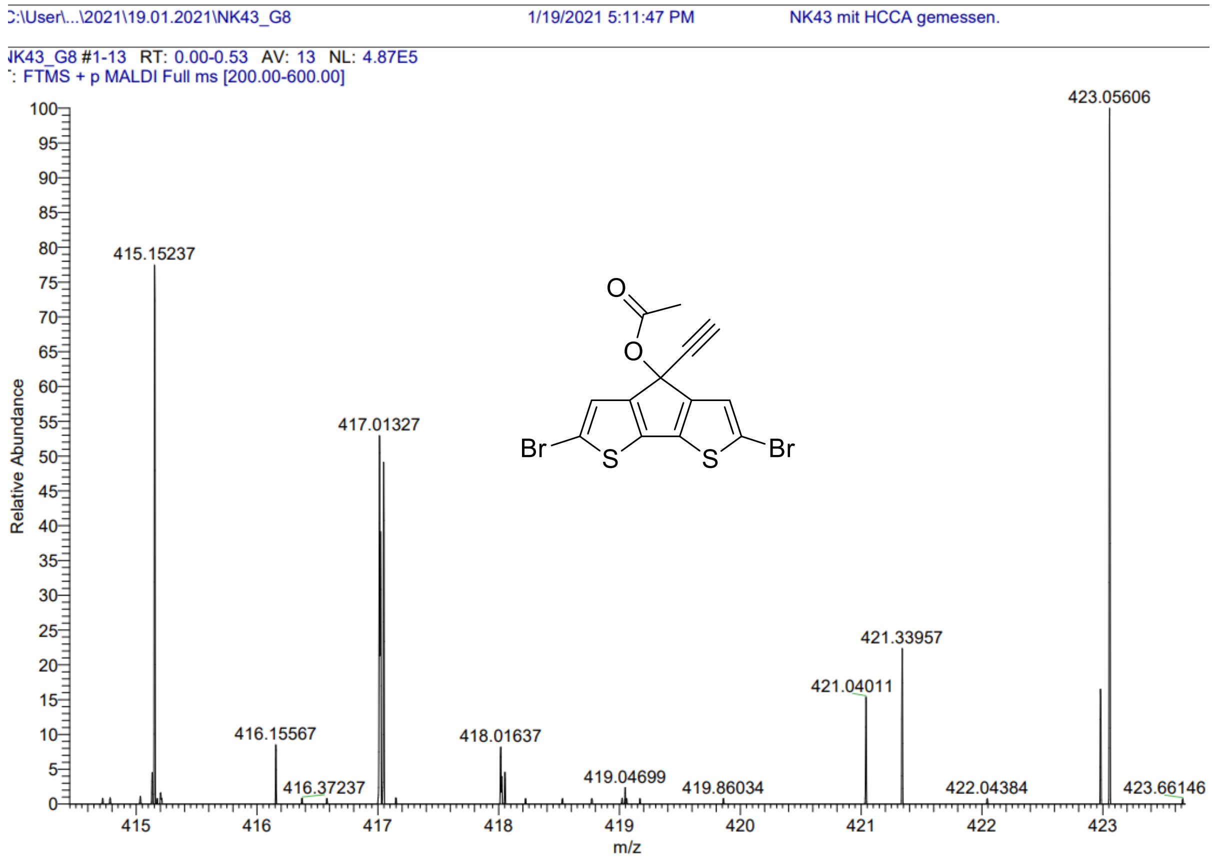

C:\User\...\2021\19.01.2021\NK43_G8
1/19/2021 5:11:47 PM
NK43 mit HCCA gemessen.
NK43_G8 #1-13 RT: 0.00-0.53 AV: 13 NL: 4.87E5
T: FTMS + p MALDI Full ms [200.00-600.00]
423.05606
415.15237
417.01327
421.33957
421.04011
416.15567
418.01637
419.04699
416.37237
419.86034
422.04384
423.66146
Relative Abundance
m/z
O
O
Br
S
S
Br

C:\User\...\2021\19.01.2021\NK41_G4 1/19/2021 5:10:34 PM NK41 mit HCCA gemessen.

NK41_G4 #1-12 RT: 0.00-0.50 AV: 12 NL: 9.74E5
T: FTMS + p MALDI Full ms [200.00-600.00]

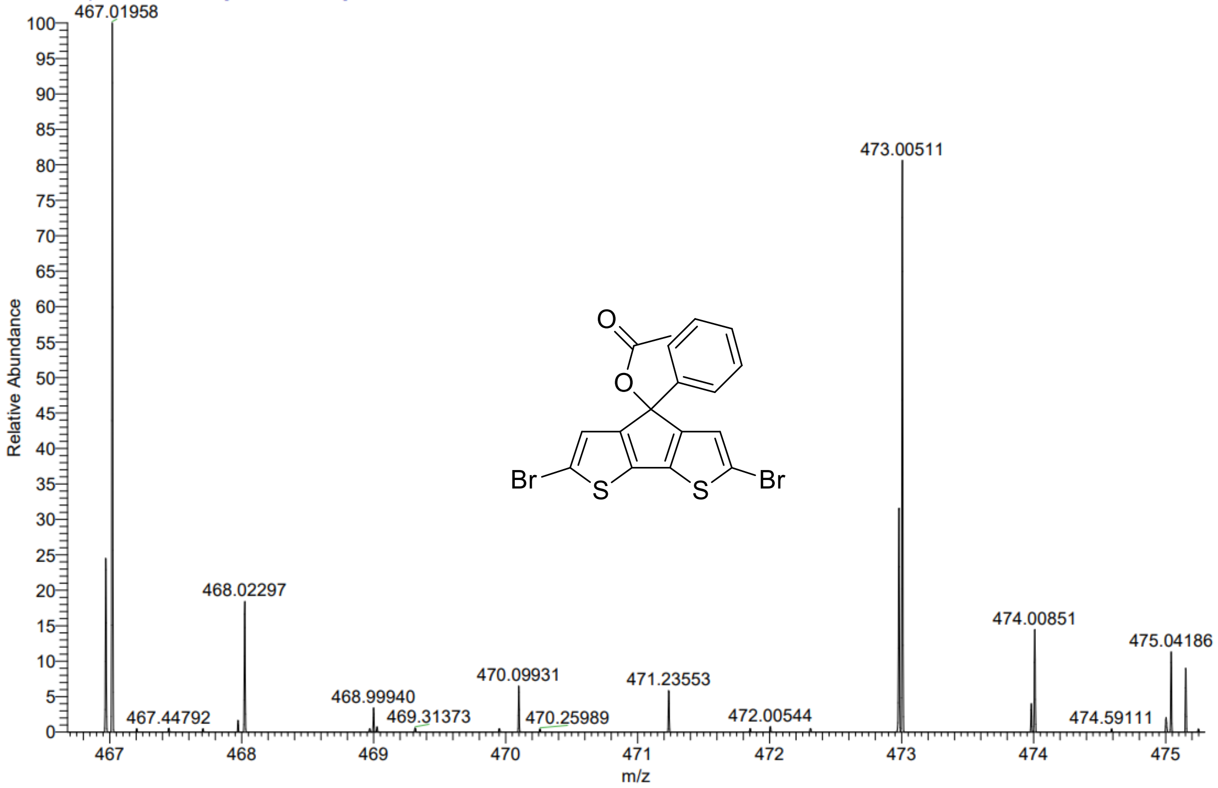

C:\User\...\2021\14.01.2021\NK49_D11 1/14/2021 6:12:52 PM NK49 mit HCCA gemessen.

NK49_D11 #1-14 RT: 0.00-0.59 AV: 14 NL: 5.89E5
T: FTMS + p MALDI Full ms [200.00-600.00]

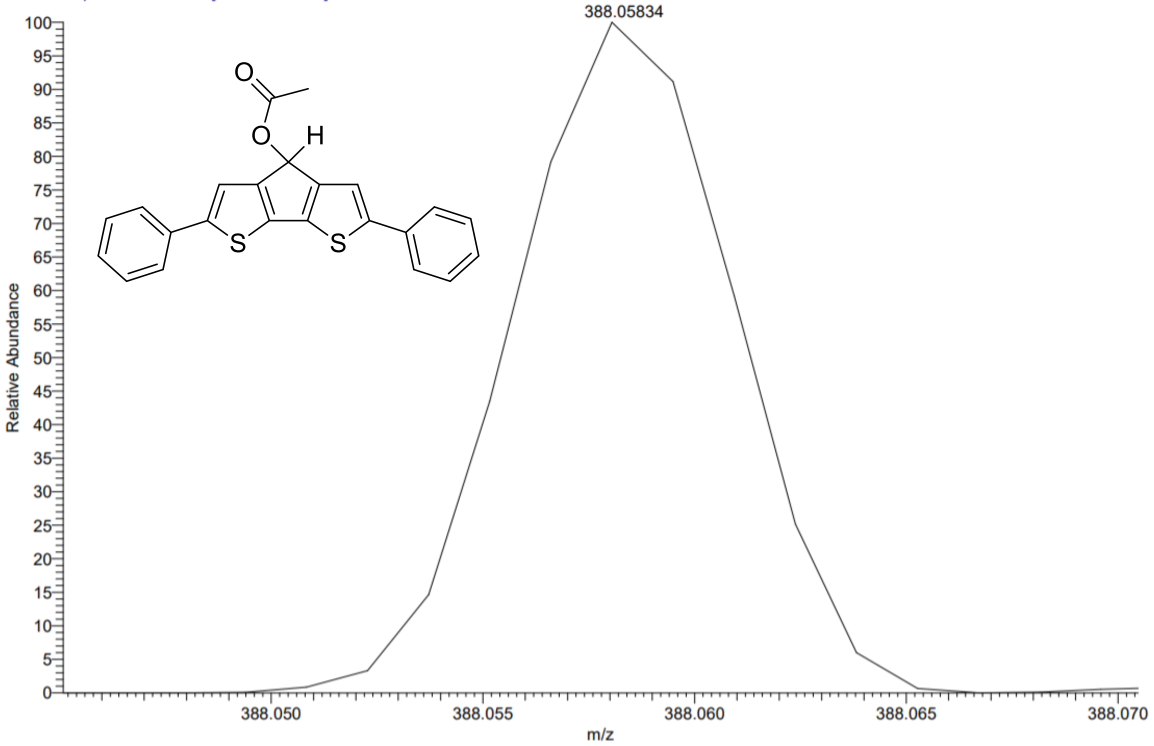

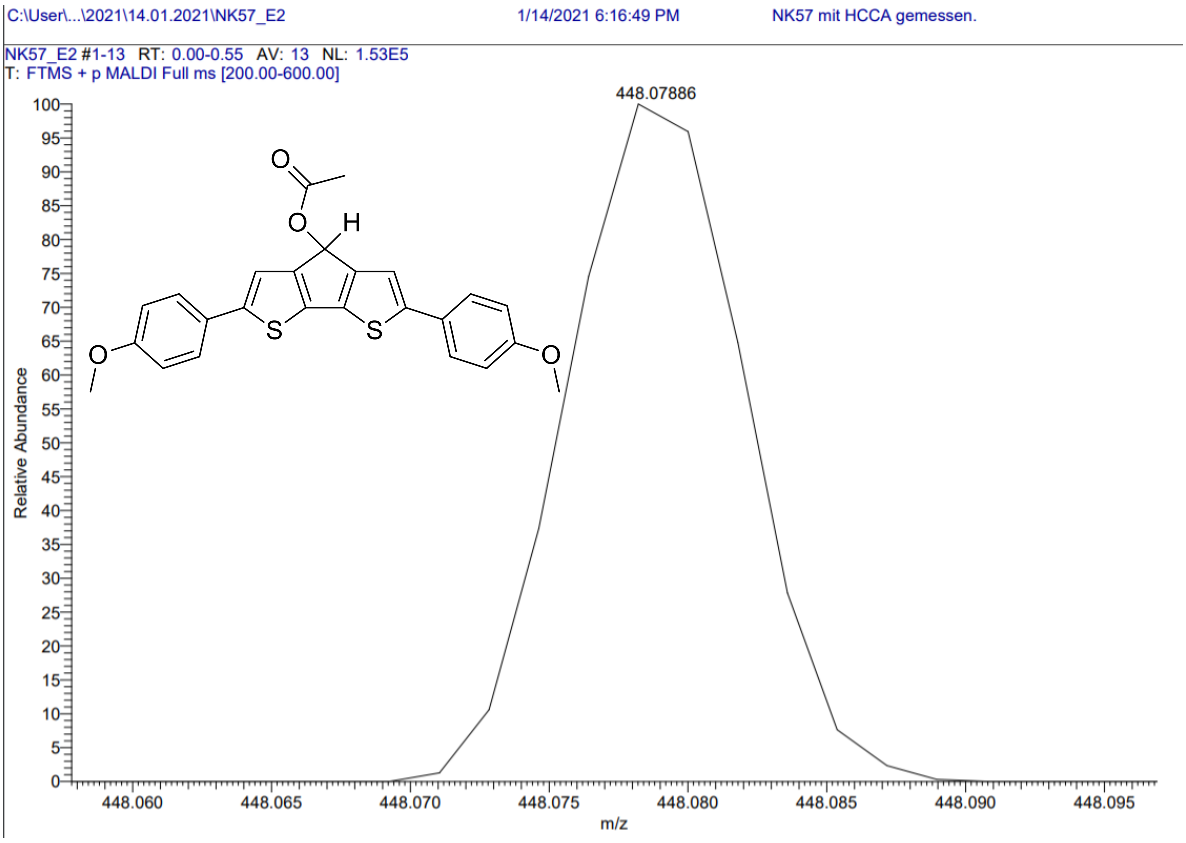
C:\User\...\2021\14.01.2021\NK57_E2
1/14/2021 6:16:49 PM
NK57 mit HCCA gemessen.
NK57_E2 #1-13 RT: 0.00-0.55 AV: 13 NL: 1.53E5
T: FTMS + p MALDI Full ms [200.00-600.00]
448.07886
Relative Abundance
m/z
448.060
448.065
448.070
448.075
448.080
448.085
448.090
448.095
O
O
H
S
S
O
O

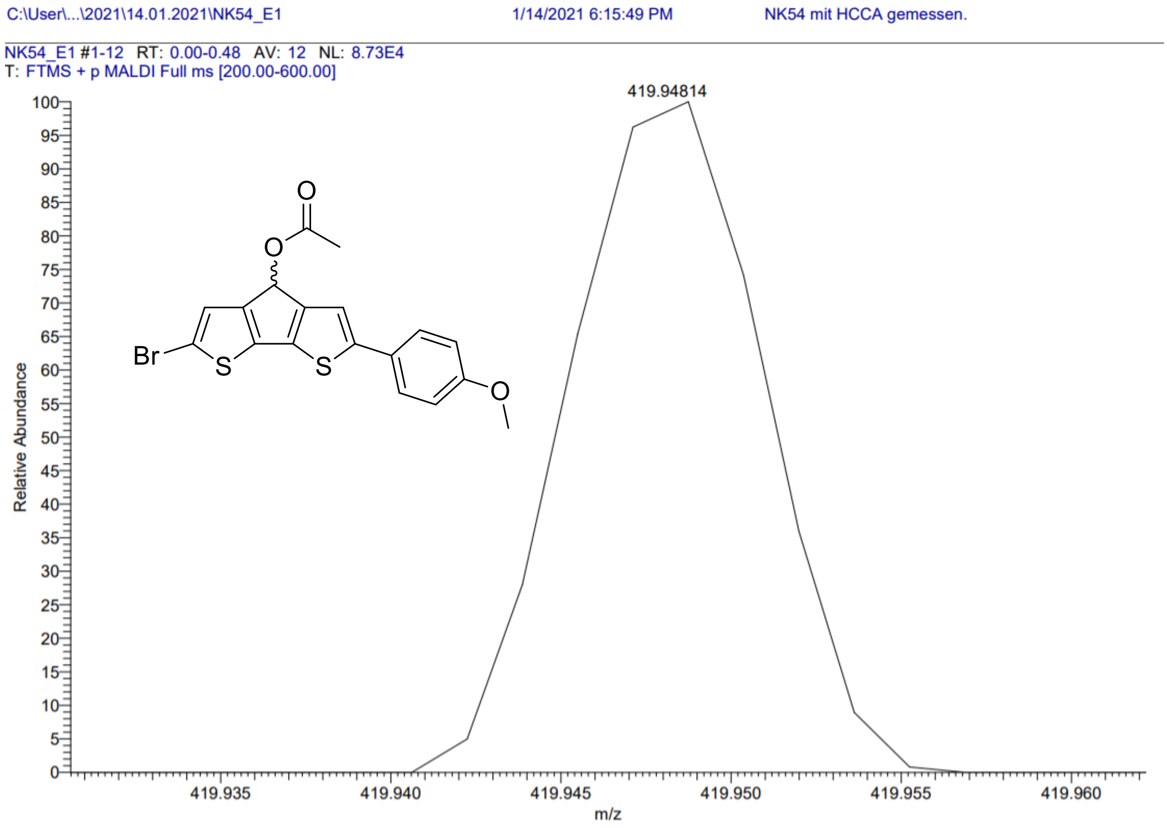
C:\User\...\2021\14.01.2021\NK54_E1
1/14/2021 6:15:49 PM
NK54 mit HCCA gemessen.
NK54_E1 #1-12 RT: 0.00-0.48 AV: 12 NL: 8.73E4
T: FTMS + p MALDI Full ms [200.00-600.00]
419.94814
Relative Abundance
m/z
419.935
419.940
419.945
419.950
419.955
419.960
O
O
Br
S
S
O

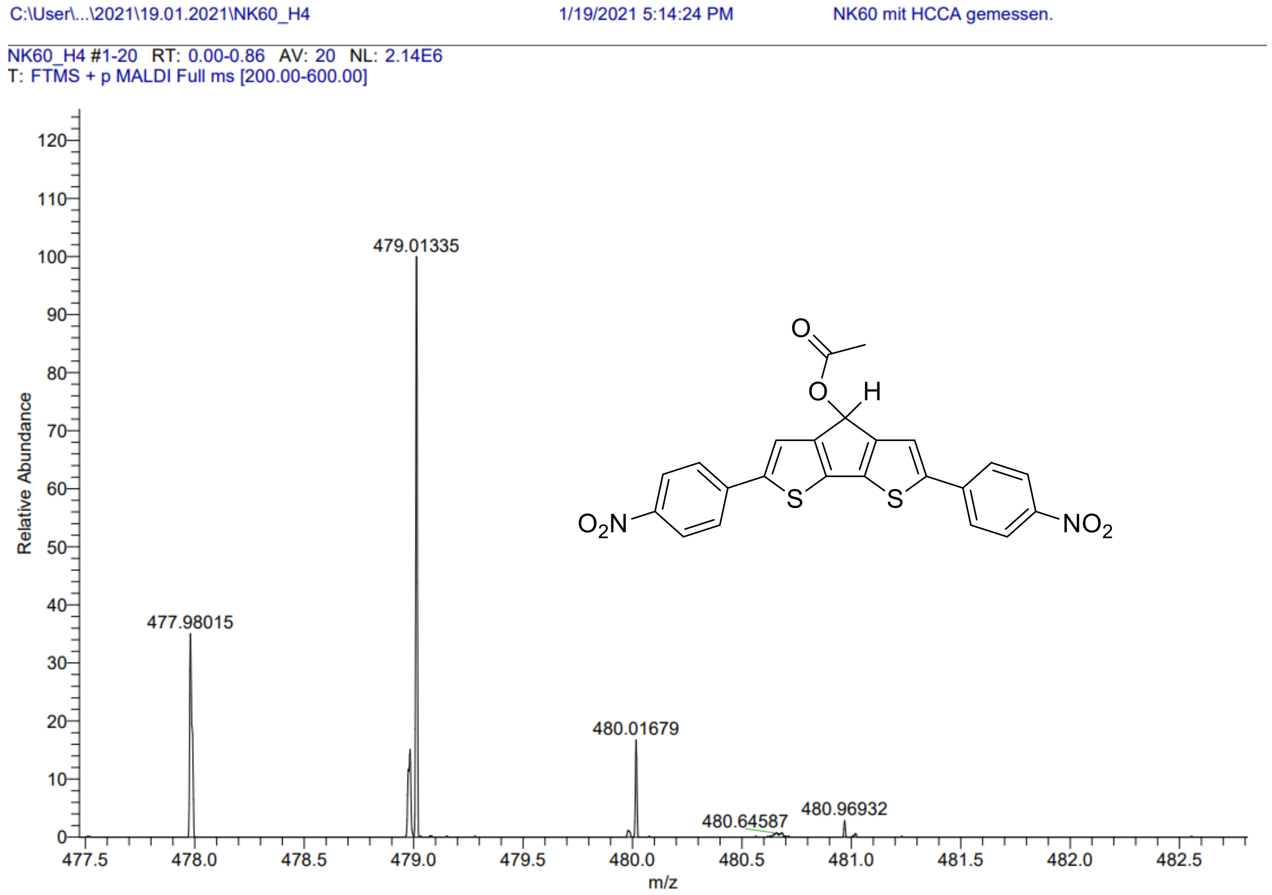

7. References

[1] M. Reinfelds, V. Hermanns, T. Halbritter, J. Wachtveitl, M. Braun, T. Slanina, A. Heckel, *ChemPhotoChem* **2019**, *3*, 441–449.

[2] C.-J. Sun, P.-F. Wang,H. Wang, B.-H. Han, *Polym. Chem.* **2016**, *7* (31), 5031–5038.

[3] J. Shi, W. Zhao, L. Xu, Y. Kan, C. Li, J. Song, H. Wang, *J. Phys. Chem. C* **2014**, *118* (15), 7844–7855.

[4] A. Rajca, M. Miyasaka, M. Pink, H. Wang, S. Rajca, *J. Am. Chem. Soc.* **2004**, *126* (46), 15211–15222.

[5] P. Willot; L. De Cremer, G. Koeckelberghs, *Macromol. Chem. Phys.* **2012**, 213 (12), 1216–1224.

[6] M. Reinfelds, J. v. Cosel, K. Falahati, C. Hamerla, T. Slanina, I. Burghardt, A. Heckel, *Chem. Eur. J.* **2018**, *24*, 13026-13035.

[7] A. T. Buck, C. L. Beck, A. H. Winter, *J. Am. Chem. Soc.* **2014**, *136*, 8933-8940.
